# Correction: Cranial Osteology of *Fona herzogae* (Dinosauria: Thescelosaurinae) from the Mussentuchit Member of the Cedar Mountain Formation

**DOI:** 10.1371/journal.pone.0359389

**Published:** 2026-09-28

**Authors:** The PLOS One Staff

## Notice of Republication

This article was republished on September 11, 2026, to correct errors in the Supporting Information files that were introduced during the syndication process. The publisher apologizes for the errors. Please download this article again to view the correct version. The originally published, uncorrected article and the republished, corrected articles are provided here for reference.

## Supporting information

S1 File Originally published, uncorrected article. (PDF)

S2 File Republished, corrected article. (PDF)
