# Supplementary material for: Cranial Osteology of Fona herzogae (Dinosauria: Thescelosaurinae) from the Mussentuchit Member of the Cedar Mountain Formation
Source: PLoS One. 2026 Aug 12;21(8):e0353169. doi: 10.1371/journal.pone.0353169 (PMC13565367; doi:10.1371/journal.pone.0353169)
Supplement: Supplementary file 1 [file pone.0353169.s038.docx]

**Supporting Information 2 phylogenetic matrix updates**

**Changes to the phylogenetic matrix of Avrahami et al. (2024)**

- **Character 365** from Avrahami et al., 2024 (originally Dieudonné et al. (2021) character number 310) was removed due to ambiguity in character definition and difficulty in consistent interpretation.
- **Character 367** from Avrahami et al., 2024 (originally based on Boyd (2015) character 227 and Scheetz (1999) character 115was removed because it violates logical fallacy Types 2B and 5 of Simões et al. (2017). Tibial midshaft cross-sections occur along a continuum from circular to elliptical to triangular, with many SBEDOs exhibiting an intermediate rounded-triangular condition. Additionally, cross-sectional geometry varies depending on the position along the shaft, making this character difficult to reproduce without explicitly defined measurement locations.
- **Character 410**, representing a newly defined scapular character, was added to the matrix and coded across the taxon sample. This character is derived from observations illustrated in Figure 5I of Avrahami et al. (38). Three additional figures explicitly illustrating the character and its discrete states are provided at the end of this document.
- Two new characters from Fonseca et al. (2024) were added, coding for the presence of ossified clavicles (**Character 411**) and clavicle morphology (**Character 412**). The character description of 411 was modified to apply only to articulated specimens or specimens represented by complete postcrania, as the absence of clavicles cannot be confidently assessed in disarticulated or incomplete material.
- **Character 406** was redefined and recoded across the taxon list to to better reflect the original intent of Weishampel et al. (2003) “*Plesiomorphically, ossified tendons are longitudinally arranged along the epaxial region of the dorsal, sacral and caudal vertebrae (0). This condition is also seen in Hypsilophodon, Gasparinisaura, Parksosaurus, Thescelosaurus, Dryosaurus and Tenontosaurus. Iguanodon and Camptosaurus, however, have ossified epaxial tendons arranged in a double-layered lattice (1).”* Therefore, many taxa here are miscoded, as they do not show the lattice arrangements [1] which is the condition in iguanodontians. To clarify this, the character description was amended to focus on the arrangement of the tendons along the dorsal and pelvic regions, as this arrangement can change along the series particularly between the pelvic region which can show a lattice pattern, and the tail, which can have the tendons arranged in a parallel sheath.

**Changes to taxon character states**

***Koreanosaurus***

- **Character 215** [Less than ten cervical vertebrae (0), ten or more cervical vertebrae (1)] was changed from **1 to 0** based on Huh et al. (2010) who note a maximum of seven cervical vertebrae for this taxon.
- **Character 219** [Postaxial cervical vertebrae, epipophyses on the postzygapophyses somewhere within the neck: absent (0), present (1) ] was changed from **0 to 1** based on the fourth cervical of KDRC-BB2; the right postzygapophysis bears a raised tubercle near its contact with the prezygapophysis of the fifth cervical.
- **Character 222** [Cervical vertebrae, evolution of central length throughout the series: central length remains approximately the same or decrease posteriorly (0), increase posteriorly (1). ] was changed from **1 to 0**. Only a small number of cervical centra are sufficiently preserved for measurement. Cervical vertebra KDRC-BB3, recovered ~2 m from KDRC-BB2 and likely representing the same individual, measures ~3.0 cm in length. The fifth cervical of the articulated cervical block of KDRC-BB2 measures ~3.5 cm, whereas the terminal cervical preserved in the articulated chest block is only ~2.4 cm long, indicating no consistent posterior increase in centrum length.
- **Character 223** [ Dorsal neural spines: arise anteriorly or are centered over centrum (0), posteriorly positioned on centrum (1).] was changed from **0 to 1** based on several dorsal vertebrae of KDRC-BB2 in which the caudal margin of the neural spine extends posterior to the caudal margin of the centrum.
- **Character 229** [ Dorsal ribs, distal anteroposterior thickening: absent (0), present (1). ] was changed from **1 to 0**, as none of the preserved dorsal ribs exhibit distal anteroposterior thickening.
- **Character 231** [Ossified intercostal plates: absent (0); present (1).] was changed from **0 to ?** due to the disarticulated and incomplete nature of the material and the possible subadult status noted by Huh et al. (2011, p. 10).
- **Character 237** [Sacral neural spines lean: posteriorly (0), anteriorly (1).] and **character 238** [Sacral vertebrae, transverse thickening and increase in rugosity near dorsal margins of neural spines: absent (0); present (1).] were changed from **? to 0** based on personal observation of KDRC-BB3.
- **Character 257** [Scapula, length of blade relative to minimum width: short and broad, ratio of length to width 5.7 or less (0); ratio of length to width 6 to 7.3 (1); elongate, ratio of 7.5 or greater (2). ] was changed from **? to 0** based on Huh et al. (2011), personal observations, and measurements derived from 3D-scanned scapulae.
- **Character 260** [Scapula, supraglenoid fossa: absent (0); present (1).] was changed from **? to 0**; the left scapula of KDRC-BB2 lacks a supraglenoid fossa.
- **Character 264** [Scapula and coracoid, relative contributions to the glenoid fossa in transverse width: scapula wider (0); subequal (1); coracoid wider (2).] was changed from **? to 1** based on 3D scans and direct observation of the left scapula of KDRC-BB2.
- **Character 269** [Ovoid fossa positioned anteroventral to the glenoid fossa on the coracoid absent (0), present (1).] was changed from **0 to 1**. Both scapulocoracoids of KDRC-BB2 preserve a lateral fossa positioned slightly ventral to and between the glenoid fossa and the coracoid foramen.
- **Character 272** [Proportions of humerus and scapula: scapula longer than humerus (0); subequal (1); humerus substantially longer than the scapula (2). ] was changed from **2 to 1**. Comparison of 3D surface models indicates that the humerus of KDRC-BB2 is only marginally longer than the scapula.
- **Character 348** [Femur, anterior extension of the greater trochanter beyond the femoral head: almost inexistent (0), shortly expanded and thick anteriorly (1), moderately to very elongated (2) ] was changed from **? to 2** based on the left femur of KDRC-BB3.
- **Character 352** [Intertrochanteric notch between the lesser and greater trochanters on the femur: absent (0), present (1).] was changed from **0 to 1** based on Huh et al. (2011, p. 17): *“A vertical cleft separates the trochanters.”*
- **Character 355** [Insertion scar of m. Caudifemoralis longus on femur: extends from fourth trochanter onto medial surface of femoral shaft (0), widely separated from fourth trochanter and restricted to the medial surface of the femoral shaft (1).] changed from **? To 0** based on personal observation of KDRC-BB3 and KDRC-BB1
- **Character 363** [Femur, posterolateral condyle position and size in ventral view: positioned relatively laterally and slightly narrower in width than the medial condyle (0), strongly inset medially, reduced in width relative to medial condyle (1)] was changed from **0 to 1** based on 3D scans and personal observations of KDRC-BB3 and KDRC-BB1.
- **Character 372** [ Proximal tibia, lateral fibular condyle from an anteroposterior view: gradually and merges with the shaft distally (0), defines an abrupt overhanging buttress with sub-horizontal ventral margin above the shaft (1).] was changed from **? to 0** based on personal observation of KDRC-BB3.
- **Character 405** [Epaxial ↑ ossified tendons absent (0), present (1).] Changed from **? To 1.** Multiple likely ossified tendons were found embedded in the matrix surrounding several element blocks of KDRC-BB2 and KDRC-BB3. These include a series of 2 or 3 parallel tendons in the matrix near the cranial side of the sacral vertebra block, a series of 3 or 4 tendons in the space between the neural spine and the left transverse process of the 6th? dorsal, and a single isolated tendon ventral to the left transverse process of the 4th dorsal.

***Hypsilophodon***

- **Character 260** [Scapula, supraglenoid fossa: absent (0); present (1)] was reverted from **1 to** **0**. This character was previously miscoded based on an MOR 054 cast labeled *Hypsilophodon* that actually represents *Orodromeus*.

***Albertadromeus***

- **Character 404** [Ossified hypaxial ↓ tendons along the tail absent (0), present (1)] was changed from **0 to ?** due to the lack of preserved caudal vertebrae or associated caudal ossified tendons on *Albertadromeus syntarsus* (TMP2009.037.0044).
- **Character 406** [Epaxial ↑ tendons of the dorsal and sacral (not caudal) vertebrae: longitudinally arranged into a single layer (0), arranged in a double-layered rhomboidal lattice (1)] was changed from **1 to ?** due to the inability to assess the orientation of the ossified tendon architecture on the dorsal vertebrae of TMP2009.037.0044. See comment from Brown et al. (2013) “*Due to the fragmentary and dissociated nature of these tendons, the architecture of the tendon network cannot be determined.*”

***Zephyrosaurus***

- **Character 404** [Ossified hypaxial ↓ tendons along the tail absent (0), present (1)] changed from **? to 0** based on personal observation of YPM 56695; no ossified tendons are preserved on any of the 11 caudal vertebrae.

***Orodromeus***

- **Character 405** [Epaxial ↑ ossified tendons absent (0), present (1)] for *Orodromeus* changed from **0 to 1**, and **character 406** [Epaxial ↑ tendons of the dorsal and sacral (not caudal) vertebrae: longitudinally arranged into a single layer (0), arranged in a double-layered rhomboidal lattice (1)] changed from **- to 0** based on observations reported in Hannebaum (2025) MS thesis.

***Thescelosaurus neglectus***

- **Character 406** [Epaxial ↑ tendons of the dorsal and sacral (not caudal) vertebrae: longitudinally arranged into a single layer (0), arranged in a double-layered rhomboidal lattice (1)] was changed from **0 to 1** based on the pelvic region of NCSM 15728, which preserves a double-lattice tendon arrangement.

***Oryctodromeus***

- **Character 406** [Epaxial ↑ tendons of the dorsal and sacral (not caudal) vertebrae: longitudinally arranged into a single layer (0), arranged in a double-layered rhomboidal lattice (1)] was changed from **0 to ?** based on difficulty interpreting the exact arrangement of tendons near the pelvic region. Although tendons are clearly arranged in parallel along the tail, the tendons around the pelvis are more difficult to confirm, and some near the right lateral side of the first few caudal vertebrae seem to show a lattice arrangement, although this is difficult to confirm due to the possibility of taphonomic disruption.

***Haya***

- **Character 13** [Contact between premaxilla and lacrimal: absent (0), present (1).] was changed from 0 to 0/1 based on MPC-D 170920.
- **Character 16** [Premaxilla, premaxillary palate: strongly arched, forming a deep, concave palate (0), horizontal or only gently arched (1) ] was changed from ? to 1 based on MPC-D 170920.
- **Character 18** [Premaxilla-vomeral head, ventral contact: present (0), absent, ventrally excluded by midline contact between maxillae (1) ] was changed from ? to 0 based on IGM 100/2017.
- **Character 61** [Posterior maxillary process on the medial side of the jugal: straight to modestly arched medially (0), anteromedially projected and arched (1) ] was changed from ? to 0 based on MPC-D 170920.
- **Character 62** [Ectopterygoid facet on the medial surface of the jugal consists of: an abbreviated, deep groove (0), a rounded scar (1).] was changed from ? to 0 based on MPC-D 210723.
- **Character 96** [Synovial socket for the head of the laterosphenoid positioned: along the frontal-postorbital contact (0), only in the postorbital (1), only in the frontal (2), no synovial joint for the laterosphenoid present (3).] was changed from ? to 0 based on MPC-D 210723.
- **Character 130** [Alveolar foramina (‘special foramina’) medial to maxillary/dentary tooth rows: absent (0); present (1). ] was changed from ? to 1 based on MPC-D 170920.
- **Character 155** [Pterygoid, contact with its counterpart: absent, the basicranium is mostly exposed in ventral view, (0) present, the basicranium is mostly obscured in ventral view by an interpterygoid contact formed by the palatal and/or quadrate rami (1) ] was changed from ? to 1 based on IGM 100 2017.

***Parksosaurus***

- **Character 406** [Epaxial ↑ tendons of the dorsal and sacral (not caudal) vertebrae: longitudinally arranged into a single layer (0), arranged in a double-layered rhomboidal lattice (1)] was changed from **0 to ?** based on lack of preserved tendons along the dorsal or sacral regions.

***Jeholosaurus***

- **Character 406** [Epaxial ↑ tendons of the dorsal and sacral (not caudal) vertebrae: longitudinally arranged into a single layer (0), arranged in a double-layered rhomboidal lattice (1)] was changed from state **1 to 0** based on this comment from Han et al. (2012) “*The tendons lack a distinct lattice-like arrangement and appear to be arranged in linear bundles. There is no evidence for the tendons extending onto anterior dorsals or cervical vertebrae in any specimen*.”

***Minimocursor***

- **Character 406** [Epaxial ↑ tendons of the dorsal and sacral (not caudal) vertebrae: longitudinally arranged into a single layer (0), arranged in a double-layered rhomboidal lattice (1)] changed state **1 to 0** based on this comment from Manitkoon et al. (2023) *“Ossified tendons run longitudinally along the lateral surfaces of the neural spines of the dorsal vertebrae (they are clearly visible from the dv4) and terminate at the first sacral vertebra (Figures 2, 7 and 10). They are long, slender, round in cross section, and appear to be to be parallel to each other.*

***Talenkauen***

- **Character 404** [Ossified hypaxial ↓ tendons along the tail absent (0), present (1)] changed from **? To 1** based on Novas et al. (2004) figure 1 B and C, showing an arrangement of ossified tendons along the dorsal series that closely mirror the condition in the pelvis of *T. neglectus*, and those preserved in *Haya griva*

***Macrogryphosaurus***

- **Character 404** [Ossified hypaxial ↓ tendons along the tail absent (0), present (1)] for *Macrogryphosaurus* changed from **? To 0** based on figure 9A of Rozadilla et al. (2020) figure 9 which shows no ossified tendons in association with the caudal series.
- **Character 405** [Epaxial ↑ ossified tendons absent (0), present (1)] for *Macrogryphosaurus* changed from **? To 1** based on Rozadilla et al. (2020) which notes ossified epaxial tendons on posterior dorsal and sacral vertebrae

***Mahuidacursor***

- **Character 404** [Ossified hypaxial ↓ tendons along the tail absent (0), present (1)] was changed from **? to 1** based on the presence of ossified tendons mentioned in Cruzado-Caballero et al. (2019)

***Yueosaurus***

- **Character 404** [Ossified hypaxial ↓ tendons along the tail absent (0), present (1)] and **Character 405** [Epaxial ↑ ossified tendons absent (0), present (1)] changed from **? To 0** based on Zheng et al. (2012) statement “*No ossified tendons are preserved in Yueosaurus (ZMNH M8620)”*

***Lesothosaurus***

- **Character 404** [Ossified hypaxial ↓ tendons along the tail absent (0), present (1)] was changed from **1 to 0** based on Baron et al. (2017) where Figure 4D does not indicate any ossified hypaxial tendons along the caudal (tail) series. Additionally, there is no mention of ossified tail tendons in the text except for fragments in articulation with the dorsal vertebrae.

***Leaellynasaura***

- **Character 406** [Epaxial ↑ tendons of the dorsal and sacral (not caudal) vertebrae: longitudinally arranged into a single layer (0), arranged in a double-layered rhomboidal lattice (1)] changed from **? To 0** based on Sharp et al. (2017) SVP Poster

***Valdosaurus***

- **Character 406** [Epaxial ↑ tendons of the dorsal and sacral (not caudal) vertebrae: longitudinally arranged into a single layer (0), arranged in a double-layered rhomboidal lattice (1)] was changed from state **1 to 0** based on figure 8 of Barrett (2016) and text comment “*Those tendons that are preserved in their natural orientations (e.g. those preserved alongside Cd26–29) extend subparallel to each other, and the overlapping trellis-like arrangement seen in ankylopollexian ornithopods (e.g. Norman, 1980, 1986) was absent.”*

***Camptosaurus***

- **Character 404** [Ossified hypaxial ↓ tendons along the tail absent (0), present (1)] and **character 405** [Epaxial ↑ tendons of the dorsal and sacral (not caudal) vertebrae: longitudinally arranged into a single layer (0), arranged in a double-layered rhomboidal lattice (1)] changed from **? to 1** due to presence of ossified tendons documented in (Carpenter and Wilson, 2008).

***Rhabdodon_p.***

- **Character 404** [Ossified hypaxial ↓ tendons along the tail absent (0), present (1)], **character 405** [Epaxial ↑ tendons of the dorsal and sacral (not caudal) vertebrae: longitudinally arranged into a single layer (0), arranged in a double-layered rhomboidal lattice (1)], and **character 406** [Epaxial ↑ tendons of the dorsal and sacral (not caudal) vertebrae: longitudinally arranged into a single layer (0), arranged in a double-layered rhomboidal lattice (1)] changed from **? to 1** due to presence of ossified tendons figured in Chanthasit (2010) and this statement “*The ossified tendons are usually found fragmentary and it can hardly be recognized whether they belong to Rhabdodon. The best occurrence of ossified tendons in Rhabdodon has been described by Chanthasit et al. (in review) on the basis of the ossified tendons found associated with the new partial skeleton of Rhabdodon priscus from the Vitrolles Couperigne locality. The ossified tendons are angular to rounded rods with striations along the length. They are distributed along the vertebral column, particularly concentrated on the posterior dorsal vertebrae and sacrum. Both epaxial and hypaxial ossified tendons are present in the caudal series where we can see them on neural spines (Figure 4.21) and chevrons (See fig.8G Chanthasit et al., in review) respectively. Moreover, as in figure 4.21, the presence of the ossified tendons on the mid-posterior caudal vertebrae indicates that the ossified tendons of Rhabdodon probably were present up to the posterior part of the vertebral column. The arrangements of ossified tendons are difficult to discern. They seem to be divided in two groups of tendons. The first group is represented by longitudinal 75 tendons running down the vertebrae with a small degree of inclination and the second group represented by relatively short tendons crossing obliquely as present on the neural spine.”*

***Goyocephale***

- **Character 404** [Ossified hypaxial ↓ tendons along the tail absent (0), present (1)] changed from **? to 1** based on Perle et al. (1982) plate 45 figure 3 C and D

***Micropachycephalosaurus***

- **Character 404** [Ossified hypaxial ↓ tendons along the tail absent (0), present (1)] and 405 changed from **? to 0** based on this comment from Butler and Zhao (2009) *“Similar fusiform tendons have been reported for Micropachycephalosaurus (Maryanska et al., 2004: p. 471; ossified tendons were mentioned by Maryanska [1990: p. 575] but she did not state whether they were fusiform or not); however, these are not present on the preserved caudals, could not be located elsewhere amongst the holotype material, were not mentioned by Dong (1978), and have not been figured or described by Dong (1978) or Maryanska et al. (2004)”*

**Supplementary Figure S2.1: Annotation of Character 410 (medial scapular tuberosity).**
Representative 3D scapulae illustrating the absence (State 0; blue, upper panel) and presence (State 1; red, lower panels) of a tuberosity or swelling on the medial surface of the scapular neck, near the narrowest portion of the blade and opposite the glenoid ridge. All sampled orodromines lack this feature, whereas it is present in multiple thescelosaurines and some iguanodontians. Adjacent magnified views show the scapular neck rotated 90° to emphasize the region of interest. An embedded 7-second video shows the direction of lighting rotation 360° to aid visualization of surface relief. *Zephyrosaurus* YPM 56695 exhibits minor taphonomic distortion in the region of interest; however, no tuberosity or ridge is evident. Expression of this feature is weakly developed or ambiguous in *Thescelosaurus* and *Parksosaurus* and could reasonably be interpreted as absent; recoding both taxa as absent in exploratory TNT analyses did not alter overall tree topology. Elements are not to scale**.**

**References**

Avrahami HM, Makovicky P, Tucker RT, Zanno LE. 2024. A New Semi-Fossorial Thescelosaurine Dinosaur from The Cenomanian-age Mussentuchit member of the Cedar Mountain Formation, Utah. The Anatomical Record: 1–65.

Baron MG, Norman DB, Barrett PM. 2017. Postcranial anatomy of *Lesothosaurus diagnosticus* (Dinosauria: Ornithischia) from the Lower Jurassic of southern Africa: implications for basal ornithischian taxonomy and systematics. Zoological Journal of the Linnean Society 179:125-168.

Barrett PM. 2016. A new specimen of *Valdosaurus canaliculatus* (Ornithopoda: Dryosauridae) from the Lower Cretaceous of the Isle of Wight, England.

Boyd CA. 2015. The systematic relationships and biogeographic history of ornithischian dinosaurs. PeerJ 3:e1523.

Brown CM, Evans DC, Ryan MJ, Russell AP. 2013. New data on the diversity and abundance of small-bodied ornithopods (Dinosauria, Ornithischia) from the Belly River Group (Campanian) of Alberta. Journal of Vertebrate Paleontology 33:495-520.

Butler RJ, Zhao Q. 2009. The small-bodied ornithischian dinosaurs Micropachycephalosaurus hongtuyanensis and Wannanosaurus yansiensis from the Late Cretaceous of China. Cretaceous Research 30:63-77.

Carpenter K, Wilson Y. 2008. A new species of *Camptosaurus* (Ornithopoda: Dinosauria) from the Morrison Formation (Upper Jurassic of Dinosaur National Monument, Utah, and a biomechanical analysis of its forelimb. Annals of Carnegie Museum 76:227-263.

Chanthasit P. 2010. The ornithopod dinosaur *Rhabdodon* from the Late Cretaceous of France: anatomy, systematics and paleobiology. In: Université Claude Bernard-Lyon I.

Cruzado-Caballero P, Gasca JM, Filippi LS, Cerda IA, Garrido AC. 2019. A new ornithopod dinosaur from the Santonian of Northern Patagonia (Rincón de los Sauces, Argentina). Cretaceous Research 98:211-229.

Dieudonné P-E, Cruzado-Caballero P, Godefroit P, Tortosa T. 2021. A new phylogeny of cerapodan dinosaurs. Historical Biology:1-21.

Fonseca AO, Reid IJ, Venner A, Duncan RJ, Garcia MS, Müller RT. 2024. A comprehensive phylogenetic analysis on early ornithischian evolution. Journal of Systematic Palaeontology 22:2346577.

Han F, Barrett PM, Butler RJ, Xu X. 2012. Postcranial Anatomy of *Jeholosaurus shangyuanensis* (Dinosauria, Ornithischia) from the Lower Cretaceous Yixian Formation of China. Journal of Vertebrate Paleontology 32:1370-1395.

Hannebaum ZJD. 2025. EXPLORING ORODROMINE VARIATION IN THE TWO MEDICINE-JUDITH RIVER-BELLY RIVER CLASTIC WEDGE IN MONTANA AND ALBERTA. In: MONTANA STATE UNIVERSITY Bozeman.

Huh M, Lee D-G, Kim J-K, Lim J-D, Godefroit P. 2010. A new basal ornithopod dinosaur from the Upper Cretaceous of South Korea. Neues Jahrbuch für Geologie und Paläontologie Abhandlungen 2010:in press.

Manitkoon S, Deesri U, Khalloufi B, Nonsrirach T, Suteethorn V, Chanthasit P, Boonla W, Buffetaut E. 2023. A New Basal Neornithischian Dinosaur from the Phu Kradung Formation (Upper Jurassic) of Northeastern Thailand. Diversity 15:851.

Novas FE, Cambiaso AV, Ambrosio A. 2004. A new basal iguanodontian (Dinosauria, Ornithischia) from the Upper Cretaceous of Patagonia. Ameghiniana 41:75-82.

Perle A, Maryanska T, Osmólska H. 1982. Goyocephale lattimorei gen. et sp. n., a new flat-headed pachycephalosaur (Ornlthlschia, Dinosauria) from the Upper Cretaceous of Mongolia. Acta Palaeontologica Polonica 27.

Rozadilla S, Cruzado-Caballero P, Calvo JO. 2020. Osteology of Ornithopod Macrogryphosaurus gondwanicus (Dinosauria, Ornithischia) from the Upper Cretaceous of Patagonia, Argentina. Cretaceous Research 108:104311.

Scheetz RD. 1999. Osteology of *Orodromeus makelai* and the phylogeny of basal ornithopod dinosaurs. In: Citeseer.

Sharp AC, Siu K, Rich T. 2017. Revealing the skeleton of the polar dinosaur *Leaellynasaura amicagraphica* using synchrotron computed tomography. In: Society of Vertebrate Paleontology 77th Annual Meeting, Meeting Program and Abstracts.

Simões TR, Caldwell MW, Palci A, Nydam RL. 2017. Giant taxon‐character matrices: quality of character constructions remains critical regardless of size. Cladistics 33:198-219.

Weishampel DB, Jianu CM, Csiki Z, Norman DB. 2003. Osteology and phylogeny of *Zalmoxes* (ng), an unusual euornithopod dinosaur from the latest Cretaceous of Romania. Journal of Systematic Palaeontology 1:65-123.

Zheng W, Jin X, Shibata M, Azuma Y, Yu F. 2012. A new ornithischian dinosaur from the cretaceous Liangtoutang formation of Tiantai, Zhejiang Province, China. Cretaceous Research 34:208-219.
