# Supplementary material for: Correction: Cranial Osteology of Fona herzogae (Dinosauria: Thescelosaurinae) from the Mussentuchit Member of the Cedar Mountain Formation
Source: PLoS One. 2026 Sep 28;21(9):e0359389. doi: 10.1371/journal.pone.0359389 (PMC13618937; doi:10.1371/journal.pone.0359389)
Supplement: Supplementary file 2 [file pone.0359389.s002.pdf]

PLOS One

RESEARCH ARTICLE

Cranial Osteology of *Fona herzogae* (Dinosauria: Thescelosaurinae) from the Mussentuchit Member of the Cedar Mountain Formation

Haviv M. Avrahami[1,2¤☯*], **Lindsay E. Zanno**[1,2☯]

1 Paleontology, North Carolina Museum of Natural Sciences, Raleigh, North Carolina, United States of America, **2** Department of Biological Sciences, North Carolina State University, Raleigh, North Carolina, United States of America

☯ These authors contributed equally to this work.
¤ Current Affiliation: Paleontology, Museum of the Rockies, Bozeman, Montana, United States of America
* avrahamihm@gmail.com, haviv.avrahami@montana.edu

Check for updates

OPEN ACCESS

Citation: Avrahami HM, Zanno LE (2026) Cranial Osteology of *Fona herzogae* (Dinosauria: Thescelosaurinae) from the Mussentuchit Member of the Cedar Mountain Formation. PLoS One 21(8): e0353169. https://doi.org/10.1371/journal.pone.0353169

Editor: Jun Liu, Chinese Academy of Sciences, CHINA

Received: July 26, 2025

Accepted: June 19, 2026

Published: August 12, 2026

Copyright: This is an open access article, free of all copyright, and may be freely reproduced, distributed, transmitted, modified, built upon, or otherwise used by anyone for any lawful purpose. The work is made available under the Creative Commons CC0 public domain dedication.

Data availability statement: All data necessary to reproduce and evaluate the findings of this study are included within the paper and its Supporting Information files, including the revised phylogenetic matrix, character change documentation, supplemental figures, and

Abstract

The North American thescelosaurine ornithischian *Fona herzogae* (Cenomanian Mussentuchit Member of the Cedar Mountain Formation) is represented by one of the largest samples of isolated cranial elements among early diverging neornithischians. Here, we present a comprehensive cranial description of *F. herzogae*, utilizing advanced digitization and visualization techniques to reconstruct individual elements in high resolution with a particular focus on taxonomically and phylogenetically significant morphology. The abundance of disarticulated cranial material permits comprehensive documentation of individual elements from multiple orientations, substantially expanding the known cranial anatomy of *F. herzogae* and improving character evaluation across Thescelosauridae. We use these new anatomical details, together with targeted character revisions and firsthand recoding of *Koreanosaurus*, to improve resolution among thescelosaurid evolutionary relationships. Our analyses recover *Koreanosaurus* within Thescelosaurinae, strengthen support for the inclusion of several Asian taxa within this clade, and reinforce a biogeographic distinction between an Asian–North American Thescelosaurinae and a geographically restricted North American Orodrominae.

Introduction

Small-bodied, early diverging ornithischians (SBEDOs) constitute a grade (non-monophyletic group) of phylogenetically labile species outside of the well-established major ornithischian clades (e.g., heterodontosaurs, neoceratopsians, thyreophorans, and iguanodontians). The early evolutionary experimentation of cranial architectures in SBEDOs (Fig 1) set the stage for the wide array of head shapes seen among later-diverging, larger-bodied clades. Detailed osteological studies that target and figure individual cranial elements in disarticulation across multiple

analytical data files. The phylogenetic matrix and associated TNT files are provided as Supporting Information. High-resolution 3D surface models and CT-derived datasets associated with this study are being archived through MorphoSource under the management of the North Carolina Museum of Natural Sciences. Access to these files is restricted to qualified researchers upon reasonable request through MorphoSource and/or the corresponding repository institution.

Funding: This research is supported by the Jurassic Foundation, the Paleontological Society, the Canyonlands Natural History Association, the North Carolina Fossil Club, and the National Science Foundation under Grant Nos. FRES 1925973 & 1925884. The funders had no role in study design, data collection and analysis, decision to publish, or preparation of the manuscript.

Competing interests: NO authors have competing interests.

anatomical views are necessary to fully document the anatomy and variation of early ornithischian skulls and often require the utilization of modern CT and surface scanning techniques [1–4]. Moreover, recent advancements in digital 2D and 3D data collection methods [5] now offer enhanced resolution, precision, and objectivity in the visualization of these key features. Nonetheless, such studies remain sparse. Some historically known SBEDOs, such as *Hypsilophodon*, are exceptionally well-preserved and thoroughly documented anatomically [6,7]; however, these studies were conducted prior to the discovery of numerous new taxa that have since broadened the list of significant features. Although there are exceptional collections comprising multiple complete or nearly complete specimens of *Orodromeus* and *Haya*, the anatomy of individual elements is limited to the portions exposed in articulation, leaving much of the internal surface morphology of individual elements, as well as their articulations, underdescribed. Even when detailed osteologies utilizing CT analyses are performed, they often focus on a single individual skull rather than multiple individuals [8]. This deficit hampers our ability to perform confident anatomical comparisons (often leading to the misidentification of important features), limits our ability to construct phylogenetic characters for inferring neornithischian interrelationships, and prevents explorations of morphological variation.

The recently described thescelosaurine *Fona herzogae* is exceptional among SBEDOs, in that it represents an attritional accumulation of individuals deposited over a narrow window of time (~235,000 years), provides a nearly complete catalog of skeletal elements (many articulated or associated) across multiple individuals, and critically includes multiple individuals from one population—associated subadults from the Mini Troll locality [9] that permit confident assessments of cranial morphology. Here, we document the individual cranial elements of *Fona* in high-resolution detail with expanded anatomical descriptions and annotated osteological figures from multiple views improving comparative anatomy and phylogenetic resolution amongst early diverging neornithischians.

Materials and Methods

Specimens

The skeletal elements described and illustrated in this study (Fig 2) derive from six discrete Cenomanian-aged localities within the upper and lower units of the Mussentuchit Member of the Cedar Mountain Formation. These localities collectively span an interval of approximately 235,000 years. Locality names, stratigraphic positions, and geologic context are summarized in Figure 1 of Avrahami et al. [9], which provides a detailed overview of the temporal and depositional framework for *Fona herzogae*.

Skull elements of *Fona* from multiple specimens recovered from the Mini Troll, Karmic, and Manolo localities [9]. This figure should be used with caution for phylogenetic character coding and is likely inappropriate for characters based on proportional measurements. It represents a composite reconstruction derived from multiple specimens, and several elements and regions are incomplete (e.g., the lacrimal–jugal–maxilla junction), potentially obscuring accurate anatomical proportions. Dashed

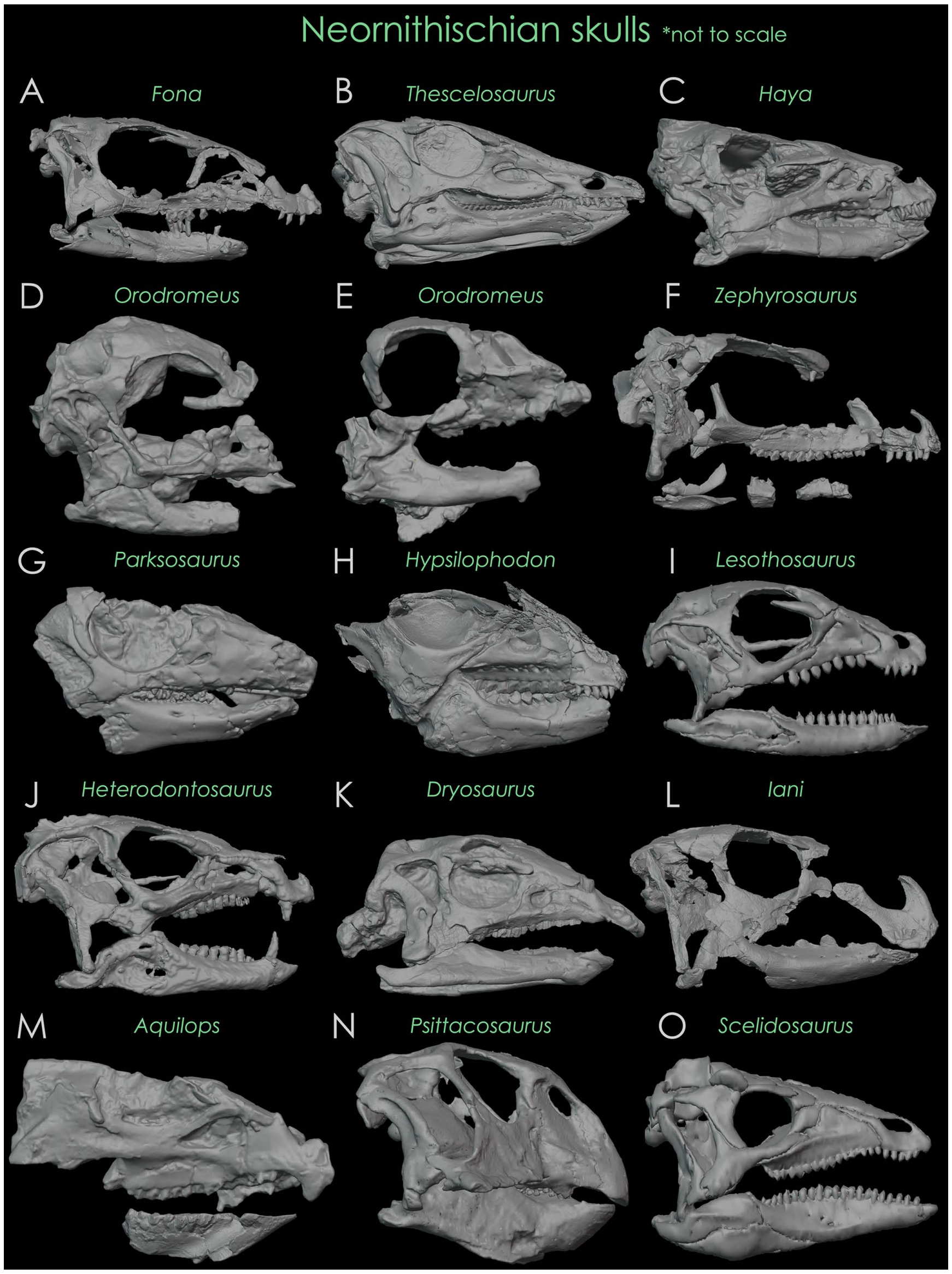

Fig 1. Examples of fifteen early diverging ornithischian skulls. (A) Composite skull of *Fona herzogae* [9]. **(B)** *Thescelosaurus neglectus* NCSM 15728. **(C)** *Haya griva* MPC-D 100/2017. **(D)** *Orodromeus makelai* MOR 294. **(E)** *Orodromeus makelai* MOR 1141. **(F)** *Zephyrosaurus schaffi* MCZ 4392. **(G)** *Parksosaurus warreni* ROM 804. **(H)** *Hypsilophodon foxii* NHMUK PV R197. **(I)** Composite skull of *Lesothosaurus diagnosticus* [10]. **(J)** Composite skull of *Heterodontosaurus tucki* [10]. **(K)** *Dryosaurus elderae* CM 3392. **(L)** *Iani smithi* NCSM 29373. **(M)** *Aquilops americanus* OMNH 34557. **(N)** *Psittacosaurus lujiatunensis* IVPP V12617. **(O)** Composite skull of *Scelidosaurus harrisonii* [10].

https://doi.org/10.1371/journal.pone.0353169.g001

lines represent the missing predentary. (Top) reconstructed skull. (Bottom) each element separated and labeled. angular (NCSM 36126), articular (NCSM 33548), basioccipital (FMNH PR 4581), basisphenoid (NCSM 36129), coronoid (NCSM 33548), dentary (NCSM 36131), ectopterygoid (NCSM 36132), frontal (NCSM 36136), jugal (NCSM 33548), lacrimal (NCSM 36172), maxilla (NCSM 36143), nasal (NCSM 36144), otoccipital (NCSM 36133), palatine (NCSM 33548), parietal (NCSM 36148), postorbital (NCSM 36149), prefrontal (NCSM 36151), premaxilla (FMNH PR 4581), prootic (NCSM

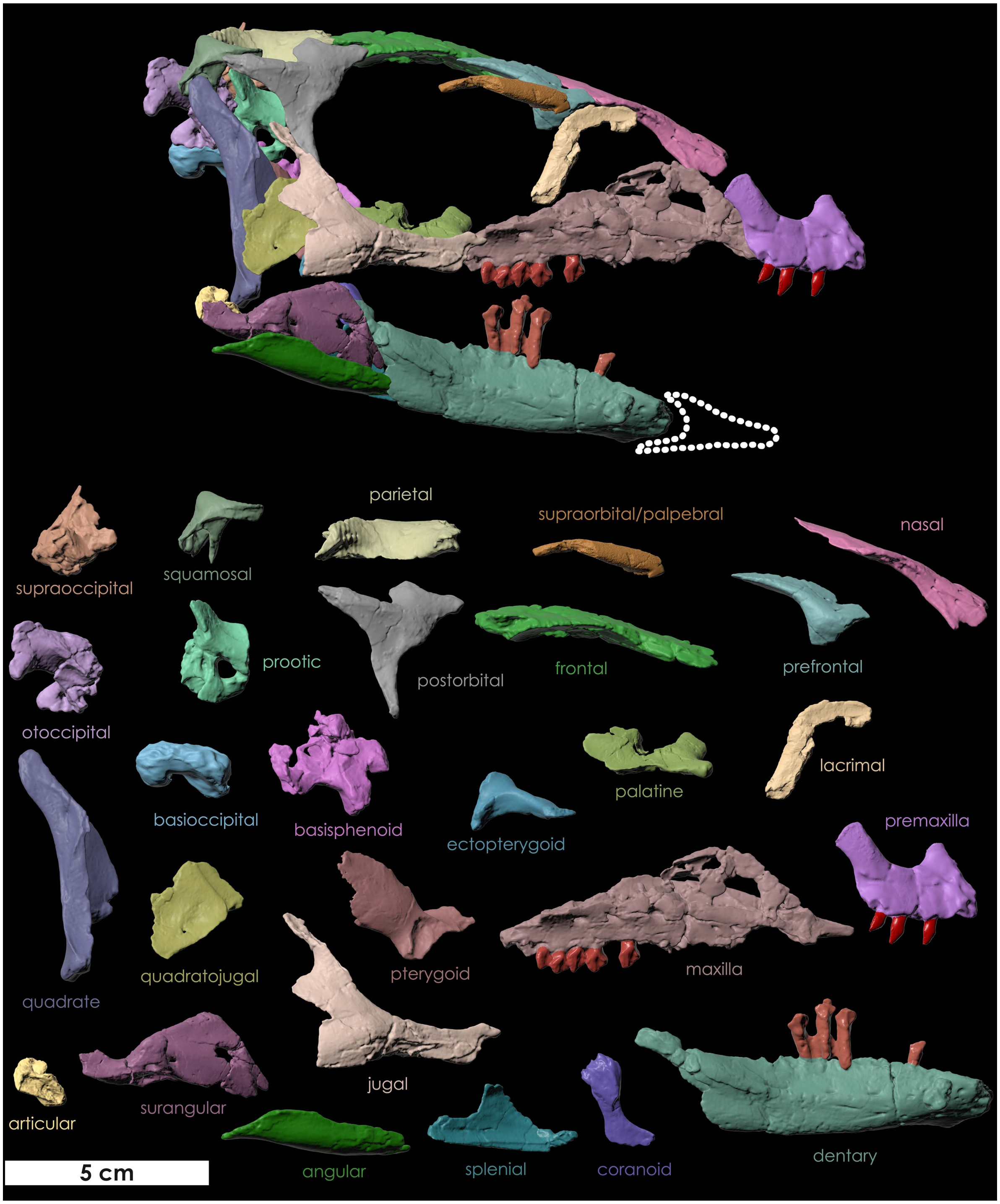

Fig 2. Cranial elements of *Fona*, modified from Figure 3 of Avrahami et al. [9].

https://doi.org/10.1371/journal.pone.0353169.g002

36156), pterygoid (NCSM 36158), quadrate (NCSM 36159), quadratojugal (NCSM 36160), splenial (NCSM 36162), squamosal (NCSM 36164), supraoccipital (NCSM 36165), supraorbital (NCSM 36147), surangular (NCSM 36166).

The Karmic (holotype) locality preserves a single individual of *Fona* (NCSM 33548), represented by a complete articulated postcranial skeleton and a partially complete, disarticulated skull. The Manolo locality preserves a single individual (FMNH PR 4581) consisting of an incomplete series of cranial and postcranial elements. The Magic Man locality, while still under active excavation, has thus far yielded a small number of postcranial elements and isolated teeth that collectively suggest the presence of a single thescelosaurine individual, though current material is insufficient for referral beyond Thescelosaurinae. Similarly, the Last Chance Theropod (LCT) locality likely represents a single disarticulated individual, also referable only to Thescelosaurinae, consisting primarily of postcranial material, isolated teeth, a single frontal, and an angular fragment, with additional material currently under active preparation. The Pit of Despair (POD) locality preserves only isolated premaxillary teeth, likewise assignable only to Thescelosaurinae.

Most significant to this study is the Mini Troll locality, which preserves two tightly co-occurring subadult individuals preserved in direct association. However, the predominantly disarticulated nature of the material means that few elements can be definitively assigned to one individual or the other. As a result, the majority of individual elements from this locality were accessioned independently with their own NCSM catalog number.

Consequently, readers will encounter numerous NCSM numbers throughout the manuscript, and individual numbers may not always be intuitively linked to specific individuals. For clarity, NCSM 33548 refers exclusively to the holotype from the Karmic locality; FMNH PR 4581 refers exclusively to the individual from the Manolo locality; and all other NCSM numbers derive from the Mini Troll locality unless explicitly noted as originating from the LCT or POD localities. A comprehensive list of specimen numbers and their locality associations is provided in the supplementary documentation of Avrahami et al. [9].

Osteological Figures

All osteology figures were produced in orthographic view using the open-source software Blender. Three-dimensional models were created using the iPhone application Metascan [5], as well as an Artec Space Spider Industrial 3D scanner. The majority of osteological figures are presented as 3D models rather than photographs, following the methodology in Debraga et al. [11]. 2D photographs were captured using the image-stacking and stitching functions of a Keyence VHX-7000 microscope. Full resolution versions of these images can be found in (S1.01–S1.37 Files).

Phylogenetic analysis

A non-time calibrated parsimony analysis and a time-calibrated Bayesian phylogenetic analysis were conducted using an updated version of the character matrix from Avrahami et al. [9]. Revisions to the matrix include: a complete re-coding of *Koreanosaurus* based on direct specimen observations and in-person 3D surface scan data collection, as well as corrections to several scoring errors identified in Avrahami et al. [9], primarily involving characters related to ossified tendons. One new scapular character was added (S1.38 File; Fig 1), derived from Figure 5I of Avrahami et al. [9], and two additional characters pertaining to the presence and morphology of the clavicle were incorporated from Fonseca et al. [12] to improve character sampling relevant to the placement of heterodontosaurids and ceratopsian outgroups. All modifications to the matrix are documented in the supporting information (S1.38 File) and are explicitly annotated as cell-level comments within the associated Excel spreadsheet (S1.39 File). The non-time calibrated parsimony analysis was run in TNT version 1.6 [13]. An invariant character was added to the first column to maintain numerical consistency between TNT and Mesquite. After the matrix was imported, "Max. trees" was set to 99,999 and RAM was set to 500 Mb. *Marasuchus* was set as the outgroup taxon and characters 23, 35, 41, 128, 160, 172, 176, 239, 252, 257, 264, and 265, were set to ordered/additive. A traditional search was performed with the TBR algorithm, setting using 1000 replicates and saving 100

trees per replication, leaving all other settings as default. 4300 trees were recovered with a score of 1812. Lastly, a strict consensus tree and a 50% majority rule tree were calculated using the default settings from all trees. The topology of these analyses is presented in supporting information (S1.40 File).

The time-calibrated Bayesian analysis was prepared in the program BEAUti 2.7.7 and run using the associated software Beast 2.7.7 [14]. Nexus files were formatted in Mesquite to be compatible with BEAUti, requiring all invariant characters to be removed. Additionally, Beast can only handle either/or polymorphic states coded as with slash "/" not simultaneous polymorphisms coded with a "&". Therefore, all "&" were converted to a slash. Next, in BEAUti, the dataset was converted into .xml files that are readable by Beast. For Beast to incorporate discrete morphological data, a series of supported packages were installed to BEAUti from its built-in package manager. These include BEASTLabs 2.0.3, cladeage (CA) 2.1.0, Morphological Models (MM) 1.2.1, Model_Selection 1.6.2, Nested Sampling (NS) 1.2.2, Optimized Relaxed Clock (ORC) 1.1.2, and sampled ancestor (SA) 2.1.1.

Six partitions were automatically created based on character state quantity, and each partition was flagged to allow the tree likelihood to use ambiguities as equally likely values for the tips. All partitions use the Lewis MK substitution model with a gamma category count of four to allow for rate heterogeneity. The clock model was set to the optimized relaxed clock and the priors model was set to Fossilized Birth-Death. *Marasuchus* was used as the outgroup.

All analyses were run with an MCMC chain length of ~100 million, storing every 5000 trees. Convergence was evaluated using Tracer 1.7.1 [15]. The maximum clade credibility tree (MCC) was created using the associated program TreeAnnotator 2.6.3 with default settings. Tree data was formatted and visualized using FigTree 1.4.4 and Adobe Illustrator.

Institutional Abbreviations

AMNH: American Museum of Natural History, New York, New York; **BMNHC:** Beijing Museum of Natural History, Beijing, China; **BYU:** Earth Sciences Museum, Brigham Young University, Provo, Utah; **CMN:** Canadian Museum of Nature, Ottawa, Canada; **DMNS**: Denver Museum of Nature & Science, Denver, Colorado; **FMNH:** Field Museum of Natural History, Chicago, Illinois; **IMNH**: Idaho Museum of Natural History, Pocatello, Idaho; **MPC-D:** Institute of Geology, Mongolian Academy of Sciences, Ulaan Baatar, Mongolia; **ROM:** Royal Ontario Museum, Toronto, Ontario, Canada; **MCZ**: Museum of Comparative Zoology, Cambridge, Massachusetts; **MOR:** Museum of the Rockies, Bozeman, Montana; **NCSM:** North Carolina Museum of Natural Sciences, Raleigh, North Carolina; **NCSU AIF:** North Carolina State University Analytical Instrumentation Facility, Raleigh, North Carolina; **MNHN:** Muséum national d'Histoire naturelle, Paris, France; **NMNH:** Smithsonian National Museum of Natural History, Washington, DC; **NHMU**: Natural History Museum of Utah, Salt Lake City, Utah; **NHMUK**: Natural History Museum, London, United Kingdom; **NMZ**: Naturhistorisches Museum der Universität Zürich, Switzerland; **OMNH**: Sam Noble Oklahoma Museum of Natural History, Norman, Oklahoma; **SMA**: Sauriermuseum Aathal, Aathal, Switzerland; **SMU**: Southern Methodist University, Dallas, Texas; **STMN**: Shandong Tianyu Museum of Nature, Pingyi, China; **TMP**: Royal Tyrrell Museum, Drumheller, Alberta, Canada; **UGS**: Utah Geological Survey, Salt Lake City, Utah; **YPM:** Yale Peabody Museum of Natural History, New Haven, Connecticut.

Results - Cranial Osteology

Dermatocranium

Premaxilla. Preservation: Two well-preserved, similarly sized, unfused left (NCSM 36152) and right (NCSM 36153) premaxillae and an equally sized, poorly preserved right (NCSM 36154) were recovered from the Mini Troll locality (Fig 3 and 4). Additionally, two unfused, slightly more dorsoventrally broad, left and right premaxillae were recovered from the Manolo Locality (FMNH PR 4581).

General shape: The premaxilla has a sub-rectangular main body and is characterized by two caudodorsally oriented processes.

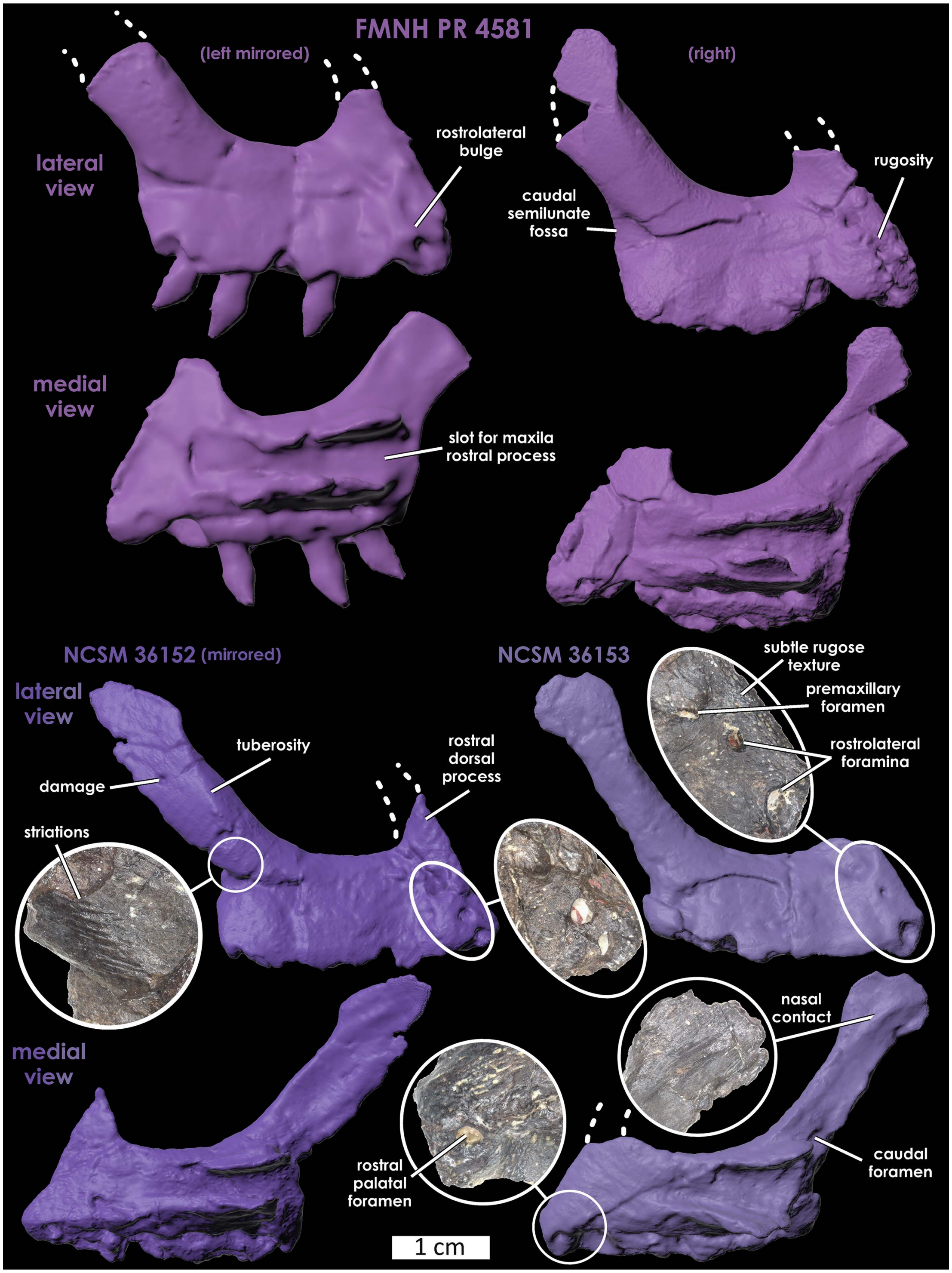

Fig 3. Premaxillae of *Fona herzogae* from multiple specimens in lateral and medial views. Dashed lines represent missing bone.

https://doi.org/10.1371/journal.pone.0353169.g003

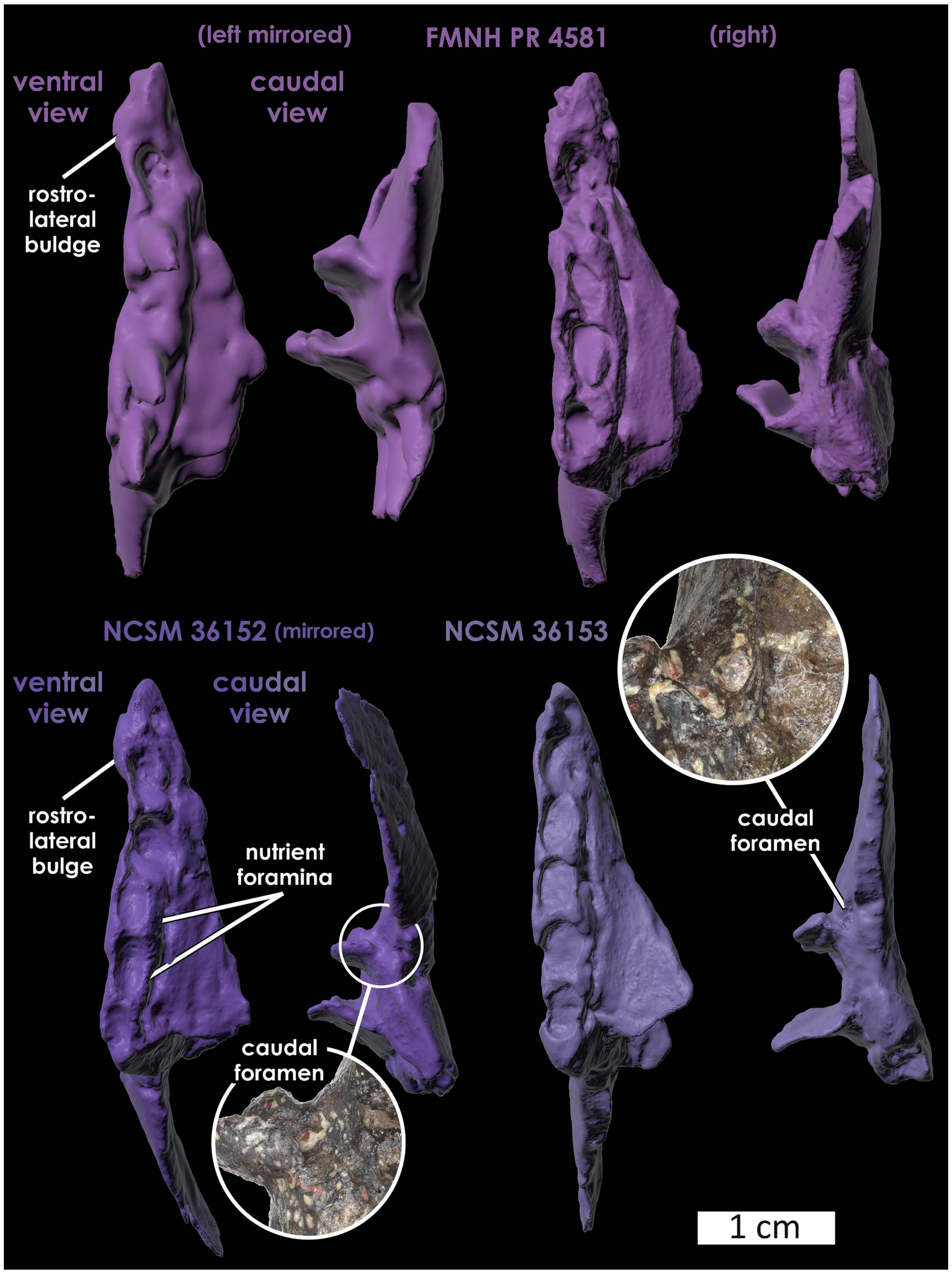

Fig 4. Premaxillae of *Fona herzogae* from multiple specimens in ventral and caudal views.

https://doi.org/10.1371/journal.pone.0353169.g004

Articulations: The premaxilla forms the rostral end of the snout as well as the caudal, ventral, and rostral margins of the external naris. The premaxilla connects with the maxilla caudally and the nasal caudodorsally. It likely contacted the vomer caudoventrally and bore a rhamphotheca rostrally [16,17]. All premaxillae show a complete lack of fusion, unlike in *Thescelosaurus neglectus* [18], *Changchunsaurus* [19], *Oryctodromeus* [20], and *Zephyrosaurus* [21], which are partially or fully fused.

Rostral surface: In lateral view, the rostrodorsal margin is angled caudodorsally (Fig 3). The rostral region of the premaxillae from the Mini Troll locality (NCSM 36152 and 36153) lacks a pronounced mediolaterally expanded shelf characterized by prominent rugosities, which is present in *Hypsilophodon* [7], *Jeholosaurus* [22], *Changchunsaurus* [19], *Th. neglectus* [18], *Zephyrosaurus* [21], and possibly to a lesser degree *Changmiania* [23] and *Oryctodromeus* [20]. On NCSM

36153, this region is weakly rugose and dominated by a dense array of small pits ~0.25 mm in diameter, a condition more similar to *Lesothosaurus* [24]. In *Haya,* the premaxilla is completely smooth and lacks rugosity or pitting altogether [25]. The premaxillae from the Manolo locality (FMNH PR 4581) also lack a distinct shelf; however, they are moderately rugose, suggesting this condition may be ontogenetically or intraspecifically variable. The rostral tip weakly overhangs the first tooth position as in *Haya* [25] and *Orodromeus* [26], although not to the extent as in *Th. neglectus* [18], *Changchunsaurus* [19], and *Hypsilophodon* [6]. Rostrodorsal to the first tooth position and immediately caudal to the premaxillary foramen, the lateral surface is slightly laterally swollen (Fig 4), as occurs in *Haya* [25] and *Orodromeus* (MOR 1141). This bulge is absent on NCSM 36153*, Th. neglectus,* and *Oryctodromeus* (MOR 1636). There is no rostral bone, which is a neomorphic element present in ceratopsians [27].

Lateral surface: The lateral surface of the premaxilla is not strongly dorsoventrally concave, as in *Th. neglectus* [18], and *Changchunsaurus* [19], but weakly flares laterally as in *Haya* [28]. At the caudal margin of the premaxilla, between the caudal process and the tooth row, there is a subtle semilunate fossa located along the premaxillary-maxillary boundary, herein termed the caudal premaxillary fossa. The fossa continues onto the maxilla and contributes to the rostral half of the anterior maxillary fossa, as in a number of other early diverging ornithischians such as *Th. neglectus* [18], *Changchunsaurus* [19], *Jeholosaurus* [22], *Orodromeus* [29], *Zephyrosaurus* [21], *Hypsilophodon* [7], *Isaberrysaura* [30], and the stegosaurian *Huayangosaurus* [31]. The caudal premaxillary fossa seems to be intraspecifically and contralaterally variable in *Fona*. It forms a prominent depression on the left and right of FMNH PR 4581, whereas, on the premaxillae from the Mini Troll locality, it is absent on NCSM 36153 (possibly due to poor preservation in the region) and less prominent on NCSM 36152.

Oral margin: Ventrally, the oral margin is not strongly laterally everted as in *Th. neglectus* [18]. Rostral to the first tooth position is an edentulous region no wider than a single tooth position. This is in contrast to *Th. neglectus* [18], in which this space spans the length of two tooth positions. A rostrolateral bulge is present near the snout, adjacent to the first tooth position. It is absent in NCSM 36153.

Dental count: There are only five premaxillary teeth (Fig 4), a condition shared by most early diverging ornithischians except for *Laquintasaura* [32] which possesses seven, *Th. neglectus* [18], *Jeholosaurus* [22], *Lesothosaurus* [3], *Isaberrysaura* [30], and *Scutellosaurus* [33], which possesses six teeth, the early diverging ceratopsian *Beg tse* [34] and the early diverging ornithopod *Convolosaurus* [35], which possess four teeth, heterodontosaurids, which possess three teeth [36], and the Elasmarian *Talenkauen* [37]*,* which possesses two teeth. Detailed descriptions of the premaxillary, maxillary, and dentary dentitions are presented at the end of the osteology section, following the descriptions of all cranial elements.

Dorsal and caudal processes: The rostral dorsal process is entirely or partially missing on all premaxillae. The caudal process arises at the caudodorsal corner of the premaxilla. It projects steeply caudodorsally, and in medial view, a subtle rostrocaudally oriented ridge can be seen, which likely abutted a facet on the lateral surface of the nasal. The process likely did not contact the lacrimal, as opposed to the condition in heterodontosaurids [36,38], the neornithischian *Jeholosaurus* [22], early diverging ceratopsians [34], and some early diverging iguanodontians [39] (see lacrimal section for an elaboration on this feature.) The lateral surface of NCSM 36152 bears a tuberosity on the caudodorsal process, which is absent in NCSM 36135 and FMNH PR 4581.

Palatal surface: In medial view, there are two parallel, rostrocaudally oriented, short, thin projections. The ventral projection forms the palatal surface. The vomer likely overlaps this projection caudally, as in *Th. neglectus* [18], however the lack of a vomer prohibits confirmation of this configuration. The dorsal projection is shorter than the ventral. Together, these two projections form a slot for the rostral process of the maxilla.

Foramina: In lateral view, there are three prominent foramina located near the rostral margin. The caudodorsal-most foramen opens into a caudodorsally directed canal. This foramen likely corresponds with the premaxillary foramen reported in *Th. neglectus* [18], *Changchunsaurus* [19], *Haya* [28], *Oryctodromeus* [20], *Zephyrosaurus* [21], *Jeholosaurus* [22], *Lesothosaurus* [3], the elasmarian *Talenkauen* [37], and the early diverging iguanodontians *Zalmoxes* [40] and *Te. tilletti* (OMNH 58340).

There is a second, unnamed foramen located rostroventrally to the premaxillary foramen, which is oriented mediolaterally. Continuing rostroventrally toward the tip of the premaxilla, there is a third, rostrally directed foramen that likely represents the rostral premaxillary foramen [sensu 24]. The rostral premaxillary foramen likely connects with the rostral palatal foramen, as in *Lesothosaurus* [3], *Th. neglectus* [18], and likely *Changchunsaurus* [19]. It can be seen in medial view, located directly rostral to the first premaxillary tooth position (Fig 3).

In caudal view, a foramen, herein termed the caudal premaxillary foramen, is located at the junction between the base of the caudal process and the medially directed dorsal projection (Fig 4). It is present in both complete premaxillae from the Mini Troll locality (NCSM 36152 and 36153), but is less obvious on NCSM 36152. It likely represents an opening for the trigeminal nerve V of the ophthalmic branch [41]. It is not reported in any other early diverging ornithischian and is not observed in the fused premaxilla of *Oryctodromeus* (MOR 1636). In medial view, there is a row of rostrocaudally elongate, slit-like nutrient foramina located directly ventral to, and running parallel with, the tooth row.

Maxilla

Preservation: Three maxillae are represented from the Mini Troll locality (Fig 5). NCSM 36143 is a right maxilla and the best preserved of the three. It preserves the majority of the rostrocaudal length and is only missing portions of its caudal end. Additionally, it has a slight deformation across the lateral surface near the maxillary fenestra. NCSM 36141 likely represents the contralateral left to NCSM 36143, as it has a similar mediolateral width, an equivalent distance between tooth positions, and appears to have a similar placement of some foramina across the lateral face. It preserves the caudal two-thirds of the maxilla and is missing portions of its caudal and rostral ends. NCSM 36142 preserves the rostral portion of a left maxilla and lacks erupted teeth in each alveolus. It cannot be the rostral half of NCSM 36141, as the two elements have overlapping regions. Additionally, when viewed ventrally, the distance between tooth positions appears slightly shorter than NCSM 36143, suggesting it may belong to a slightly smaller individual.

General shape: The maxilla is a rostrocaudally elongate bone characterized by a tooth-bearing ventral margin and a caudodorsally projecting ascending process that emerges near the rostral end of the element. It is "V" shaped in cross section, with a deep, rostrocaudally oriented trough that runs across the length of the element, forming the ventral margins of the antorbital fossa.

Articulations: It connects with the premaxilla rostrally and also likely connects with the jugal and lacrimal caudolaterally, which would have excluded it from the orbital margin, the palatine medially, the nasal dorsally, and the ectopterygoid caudomedially, as in *Haya* [25], however these connections are not preserved. Dieudonné et al. [42; Supplemental File 4] incorrectly interpreted the skull reconstruction presented in Avrahami et al. [9] as showing true maxillary participation in the orbital margin, when in fact, the maxilla was "exposed" along the orbital margin as a result of a damage to the ventral aspect of the lacrimal where it would have contacted the jugal. The preserved lateral and dorsal margins of the maxilla indicate that the jugal and lacrimal would likely have contacted along the orbital rim, consistent with the condition in other early diverging ornithischians. Based on their interpretive error, Dieudonné et al. [42; Supplemental File 4] note that they performed a counter-clockwise rotation of the postorbital, parietal, and squamosal, combined with downward displacement of the quadrate, and rescored the skull based on this alternative reconstruction. In the absence of an image of this alternative hypothesis, we cannot evaluate this alternative reconstruction; however, we note that it is correcting an "error" that does not exist.

Lateral view: The lateral surface of the caudal portion of the maxilla is marked by a pronounced, rostrocaudally oriented buccal ridge, causing the tooth row to become medially inset, as in most early diverging ornithischians. This ridge appears to be straight, as in *Haya* [25], and in contrast to the ventrally bowed buccal ridge in *Th. neglectus* [18]. The buccal ridge becomes weakly defined rostrally on NCSM 36143.

Ascending process: The ascending process emerges rostrally from the lateral surface and projects caudodorsally. The process is mediolaterally thin with dorsal and ventral margins that run parallel to each other.

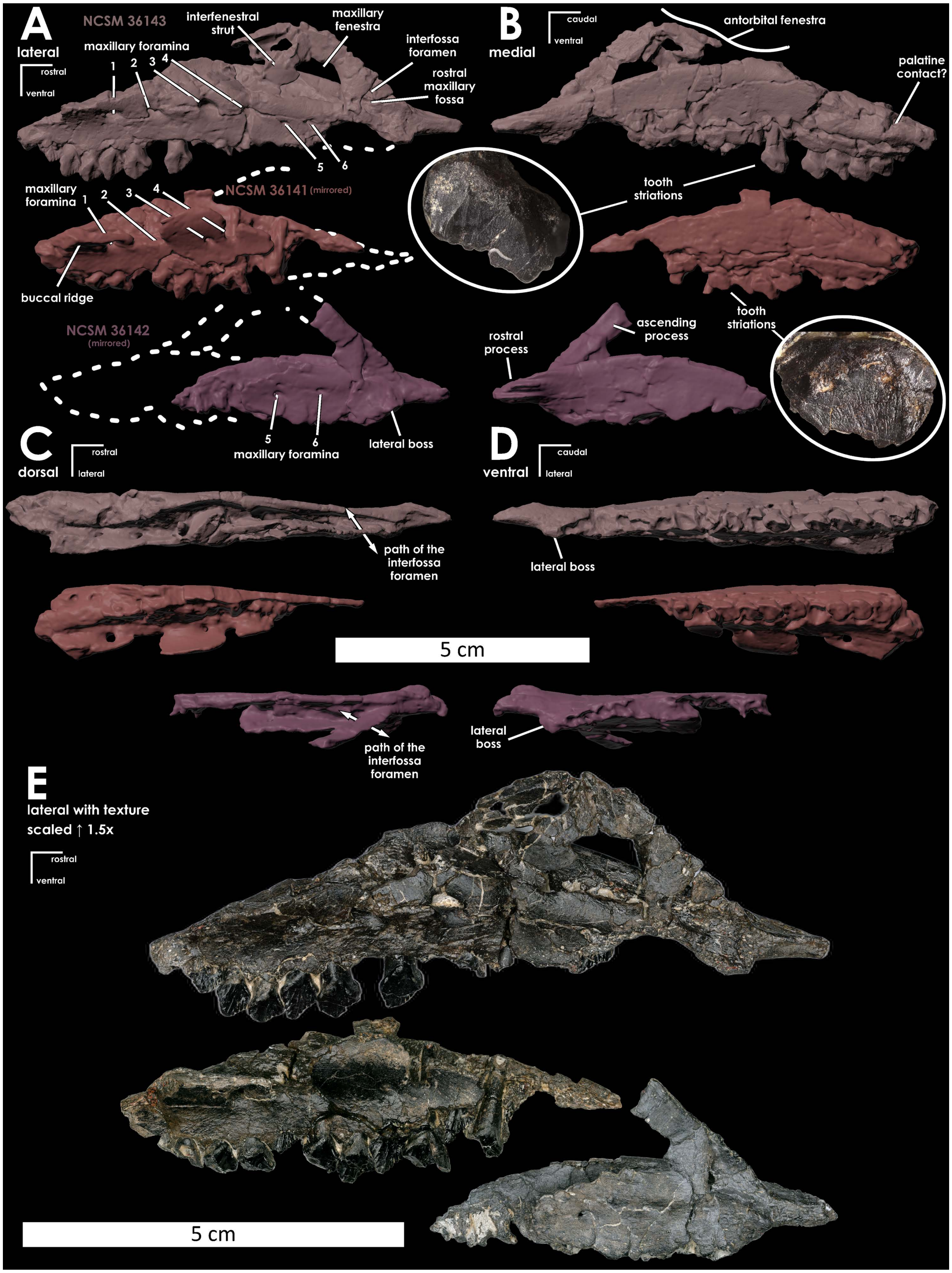

Fig 5. Maxillae of *Fona herzogae*. Dashed lines represent missing bone.

https://doi.org/10.1371/journal.pone.0353169.g005

Rostral maxillary fossa: The base of this process is marked by a semilunate fossa called the anterior maxillary fossa, which continues onto the caudal margin of the premaxilla. This fossa is present in *Th. neglectus* [18]*, Changchunsaurus* [19]*, Haya* [25]*, Hypsilophodon* [7]*, Jeholosaurus* [22]*, Orodromeus* [29]*, Zephyrosaurus* [21], *Changmiania* [23], *Galleonosaurus* [43], and possibly *Isaberrysaura* [30]. The caudal margin of the anterior maxillary fossa occurs caudal to the first tooth position, whereas in all other early diverging ornithischians that have the fossa, except for *Isaberrysaura* [30], it occurs rostral to the first tooth position.

In *Fona,* there is a rostrocaudally oriented interfossa foramen located at the caudal margin of the rostral maxillary fossa that pierces through the ascending process into the maxillary fenestra. The foramen is much larger on NCSM 36142.

Diastema: Ventral to the anterior maxillary fossa, there is a small diastema present between the first maxillary tooth position and the last premaxillary tooth position. This diastema is short and only spans a single tooth position.

Rostral process: The rostral-most part of the maxilla is characterized by a prominent spike-like process that fits in between the medial projections of the premaxilla and is also present in *Th. neglectus* [18] and *Orodromeus* [29].

Rostrolateral boss: At the caudoventral margin of the rostral process, there is a small anterolateral boss. This boss is present in *Changchunsaurus, Haya, Orodromeus, Oryctodromeus, Zephyrosaurus,* and *Th. neglectus* [18].

Rostral portion: In dorsoventral view, the maxilla becomes mediolaterally thinner towards the rostral end, a condition shared by *Haya* [25]. The element is rostrocaudally straight and lacks the caudolateral kink present in *Galleonosaurus* and *Camptosaurus* [43].

Foramina: On NCSM 36143, there are at least six nutrient foramina located ventrally and running parallel to the buccal ridge. The rostral two are located rostral to the interfenestral strut. They are more closely spaced and much smaller than the caudal four. The caudal four foramina are much larger and spaced an equal distance from each other. In contrast to *Th. neglectus* [18] there is no row of smaller foramina across the lateral surface of the buccal ridge.

Maxillary fenestra: NCSM 36143 seems to preserve a maxillary fenestra located rostral to the antorbital fenestra. This maxillary fenestra is also present in *Haya* [25] and *Hypsilophodon* [7]. The caudal margin of the maxillary fenestra is formed by a mediolaterally thin interfenestral strut, which emerges from the dorsolateral surface of the maxilla, slightly rostral to the maxilla's mid-length, and projects rostrodorsally, merging dorsally with the ascending dorsal process to form a 'U' shaped arc. However, the interfenestral strut is significantly damaged, and its exact morphology is difficult to determine.

Similar accessory fenestrae located within, rostral to, or dividing the antorbital fenestra are present in *Kulindadromaeus* [44], specimens of *Lesothosaurus* (MNHN LES 17) [45], and possibly specimens of *Orodromeus* (MOR 249); however, the homology of these structures remains unclear.

Medial view: In medial view, there is a row of rostrocaudally elongate, slit-like special foramina located directly ventral to, and running parallel with, the tooth row (see dentary description for more detail on the special foramina). The medial surface of the maxilla is flat, in contrast to the dorsoventral concavity in *Th. neglectus* [18]. Caudally, the medial surface is covered in a series of fine, rostrocaudally oriented ridges, as in *Changchunsaurus* [19] and *Zephyrosaurus* [21].

Tooth row: In ventral view, the tooth row is straight and does not curve medially or laterally. On NCSM 36143, tooth positions 1–18 can be seen, whereas on NCSM 36142, positions 1–10 or 11 can be seen. On NCSM 36141, ten tooth positions can be verified, which likely correspond to tooth positions 17–8 (± one tooth position). All teeth are missing from NCSM 36142. NCSM 36143 contains five fully erupted, well-preserved teeth (11, 13–16). Teeth 8–10 appear to have been partially or fully erupted; however, they are broken in transverse cross-section. Tooth positions 5, 10, and 17 contain partially erupted replacement teeth. All other alveoli are empty. On NCSM 36141, all tooth positions house fully erupted teeth except for 17, 13, and 8, in which the former is empty and the latter two contain partially erupted replacement teeth. The presence of eighteen or more maxillary teeth is a condition more closely shared by the early diverging ornithischians *Hexinlusaurus, Th. neglectus, Kulindadromaeus,* and *Isaberrysaura* [18,30,44,46], as opposed to other neornithischians

that usually display 13–15 maxillary teeth [25]. Detailed descriptions of the premaxillary, maxillary, and dentary dentitions are presented at the end of the osteology section, following the descriptions of all cranial elements.

Nasal

Preservation: A single partially preserved right nasal was recovered from the Mini Troll locality (NCSM 36144) (Fig 6).

General shape: The nasal is a rostrocaudally elongate sheet-like bone characterized by a flared caudal end.

Articulations: The nasal forms the dorsal margin of the external naris and connects with the premaxilla rostrolaterally, the prefrontal caudolaterally, and the frontal caudomedially. The connections with the lacrimal and maxilla remain uncertain, as these elements are poorly preserved and partially incomplete around the area where articulation with the nasal would occur.

Lateral surface: The lateral surface of the nasal is marked by a distinct rostrocaudally oriented, slightly dorsoventrally concave facet. This facet begins rostral to the lateral process as a dorsoventrally short groove that broadens rostrally

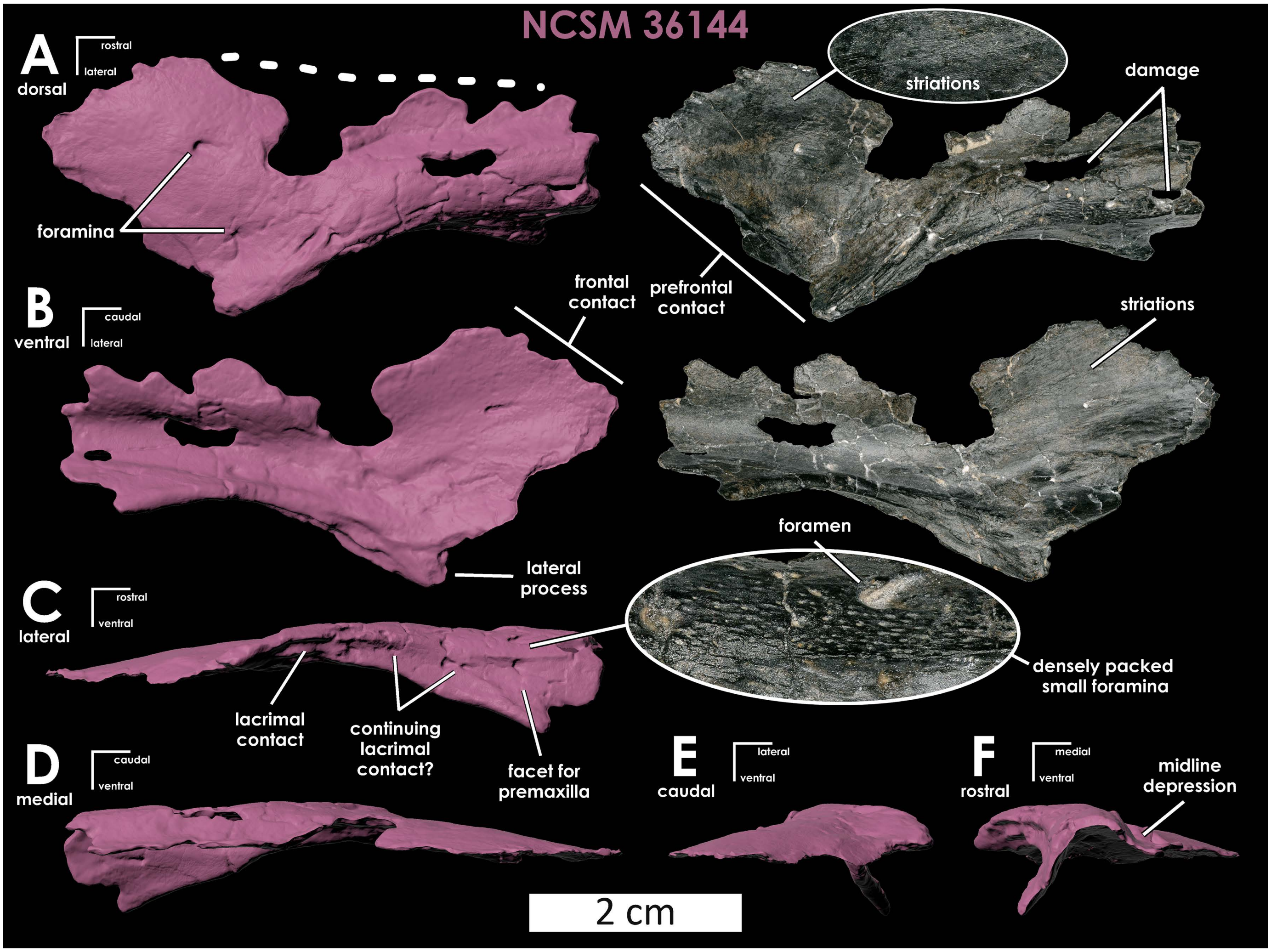

Fig 6. Nasal of *Fona herzogae*. Dashed lines represent missing bone.

https://doi.org/10.1371/journal.pone.0353169.g006

(Fig 6C). The caudal two-thirds of this facet likely formed a junction with the dorsal portion of the maxilla or possibly the lacrimal, although these connections are not preserved. The rostral third of the facet forms a slightly deeper, triangular facet that accepts the caudal process of the premaxilla. This configuration is common among most early diverging ornithischians except *Th. neglectus* [18] in which the nasal overlaps the dorsal process of the maxilla. In *Dryosaurus, Dysalotosaurus, Kulindadromeus, Changmiania, Jeholosaurus*, possibly *Convolosaurus*, and heterodontosaurs, the caudal process of the premaxilla contacts the lacrimal across this surface, with the extent or contribution of each element varying between taxa [22,23,35,36,38,44,47].

Dorsal view: The dorsal surface of the nasal is rostrocaudally flat, slightly mediolaterally convex along its caudal half, and strongly mediolaterally convex along its rostral half. The nasals are unfused along the internasal suture, as in *Haya* [25]. The rostromedial portion of the dorsal surface is slightly depressed, indicating the presence of the midline depression (Fig 6F). This depression is present in the early diverging ornithischians *Hexinlusaurus*, *Jeholosaurus, Haya, Kulindadromeus, Changchunsaurus, Heterodontosaurus*, *Agilisaurus, Isaberrysaura*, and *Changmiania* [19,22,23,25,30,36,44,46,48], the early diverging ceratopsians *Yinlong* [49] and *Liaoceratops* [50], and the early diverging stegosaur *Huayangosaurus* [31]. This depression is also present in *Th. neglectus* [contra 18], although it is not as prominent.

Rostral portion: In contrast to *Haya* [25] the nasals do not arch dorsally near the rostral end; however, this may be due to incomplete preservation.

Caudal portion: In dorsal view, the caudal portion of the nasal forms a dorsoventrally thin, triangular sheet of bone that is overlapped across its caudolateral margin by the rostral portion of the prefrontal (Fig 6A). The caudal margin of the nasal forms a caudomedially oriented contact with the rostral ends of the frontals. Near the margins of these contacts, the dorsal surface shows slight rostrocaudally oriented striations. More prominent rugose striations are also present dorsal to the lateral process and lateral facet.

Foramina: A single foramen is located near the mediolaterally widest portion of the nasal, as in *Haya* [25]. This is in contrast to the condition in *Jeholosaurus* [22] and *Th. neglectus* [18], in which a row or series of foramina are located more laterally. Several smaller foramina are present dorsal to the premaxillary facet, across a region that is moderately rugose; however, these structures do not pierce through the element.

Jugal

Preservation: In total, five jugals are represented for *Fona*, a left and right NCSM 33548 from the Karmic Locality, whereas the Mini Troll locality is represented by two lefts and a right (Fig 7).

The right jugal of NCSM 33548 is well preserved rostrally but missing portions of its caudal and dorsal sections, whereas the left is less preserved overall. A right (NCSM 36138) and a left (NCSM 36140) bear well-preserved caudal portions but are missing the rostral-most portions of their rostral processes. NCSM 36139 is well preserved overall, but it is missing the caudoventral portion that contacts the ventral edge of the quadratojugal.

General shape: The jugal is a mediolaterally flattened strap-like bone characterized by a central body with four processes that project rostrally, dorsally, caudally, and caudoventrally. The jugal appears somewhat like a right-angled triangle, longer than it is tall, with a hypotenuse that forms an inward arc. The lateral surface of the jugal is mildly convex, whereas the medial surface is concave.

Articulations: The jugal forms the rostroventral margin of the infratemporal fenestra and the caudoventral margin of the orbit. It connects to the postorbital caudodorsally, the quadratojugal caudally, the ectopterygoid medially, the maxilla rostromedially, and the lacrimal rostrally.

Ornamentation: The lateral surface of the main body of the jugal is either featureless and flat, as in *Th. neglectus* [18], or may bear a subtle tuberosity with moderate rugosity, as in *Jeholosaurus* [22]. However, no jugal bears the distinct

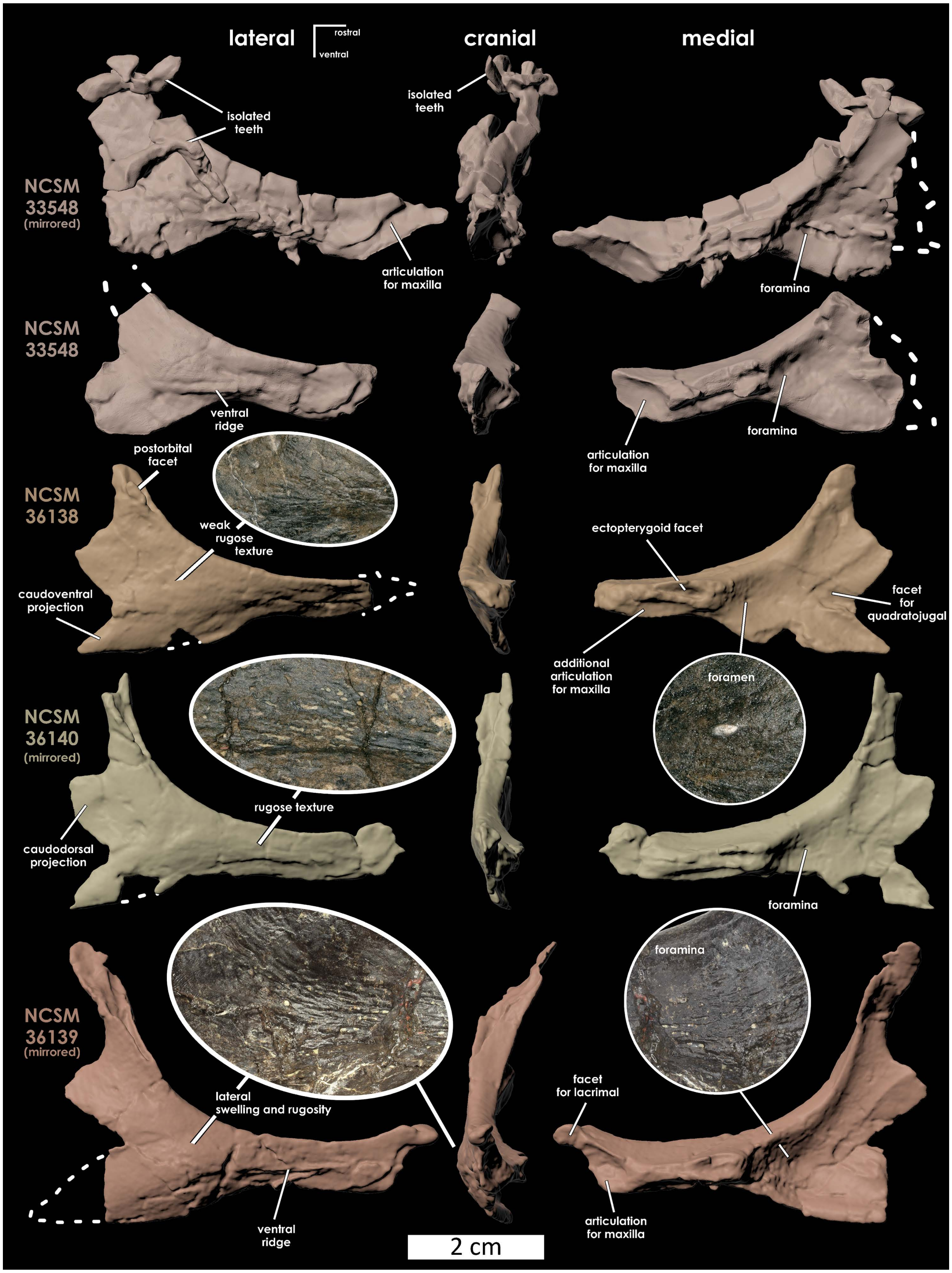

Fig 7. Multiple jugals of *Fona herzogae*. Dashed lines represent missing bone.

https://doi.org/10.1371/journal.pone.0353169.g007

caudolaterally directed jugal horn that is present in *Orodromeus* [29], *Zephyrosaurus* [21,51], and some heterodontosaurids [36,52–54]. See discussion for an elaboration on this feature.

Rostral process: The longest process of the jugal is the rostral process (=maxillary process), which makes up the rostral half of the jugal as it projects rostrally from the center of the jugal. The dorsal surface of the rostral process, which forms part of the orbital margin, is tilted at a ventromedial angle. In lateral view, the contact with the lacrimal likely sits at the tip of a rostrodorsally inclined projection. This projection would have overhung the maxilla at the tip of the rostral process, as in *Hypsilophodon,* but is not as dorsally inflected as *Agilisaurus* [48].

In medial view, the rostral process is characterized by a medially expanded shelf. This expanded region houses a deep rostrocaudally oriented groove on its caudodorsal margin that forms the articulation surface for the ectopterygoid. The contact with the maxilla occurs ventral and dorsal to this expansion. The dorsal contact is a lateral concavity that starts shallow and narrow directly rostral to the expansion, broadening and deepening rostrally, whereas the ventral contact occurs directly ventral to the expansion as a rostrocaudally elongate concavity. This condition is shared by *Th. neglectus* [18], *Zephyrosaurus* [21,55], and possibly *Oryctodromeus,* but differs from some early diverging iguanodontians in which the jugal inserts into the maxilla, e.g., *Te. tilletti* [4] and *Iani* [1]. It is also dissimilar from the condition in *Haya* [25], *Orodromeus* [26], and *Heterodontosaurus* [52] in which the rostral process is bifid. *Zephyrosaurus* (MCZ 4392) may also show a bifid condition, however the rostral end of the maxillary processes is incomplete.

Quadratojugal process: The caudal aspect of the jugal is characterized by a dorsoventrally broad, sheet-like structure that is bifurcated into two separate, mediolaterally flattened projections, termed the caudodorsal projection and the caudoventral projection. The medial surface of the caudodorsal projection is the thinnest area of the jugal, forming a slightly depressed facet for the quadratojugal. The caudodorsal projection is absent in *Changmiania* [23], *Oryctodromeus* [20], and *Zephyrosaurus* [21], although its absence may be an artifact of taphonomy, as only three of the five jugals of *Fona* preserve this feature. A caudodorsal projection is present in *Orodromeus* [29; Ch. 3 Figure 3 B], *Jeholosaurus* [22], *Haya* [28], *Th. neglectus* [18], and possibly *Changchunsaurus* [19]. In these taxa, the projection is slightly smaller, relative to the jugal, and projects caudodorsally, whereas when preserved in *Fona,* the base of the projection is dorsoventrally broader. In both *Fona* and *Th. neglectus,* the shape and orientation of this projection vary across individuals and/or contralateral pairs. A dorsal projection of the caudal process is also present on an isolated ornithopod jugal from the Albian-aged Griman Creek Formation of Southeast Australia [55]. The caudoventral projection is located directly ventral to the caudodorsal projection and is directed caudoventrally, tapering to a sharp point. The medial surface is concave dorsoventrally, creating a scoop shape that follows the path of the projection caudoventrally. It slots into a groove on the ventromedial margin of the quadratojugal (Fig 8 C), which is similar to the condition in *Th. neglectus* [18], *Te. tilletti* [4], *Orodromeus* [29; Ch. 3 Figure 3 B], and *Zalmoxes* [40], and in contrast to the laterally overlapping condition in *Haya* [25].

Postorbital process: The dorsal (=postorbital) process is caudodorsally inclined and has a dorsal half that forms a mediolaterally thin sheet of bone. The ventral half becomes thicker to form a triangular cross-section that points caudally, as in *Th. neglectus, Oryctodromeus,* and *Zephyrosaurus.* The rostrolateral margin is characterized by a tall, triangular slot that accepts the ventral process of the postorbital.

Ventral margin: The ventral margin maintains a mediolaterally compressed edge across the length of the jugal. In lateral view, the ventral margin of the jugal follows a straight path from the ventral projection of the caudal process, stepping dorsally as it transitions toward the rostral process, a condition shared by *Th. neglectus* [18] and *Haya* [25]. The obliquely inclined ridges present on the ventrolateral surface of the jugal in *Th. neglectus* are not present [18].

Medial surface: In medial view, there is a foramen located within the medial concavity, as in *Lesothosaurus* [3]. It is located within the deepest part of the concavity, directly opposite the lateral bulge. The medial concavity is more pronounced on jugals with the lateral surface rugose swelling.

Quadratojugal

Preservation: Two quadratojugals are represented from the Mini Troll locality. NCSM 36160 (Fig 8) is well preserved whereas NCSM 36161 (S1.05 File) is poorly preserved.

General shape: The quadratojugal is a mediolaterally flattened, trapezoidal, plate-like bone characterized by an undulating body and a small foramen (Fig 8).

Articulations: The quadratojugal forms the caudoventral margin of the infratemporal fenestra and connects to the jugal rostrally, the quadrate caudomedially, and possibly the squamosal dorsally.

Lateral surface: The lateral surface of the quadratojugal is slightly concave, as in *Th. neglectus* and *Haya* [25], and in contrast to *Te. tilletti* [4], and *Jeholosaurus* [22], in which it is gently convex. In lateral view, the rostral portion is marked by a caudodorsally inclined, rectangular facet, which accepts the caudodorsal projection of the jugal.

Contact with Jugal: The quadratojugal has a complex connection with the jugal that was previously considered a possible autapomorphy of *Th. neglectus* [18]. In all other neornithischians, no portion of the jugal contacts the medial surface of the quadratojugal. However, in *Th. neglectus* [18] and *Fona,* the ventral projection of the caudal process of the jugal,

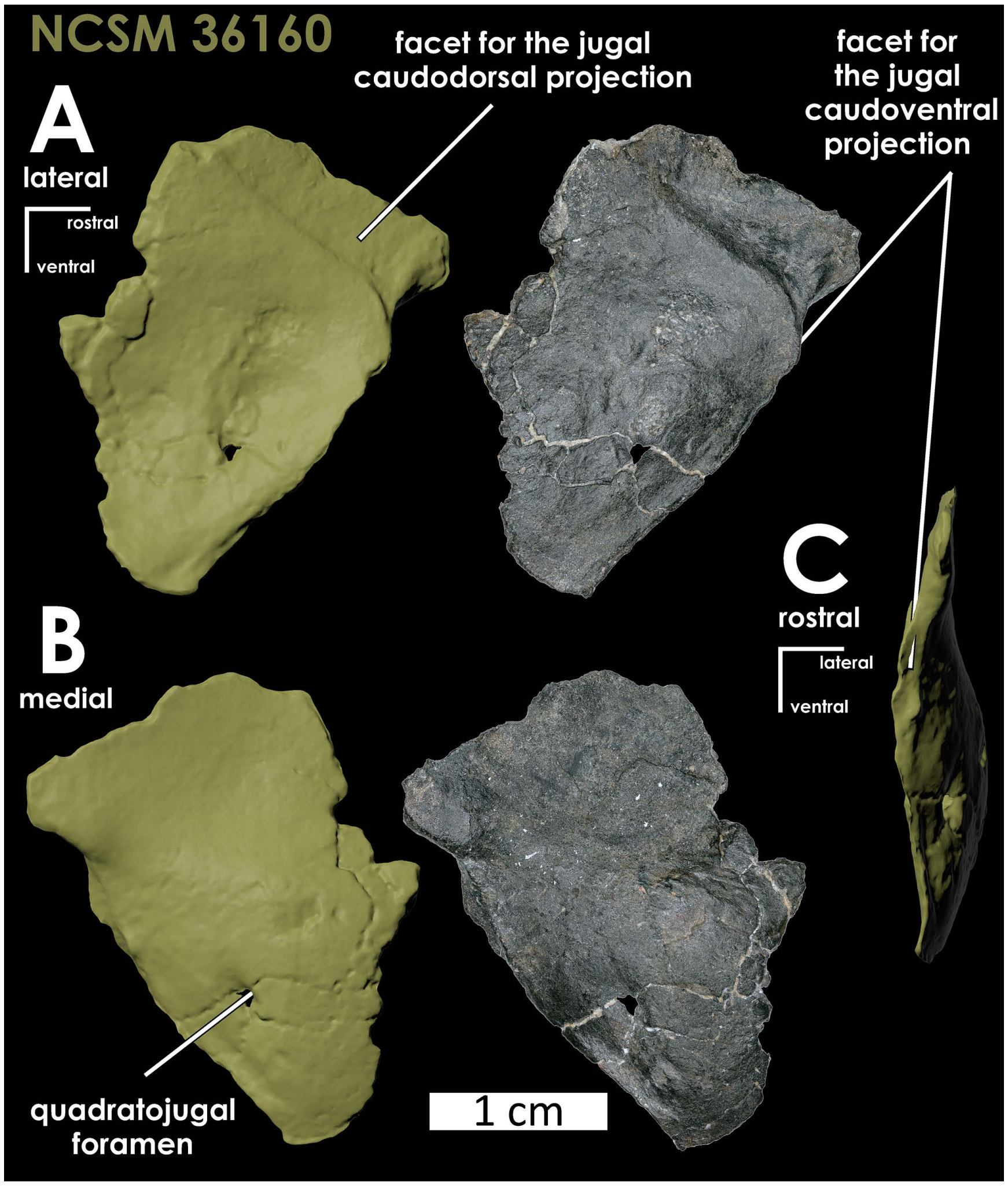

Fig 8. Quadratojugal of *Fona herzogae*.

https://doi.org/10.1371/journal.pone.0353169.g008

contacts the ventromedial surface of the quadratojugal (Fig 8C). More specifically, the dorsal margin of the projection slots into a small groove located along the ventral edge of the quadratojugal. This is dissimilar to the condition present in *Zalmoxes,* in which the jugal contains a recess for the rostral margin of the quadratojugal [40].

Contact with quadrate: The caudomedial portion of the quadratojugal is concave and oppresses against a concave surface along the ventral half of the quadrate. There is no sign of a paraquadratic foramen.

Foramen: The quadratojugal houses a single foramen that pierces directly through the element mediolaterally. This foramen is widely distributed across Neornithischia. However, its shape, size, and specific location often differ. In *Jeholosaurus, Hypsilophodon,* and some specimens of *Haya,* the foramen is a wide, circular perforation [7,22,25]. In other specimens of *Haya,* the foramen is slightly smaller and ovate [25]. Similar variability is present in *Orodromeus,* with the foramen being relatively larger and more open in ontogenetically older specimens (MOR 1141) and smaller in less mature individuals (MOR 294) [26]. In *Fona*, *Th. neglectus* and *Gasparinisaura,* the foramen is much smaller [18,56]. The placement of this foramen in *Fona* also differs from *Th. neglectus* and is located further ventrally and closer to the quadrate.

Postorbital

Preservation: Four postorbitals of *Fona* are preserved: A well-preserved, complete, partially deformed left from the Mini Troll locality (NCSM 36149), a partially preserved right from the Mini Troll locality (NCSM 39062) and a well-preserved contralateral pair from NCSM 33548.

General shape: The postorbital is a triradiate bone shaped characterized by three processes (Fig 9).

Articulations: The postorbital forms the rostrodorsal margin of the infratemporal fenestra and the caudodorsal margin of the orbit. It connects to the jugal ventrally, the squamosal caudally, the parietal dorsomedially, the laterosphenoid medially, and the frontal rostrodorsally.

Ventral process: The ventral process (= jugal process) of the postorbital has a triangular cross-section and tapers to a point that fits into a slot on the dorsal process of the jugal.

Caudal process: The caudal process (= squamosal process) of the postorbital is a mediolaterally thin projection that articulates with the rostral process of the squamosal. In lateral view, the dorsal and ventral margins of the process converge at the tip and do not show the bifid condition present in *Th. neglectus* and possibly some specimens of *Haya* [18,25]. The lateral surface of the caudal process is dorsoventrally convex, a condition shared by *Haya*, *Orodromeus*, *Oryctodromeus*, and possibly *Changmiania* [23]. This is in contrast to the condition in *Th. neglectus* [18] in which the lateral surface of the process is dorsoventrally concave, a condition possibly shared by *Changchunsaurus* [19,57]. The medial surface of the process of NCSM 36149 and 39062 is marked by a rostrocaudally elongated shallow groove, which serves as a slot for the rostral process of the squamosal, as in some hadrosaurids [58], whereas NCSM 33548 possesses a pronounced ridge in the same area.

Rostral process: The rostral process is a rostrocaudally expanded straplike process that bears complex sutural facets with the frontal and parietal medially, forming a "T" shaped junction between the three elements.

Socket for the laterosphenoid: In medial view, part of the socket for the laterosphenoid is located at the caudodorsal corner of the rostral process, caudal to the orbital margin, and lateral to the parietal contact. This socket continues laterally onto the caudomedial corner of the frontal, a condition shared by *Hypsilophodon* [59]*, Th. neglectus* [18], *Thescelosaurus assiniboiensis* [60], *Oryctodromeus* [20], and an isolated orodromine frontal TMP 2003.012.0135 (possibly *Albertadromeus*) [61: fig. 9A]. In contrast, the laterosphenoid socket is restricted to only the postorbital in *Dryosaurus* [62], whereas *Haya* seems to lack this connection completely [25]. The medial surface of the concave socket is rugose and consists of a series of small excavation pits and dimples.

Lateral surface: The lateral surface of NCSM 36149 is mostly smooth and lacks the prominent nodular ornamentation present in *Jeholosaurus* [22], pachycephalosaurs [63], and some early diverging ceratopsians [49,64]. However, the surface of the rostral protuberance on the lateral surface of the orbital rim of NCSM 33548 bears prominent rugosities, which

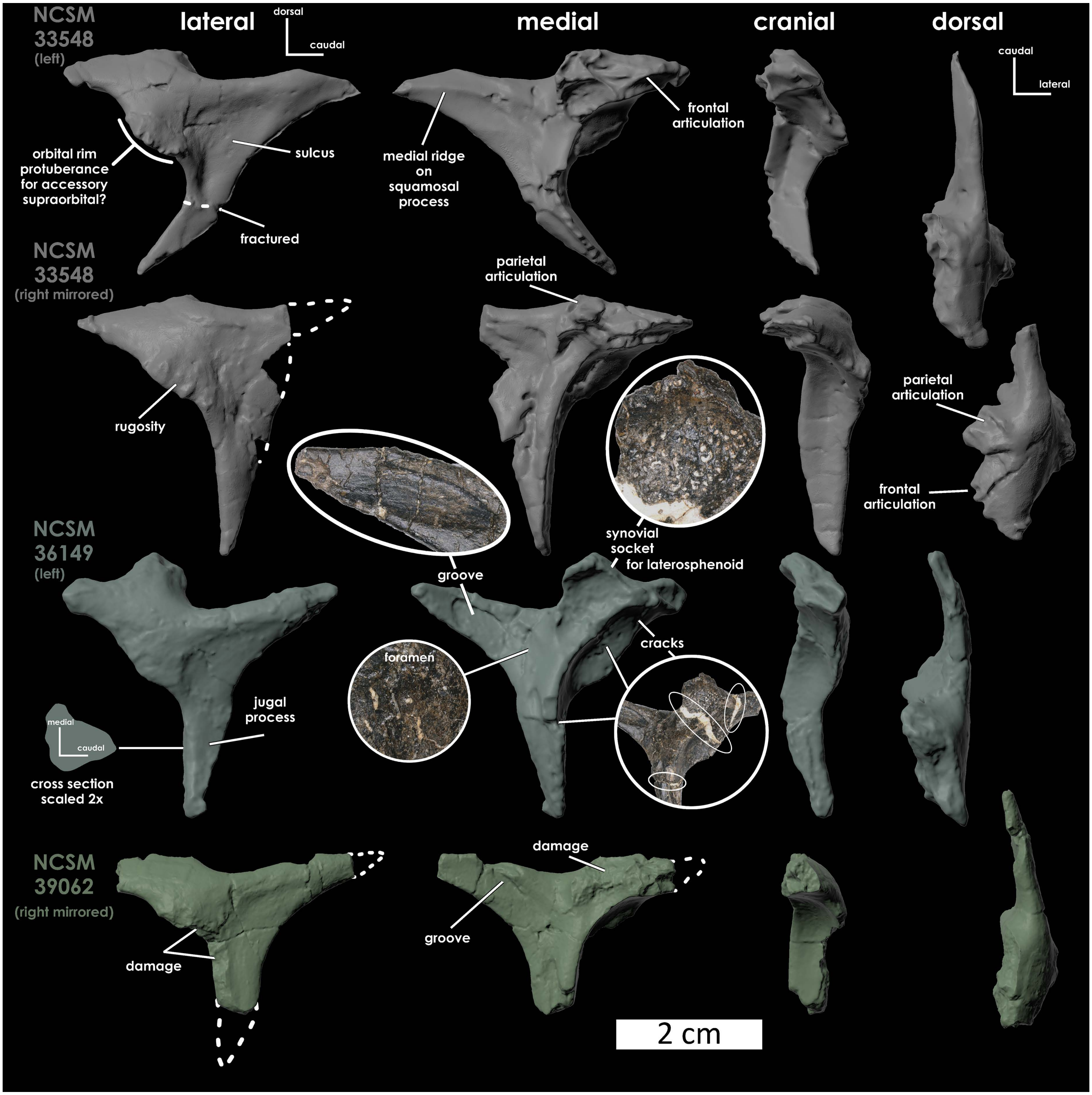

Fig 9. Multiple postorbitals of *Fona herzogae*. Dashed lines represent missing bone.

https://doi.org/10.1371/journal.pone.0353169.g009

possibly served as the contact for a posterior supraorbital [39]. A similar protuberance is present on *Th. neglectus* [18], *Haya* [25], *Orodromeus* [29], *Oryctodromeus* [20], *Zephyrosaurus* [21], *Jeholosaurus* [22], *Hexinlusaurus* [46], *Dryosaurus* [65], and possibly *Changchunsaurus* [19], but seems to be absent in *Changmiania* [23]. There is a shallow sulcus that runs across the caudal portion of the lateral surface. This sulcus is present in *Yandusaurus* [66], *Th. neglectus* [18], and is variably present in some specimens of *Haya* [25], suggesting that it may be ontogenetically variable. It also appears to be extensive in *Hexinlusaurus* [46]. In contrast to *Th. neglectus* [18], there are no foramina present across the lateral surface.

Medial surface and orbital margin: The medial surface of the postorbital is characterized by a prominent curved ridge that runs from the rostral process to the ventral process. Rostral to this ridge, the medial surface is concave, forming the postorbital contribution to the orbit. A single medial foramen occupies the region caudal to the orbital rim ridge at the base of the squamosal process.

Quadrate

Preservation: A well-preserved, complete right quadrate (NCSM 36159) and two poorly preserved, partially complete right quadrates (FMNH PR 4581 and NCSM 33548) are referred to *Fona* (Fig 10).

General shape: The quadrate is a dorsoventrally elongate columnar element that is characterized by two disproportionately sized bony plates, which project rostrally from the main shaft and thin distally, forming a sharp ridge in caudal view and a deep groove in rostral view.

Articulations: The quadrate forms the majority of the caudal margin of the infratemporal fenestra and articulates with the squamosal dorsally, the quadratojugal rostrally, the pterygoid rostromedially, and the surangular ventrally.

Lateral surface: In lateral view, the dorsal third of the quadrate is caudally inflected such that the dorsal articulation with the squamosal is located caudal to the plane of the quadrate ventral condyles, a condition that is shared by most early diverging ornithischians. In caudal view, the shaft of the quadrate remains straight and lacks mediolateral curvature.

Quadratojugal wing: The smaller lateral plate is the quadratojugal wing (= jugal wing), which emerges at the lateral margin of the dorsal condyle and flares slightly rostrally as it descends ventrally. At approximately two-thirds of the total height of the quadrate, the quadratojugal wing forms a concave contact to accept the quadratojugal. This depression extends ventrally, terminating just above the articular surface of the ventrolateral condyle.

Pterygoid wing: The larger medial plate is the pterygoid wing, which emerges a short distance below the head of the dorsal condyle and gradually flares rostromedially as it descends ventrally. It reaches a maximum extent near the middle of the quadrate's dorsoventral height. Ventral to this point, the wing sharply attenuates, terminating dorsal to the medial ventral condyle. A mediolaterally expanded buttress-like structure occupies the point halfway between the apex of the wing and the shaft, possibly to serve as an accessory articulation point for the pterygoid, similar to the pterygoid wing ventral groove in *Th. neglectus* [18]

Foramen: The paraquadratic foramen is present and occurs on the lateral surface along the quadrate-quadratojugal contact. It forms a closely appressed slit in contrast to the open foramen in *Th. neglectus* [18] and some early diverging iguanodontians and ankylopollexians [39]. On NCSM 36159, there is a dimple, only visible in rostral view, located towards the ventral third of the quadrate, within the mediolateral concave groove that runs dorsoventrally between the two quadrate wings, which possibly represents the exit of the quadrate foramen. It appears to be closed and is not present on NCSM 36173, NCSM 33548, or FMNH PR 4581. A second dimple occurs along the lateral surface of the quadratojugal wing, slightly caudodorsal to the maximum extent of the quadratojugal wing, but is likely due to breakage.

Fossa: A dorsoventrally elongate fossa occupies the majority of the pterygoid wing medial surface. It is subtle, with less pronounced margins than those of *Oryctodromeus* and *Th. neglectus.*

Ventral condyles: The ventral-most aspect of the quadrate is defined by two transversely oriented, equally sized ventral condyles spaced an equal distance from each other along the midline of the shaft. In contrast, *Orodromeus* and

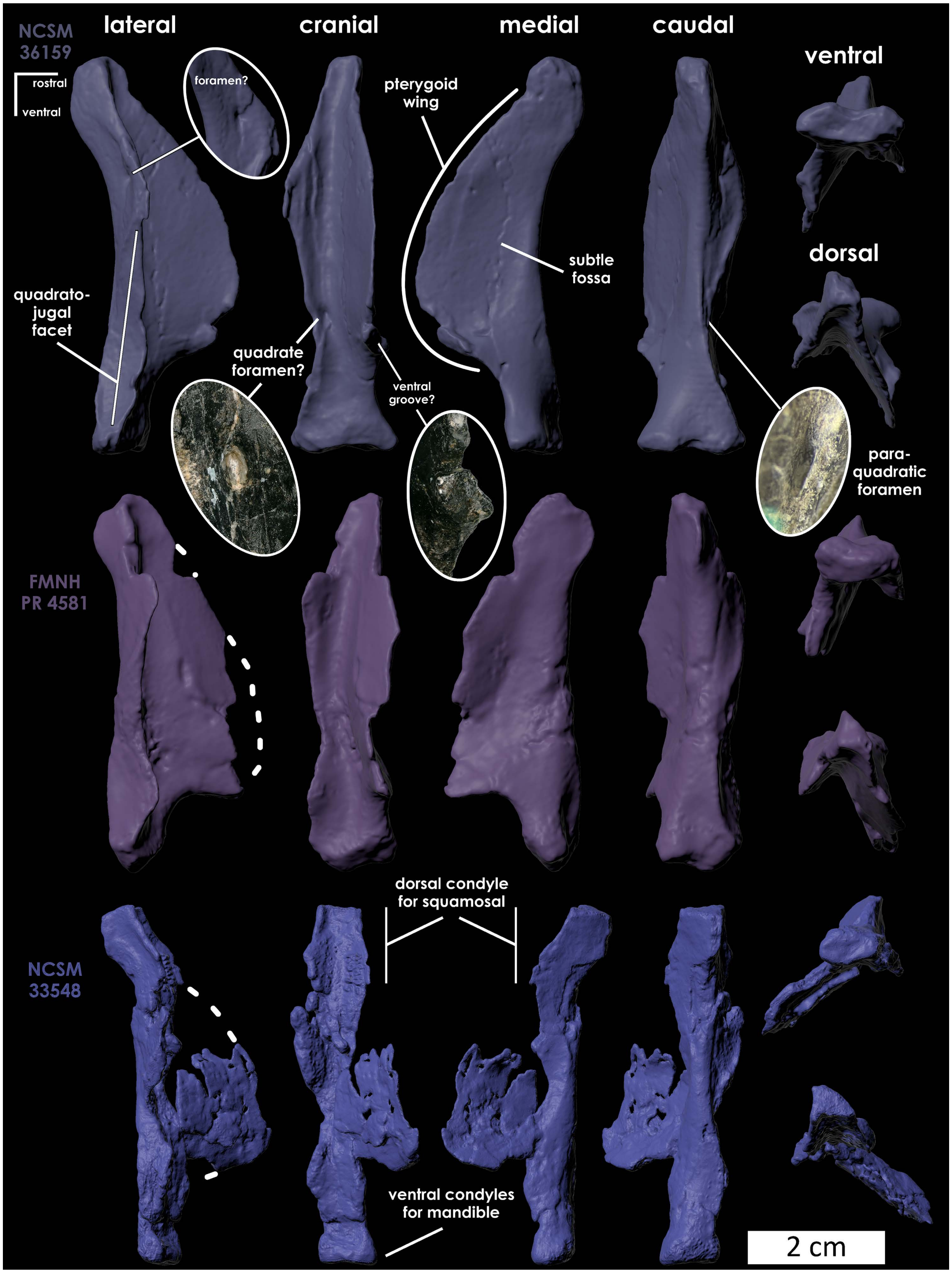

Fig 10. Multiple quadrates of *Fona herzogae*. Dashed lines represent missing bone.

https://doi.org/10.1371/journal.pone.0353169.g010

Oryctodromeus possess a smaller ventromedial condyle and a larger ventrolateral condyle. However, in *Orodromeus,* the smaller medial condyle is aligned with the quadrate shaft [29], whereas in *Oryctodromeus,* the larger lateral condyle is aligned with the quadrate shaft.

Squamosal

Preservation: Two well-preserved, complete, right squamosals are represented from the Mini Troll locality (NCSM 36163 and 36164).

General shape: The squamosal is a crescent-shaped bone characterized by a central body with four processes that project rostrally, rostroventrally, medially, and caudoventrally (Fig 11).

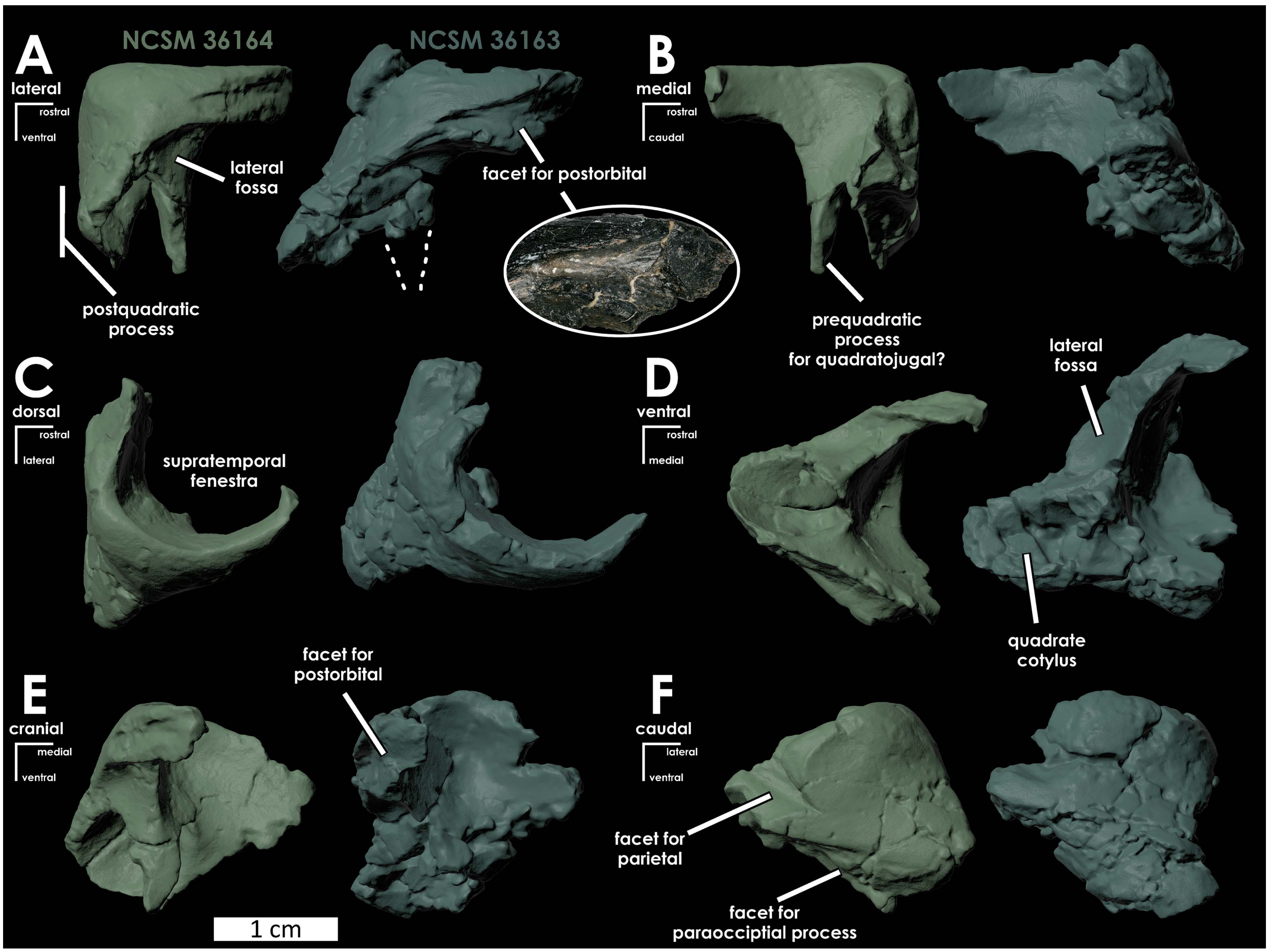

Fig 11. Squamosals of *Fona herzogae*. Dashed lines represent missing bone.

https://doi.org/10.1371/journal.pone.0353169.g011

Articulations: The squamosal forms the caudodorsal margin of the infratemporal fenestra and the caudolateral margin of the supratemporal fenestra. It connects to the postorbital rostrodorsally, the quadrate ventrally, the exoccipital caudoventrally, the parietal mediodorsally, and possibly the supraoccipital caudomedially.

Rostral process: The longest process of the squamosal that is preserved is the rostral process, which connects to the caudal process of the postorbital. The process emerges at the dorsolateral corner of the central body and thins mediolaterally as it projects rostrolaterally before curving rostromedially to create a crescent shape in dorsal view. This strong rostromedial curvature also appears to occur in *Oryctodromeus* and *Orodromeus,* but appears more rostrocaudally straight in *Haya* [25], *Th. neglectus* [18], *Hypsilophodon* (NHMUK R197)*, Lesothosaurus* [3], and especially *Changmiania,* in which the rostral process is rostrally elongated and straight due to the elongation of the dorsal margin of the infratemporal fenestra [23]. There is a distinct triangular groove that begins at the mid-point along the length of the rostral process and widens dorsoventrally as it extends rostrally, creating a slot for the caudal process of the postorbital.

In dorsal view, the dorsal surface of the rostral process is mediolaterally convex and rostrocaudally flat across the entire length of the process, whereas in ventral view, the ventral surface is divided by a rostrocaudally oriented, well-defined, sharp ridge.

Prequadratic process: In ventral view, the prequadratic process is only preserved on NCSM 36164 and is located at the centroid of the element. It is unclear if the full extent of the process is preserved.

Postquadratic process: The postquadratic process is dorsoventrally compressed and projects caudoventrally and somewhat laterally from the main body of the squamosal. The dorsal surface of the process is mediolaterally convex, and the ventral surface is deeply concave, forming the quadrate cotyle.

Medial process: The medial process of the squamosal is short and stout and projects rostromedially from the main body of the squamosal. Its dorsal surface bears a deep triangular slot for the caudal process of the parietal. Between the medial and postquadratic process is the caudal margin of the squamosal, which bears a mediolaterally elongate groove that buttresses the dorsal margin of the paraoccipital process.

Ventral ridges: In ventral view, three ridges radiate from the base of the prequadratic process, contributing to the lateral fossa, supratemporal fenestra, and the quadrate cotyle. In ventral view, the ventral ridge that runs across the rostral process extends rostrally from the base of the prequadratic process. It becomes dorsoventrally shorter, attenuating at the rostral third of the process, transitioning into the mediolaterally compressed ventral margin of the postorbital slot. The edge of this ridge represents the caudodorsal margin of the infratemporal fenestra.

The ventral ridge that runs across the postquadratic process extends caudolaterally from the base of the prequadratic process, becoming dorsoventrally shorter until merging with the lateral margin of the postquadratic process near its caudolateral tip.

A third ventral ridge runs from the tip of the postquadratic process to the tip of the medial process to form a facet for the dorsal margin of the paraoccipital process.

Fossa: In lateral view, the lateral fossa is located between the rostral and postquadratic processes and dorsolateral to the prequadratic process. It is a rostrocaudally elongate concavity for the origin of the *M. adductor mandibulae externus superficialis* [7].

Parietal

Preservation: Two well-preserved fused parietals are represented by *Fona* (NCSM 36148 and FMNH PR 4581) (Fig 12).

General shape: The parietal is a dorsoventrally compressed, hourglass/saddle-shaped bone characterized by four processes, a convex dorsal surface, and a concave ventral surface.

Articulations: The parietal forms the medial half of the supratemporal fenestrae and connects with the frontals rostrally, the postorbitals rostrolaterally, the squamosals caudolaterally, the supraoccipital caudoventrally, and possibly the laterosphenoid rostroventrolaterally.

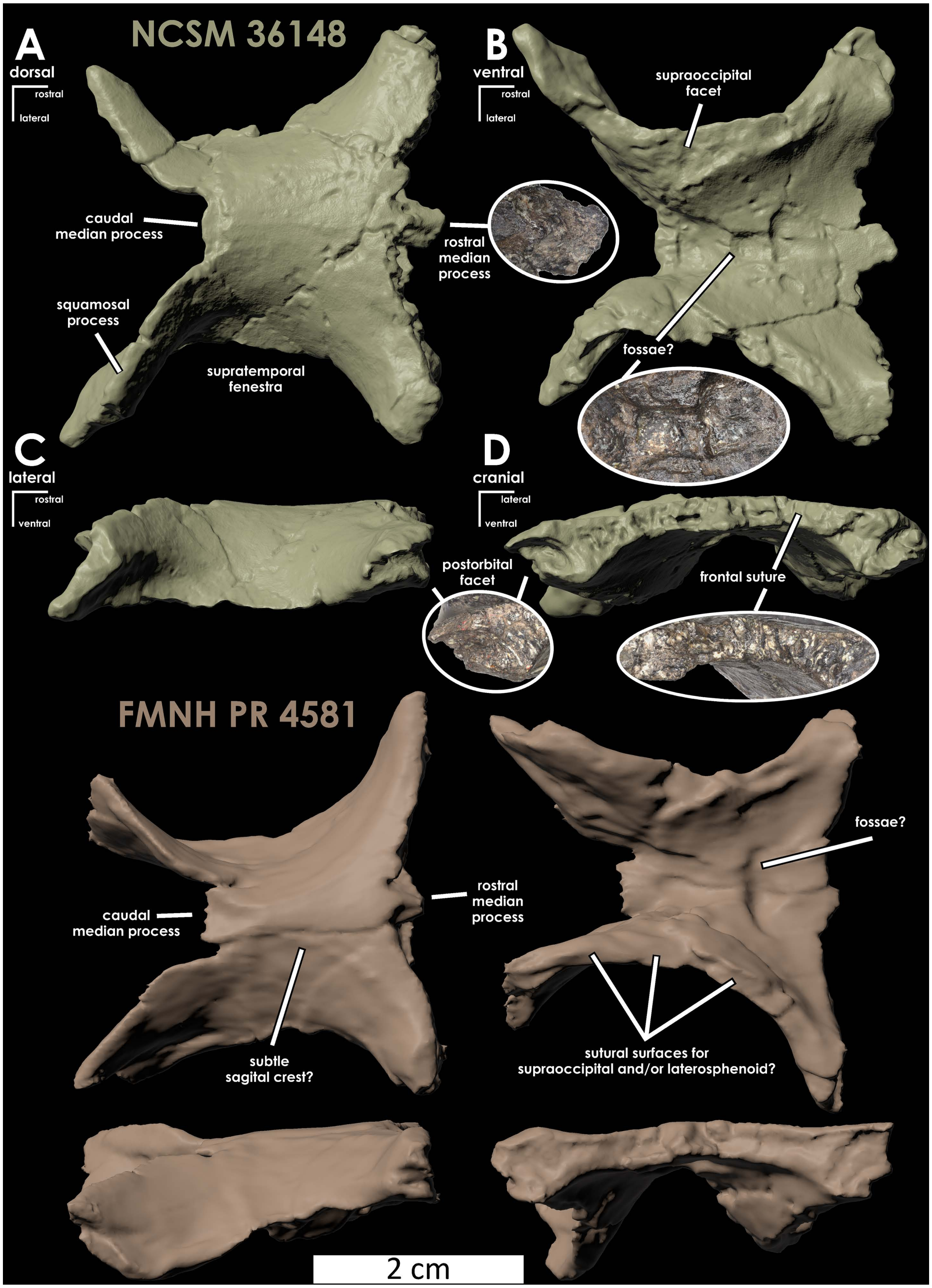

Fig 12. Parietals of *Fona herzogae*.

https://doi.org/10.1371/journal.pone.0353169.g012

Dorsal surface: In dorsal and ventral views, the parietals form a transversely constricted hourglass shape. The dorsal surface of NCSM 36148 is smooth, and there is no visible suture between the contralateral elements. The dorsal surface is flat rostrocaudally and strongly convex mediolaterally, forming a saddle shape. There is no indication of the sagittal crest that is present in most early diverging ornithischians, nor is there a narrow shelf, which is reported as intraspecifically variable in some individuals of *Hypsilophodon*, *Convolosaurus*, and *Dryosaurus* [29]. A complete lack of a sagittal crest is a condition shared only by *Lesothosaurus* and *Changmiania* [3,23], but is extremely reduced on *Orodromeus* (MOR 1136). Additionally, there is no ridge on the dorsolateral surface with a deep ventral excavation demarcating the insertion of the *M. pseudotemporalis superficialis* and *M. adductor externus medius*, as occurs in *Dysalotosaurus* [67]. FMNH PR 4581 does preserve a subtle sagittal crest, suggesting this feature may be ontogenetically or intraspecifically variable.

Rostral process: The rostrolateral corner of the parietals forms a dorsoventrally flat, mediolaterally broad process that projects outward rostrolaterally to contact the frontal rostrally and postorbital rostrolaterally.

Rostral margin: The rostral margin of the parietals forms a series of interdigitating sutures, and in dorsal view, the contact between this margin and the caudal margin of the frontals bows slightly caudally. This condition is shared by most early diverging ornithischians except *Changmiania*, in which the condition is reversed [23], and *Lesothosaurus,* in which the contact is mediolaterally straight [3]. In rostral view, the rostral margin is at its most narrow extent along the midline, becoming dorsoventrally thicker laterally towards the postorbital contact. This contact is formed by a deep pocket into which the caudal margin of the dorsal process of the postorbital slots, as occurs in *Dysalotosaurus* [67].

Rostral tab: The median process is a small tab-like projection that emerges rostrally from the midline, shared by *Th. neglectus* and *Te. tilletti* [4,18]. It is wedge-shaped like a locomotive cowcatcher, and its contact with the frontal is limited to the ventral surface, where it inserts into a small groove at the caudomedial corner. This process is either absent or not visible in dorsal view in *Haya* [25]. It is present in *Dysalotosaurus* [67], where the contact with the frontals extends to the dorsal surface, allowing it to be visible in articulation, a condition possibly shared by some specimens of *Hypsilophodon* [7].

Caudolateral process: The caudal margin of the parietals is deeply inset, forming a "V" shape in dorsal view. This condition is shared by all other small-bodied early diverging ornithischians (SBEDOs) except for *Changmiania* and early diverging ceratopsians (e.g., *Yinlong* and *Psittacosaurus*), in which the caudal margin is mediolaterally straight [23,49,68].

The caudolateral corners of the parietal form a mediolaterally thin process that curves around the caudomedial border of the supratemporal fenestra to contact the squamosal. The contact with the squamosals consists of a short triangular slot joint, in which the rostrolateral face of the caudolateral process slots into an accepting triangular depression on the squamosal medial process. This slotting condition is similar to *Haya*. However, the caudolateral process of *Haya* exhibits a drooping, pendent shape with broader dorsoventral margins, and contact with the squamosal occurs along the ventral tip of the process [25]. These conditions are dissimilar to *Th. neglectus*, in which the caudolateral process extends much further ventrally and appears to wrap around the medial process of the squamosal [18]. At the midline junction between these two processes, a small, short caudal prong exists, a condition shared with *Th. neglectus.*

Ventral surface: In ventral view, the parietal is deeply concave mediolaterally to form the roof of the braincase. On the lateral edges of the ventral surface, there is an elongate groove that is mediolaterally wider prior to the caudolateral process and narrows rostrally toward the postorbital contact. This groove served to accept the supraoccipital. The parietals are not fused to the supraoccipital as they are in *Dysalotosaurus* and at least one specimen of *Zephyrosaurus* [67,69].

There is no sign of a parietal contact for the laterosphenoid, as occurs in *Hypsilophodon* [7], *Zephyrosaurus* [21], and *Te. tilletti* [4]. There is also no sign of the caudally projecting tab present along the caudal margin of the midline in *Th. neglectus* [18] and *Dysalotosaurus* [67].

The ventral surface of the parietal is excavated with multiple depressions of variable diameters, the largest of which occurs along the caudal margin at the midline. This structure also occurs in *Dysalotosaurus,* which houses two pineal foramina that seem to pierce the parietal [67]. Similar binary depressions are present and located closer to the centroid

of the parietal body in *Hypsilophodon,* and in *Leaellynasaura,* they are located further rostrally near the frontal contact [69,70].

Ridges and fossa: The parietals lack a series of caudodorsally inclined ridges on the lateral surface and a pronounced fossa on the dorsal surface of the caudal process, which are present in *Th. neglectus* [18]. They also lack the flattened triangular-shaped surface that is present at the junction between the sagittal crest and the rostral margin in *Th. neglectus* and *Haya* [18,25].

Frontal

Preservation: *Fona* is represented by a partially preserved pair from NCSM 33548 and a well-preserved complete right frontal (NCSM 36136). A smaller, well-preserved, partially complete left frontal (NCSM 36137) was recovered from the Last Chance locality and is referred only to the level of Thescelosaurinae (Fig 13).

General shape: The frontal is a dorsoventrally flat, rostrocaudally elongate, rectangular bone that bows slightly dorsally, broadens slightly caudally, and narrows slightly rostrally.

Articulations: The frontal forms the majority of the orbital dorsal margin, a condition shared by most early diverging ornithischians except for some ankylopollexians and some derived neornithischians (e.g., *Zalmoxes,* where the frontals only contribute to the caudal half of the orbit [39,40], or do not contribute to the orbital margin at all, like in *Zalmoxes shqiperorum* [71]. The frontal connects to the nasals rostrally, the prefrontal rostrolaterally, postorbital caudolaterally, the parietal caudally, and the laterosphenoid caudoventrally.

Medial surface: The medial sutural surface for the contralateral frontal is mediolaterally flat and marked by rostrocaudally oriented sutural scars. In dorsal view, it begins at the caudomedial corner and runs rostrally for the majority of the length of the frontal length, terminating near the rostral end as the medial margin becomes dorsoventrally thinner and angles rostrolaterally by 130 degrees. This angled margin forms a "V" shaped junction with the contralateral frontal that wedges between the nasals. This condition is shared by *Th. neglectus* [18] and *Haya* [25].

Prefrontal contact: The sutural surface for the prefrontal is a rostrocaudally oriented groove located along the rostrolateral margin. It is weakly concave and broadens rostrally to form a facet that slips into the obliquely oriented tongue-and-groove joint located at the caudomedial portion of the prefrontal.

Orbital margin: The orbital margin forms the lateral edge of the frontal and contributes to more than 25% of the total length of the frontal, a condition seen in most early diverging ornithischians with the exception of *Th. neglectus* [18,60], as well as some early diverging iguanodontians, such as *Muttaburrasaurus* [72]. It is dorsoventrally thin and characterized by rugose, laterally directed striations, which likely served as the attachment site for connective tissues that stretched between the frontal and the supraorbitals [73]. A rugose orbital margin is a condition shared by a number of early diverging ornithischians such as *Haya*, *Zephyrosaurus*, TMP 2003.012.0135*, Orodromeus,* the Kaiparowits orodromine [18,21,28,29,61,74,75], early diverging ceratopsians (e.g., *Archaeoceratops* and *Liaoceratops*) [50,76], and the rhabdodontid *Zalmoxes robustus* [71].

Postorbital contact: The postorbital contact is located at the caudolateral corner of the frontal and possesses a dorsolaterally facing articulation.

The facet for the dorsal head of the laterosphenoid is located ventromedial to the postorbital contact, at the caudal termination point of the crista cranii. It continues onto the postorbital as in the early diverging ornithischians *Agilisaurus, Jeholosaurus, Lesothosaurus, Th. neglectus, Zephyrosaurus*, and the Kaiparowits orodromine [18,21,22,24,46]. This condition is also likely present in *Orodromeus* [26].

Dorsal surface: The dorsal surface of the left and right frontals of NCSM 33548 bear shallow rostrocaudally elongate depressions that are slightly offset from their midline medial contacts. This depression is either formed entirely or accentuated by the dorsal swelling of the orbital rim. It is more prominent on the left of NCSM 33548, possibly due to its more

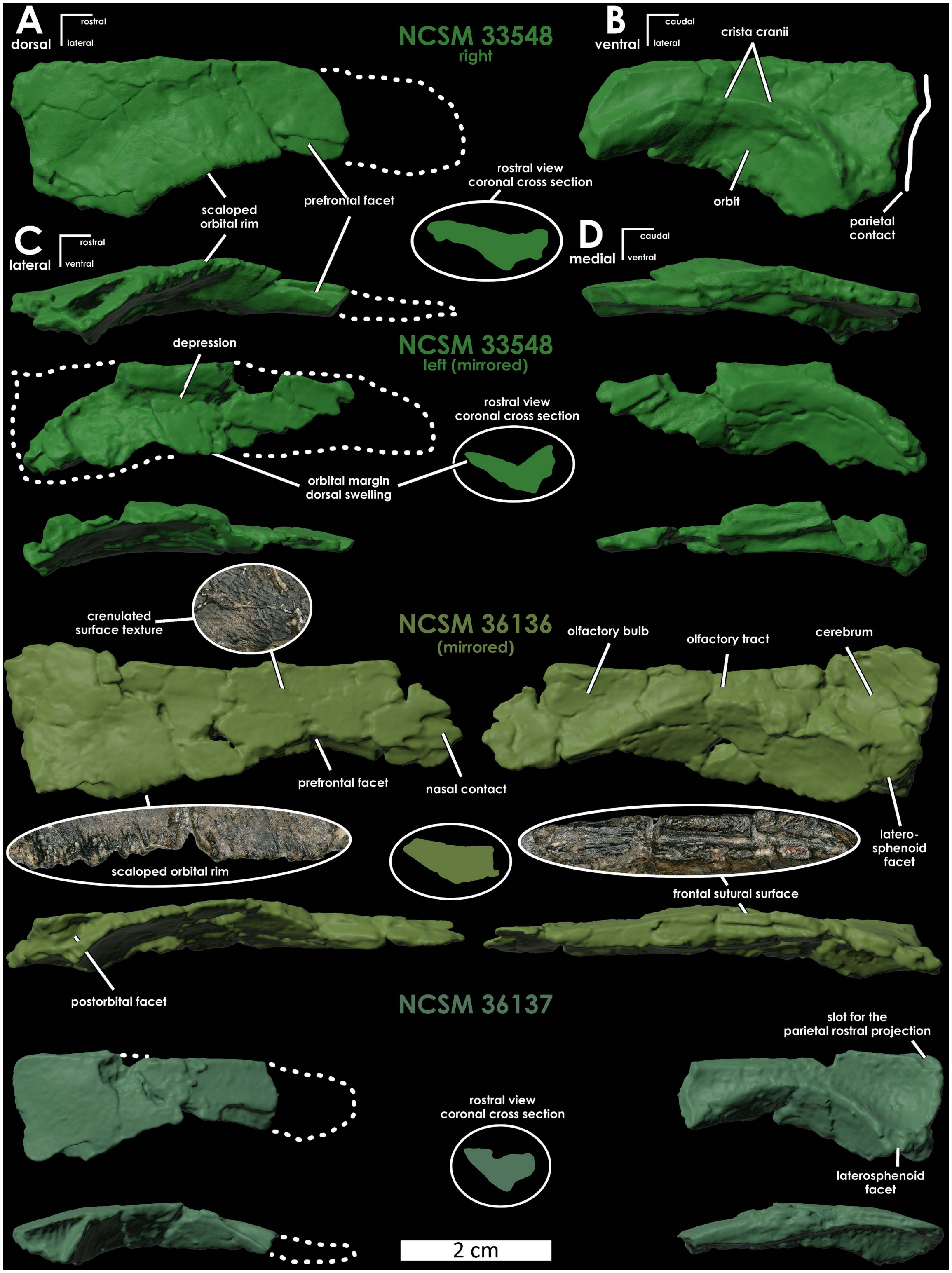

Fig 13. Frontals of *Fona herzogae*. Dashed lines represent missing bone.

https://doi.org/10.1371/journal.pone.0353169.g013

extensive damage, and is absent on NCSM 36137. It is possibly present on NCSM 36136, constrained to a small, subtle dimple towards the caudal region of the dorsal surface.

In dorsal view, the caudal contact with the parietals bow slightly caudally, as occurs in *Haya, Lesothosaurus, Orodromeus,* and *Hypsilophodon* [7,24,28,29]. In *Th. neglectus,* the medial half of the caudal margin is more prominently caudally inset, and these projections fit into slots on the parietals [18]. In ventral view, there is a small dimple at the ventromedial corner of the frontal that likely housed the rostral projection of the parietal. It is only preserved on NCSM 36137.

Ventral view: In ventral view, the crista cranii runs rostrocaudally and bows medially to form the orbital margin, which is not as laterally broad as in *Te. tilletti* [4]. Rostromedial to the crista cranii is a concavity for the olfactory bulb, which connects, via the olfactory tract, to the caudomedial concavity that marks the space for the rostral portion of the cerebrum [69].

Prefrontal

Preservation: Two well-preserved, nearly complete, right prefrontals are preserved from NCSM 33548 and NCSM 36151 (Fig 14). A second prefrontal fragment was recently identified from the Mini Troll locality (NCSM 36150) (S1.11 File), and is not discussed here due to its poor and incomplete preservation.

General shape: The prefrontal is a triradiate bone that appears as a dorsally bowed scalene triangle in lateral view. Caudally, it begins as a dorsoventrally thin tip that broadens dorsoventrally towards its rostral end. The dorsal and ventral surfaces are mediolaterally straight, and the medial surface is deeply concave dorsoventrally.

Articulations: It forms the rostrodorsal margin of the orbit and connects to the frontal caudomedially, the nasal rostromedially, the dorsal margin of the lacrimal ventromedially, and the supraorbital laterally.

Medial surface: The medial surface of the prefrontal is deeply concave, becoming less prominent as it tapers caudally, as in *Hypsilophodon, Haya,* and *Zephyrosaurus* [7,21,28]. The medial region of the caudal process is characterized by a thin, obliquely oriented tongue-and-groove joint for articulation with the rostrolateral portion of the frontal.

Rostral process: The rostral process is a dorsoventrally thin sheet of bone that tapers to a pointed triangular tip. Its rostromedial edge would have bordered the caudolateral margin of the nasal and would likely have been positioned dorsal to the lacrimal, as in most early diverging ornithischians [77–79]. This is in contrast to *Parksosaurus,* in which the triangular tip of the prefrontal dives rostroventrally to insert between the lacrimal and the dorsal process of the maxilla, nearly preventing contact between the two [80,81]. Barta and Norell [25] notes a slight contact between the maxilla and the prefrontal in *Haya*, which appears to be absent in *Fona*, as inferred by the apparent lack of a sutural surface on the dorsolateral portion of the prefrontal.

Ventral process: The ventral process extends from the rostroventral region and would have likely been partially underlapped by the lacrimal, as in *Hypsilophodon* (NHMUK PV R2477), *Haya,* and *Orodromeus* [28,29]. As in *Th. neglectus* [18], there is a slightly concave depression at the distal end of the ventral process, which likely articulated with the lacrimal.

Lateral view: In lateral view, the articulation for the supraorbital occurs at the swollen region dorsal to the lacrimal contact near the dorsal margin. It lacks the rugosity that characterizes the prefrontals in *Th. neglectus* [79].

Foramen: The quantity of foramina varies between individuals. In dorsal view of NCSM 33548, at least three prefrontal foramina are located on the dorsal surface, medial to the supraorbital contact, with a fourth located high on the lateral surface. In contrast, NCSM 36151 bears only a single foramen on the dorsal surface, which opens rostromedially but does not perforate the dorsal surface. Rather, in medial view, a subtle ridge demarcates the internal path of the canal, which travels caudoventrally before opening on the ventral surface of the prefrontal. A prefrontal foramen was previously reported as one of the five autapomorphies of *Th. neglectus* [79], but also occurs in at least one specimen of *Haya* (MPC-D 100/3181) [25], and *Orodromeus* (MOR 1141) [26].

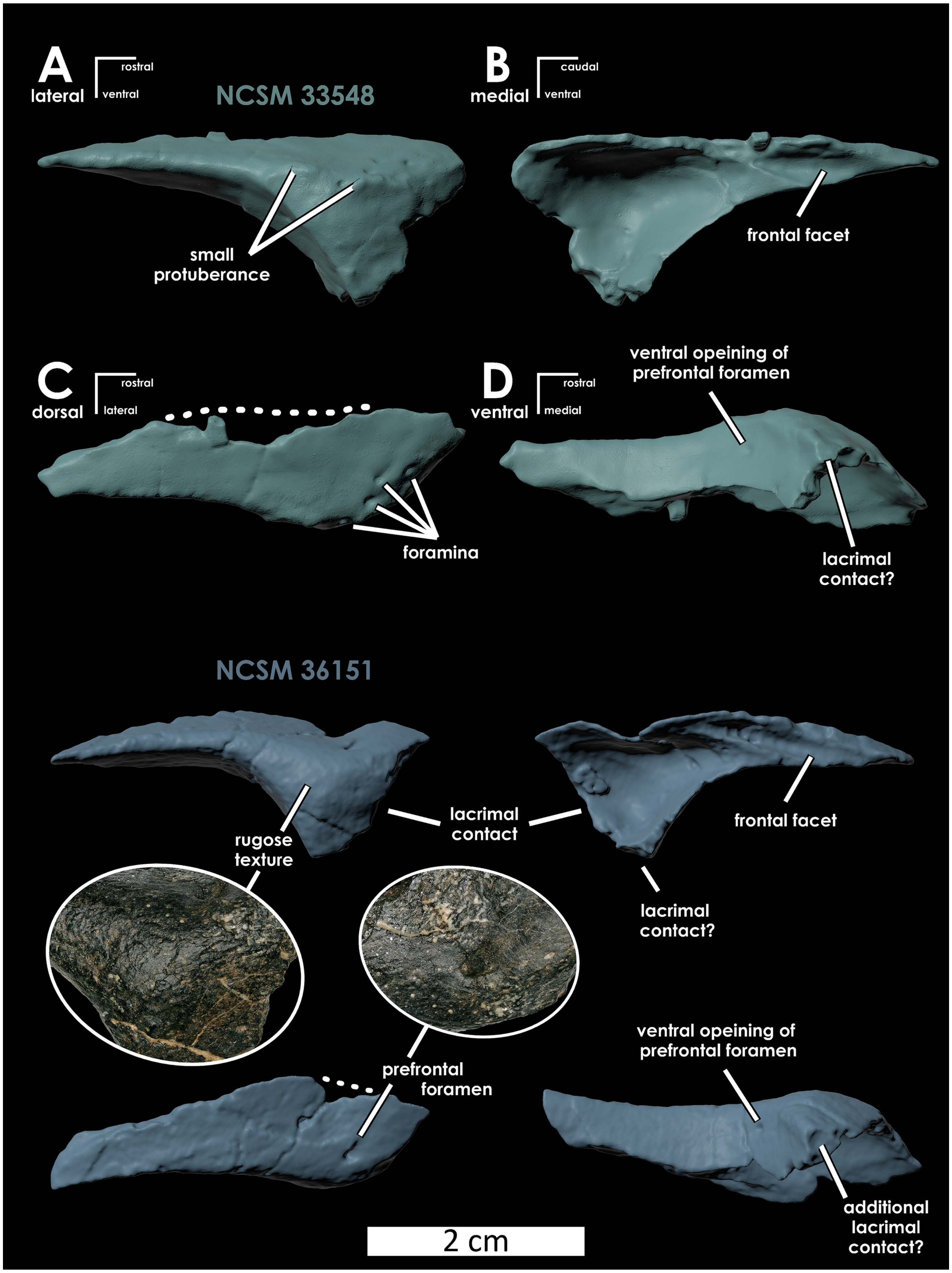

Fig 14. Prefrontals of *Fona herzogae*. Dashed lines represent missing bone.

https://doi.org/10.1371/journal.pone.0353169.g014

Lacrimal

Preservation: A single incomplete left lacrimal is preserved from the Mini Troll locality (NCSM 36172).

General shape: The lacrimal is a rotated "J" shaped bone that is slightly mediolaterally compressed and characterized by a prominent perforation that pierces through the element (Fig 15).

Articulations: The lacrimal forms the dorsal margin of the antorbital fenestra and the rostral margin of the orbit. It contacted the prefrontal dorsally, the nasal rostrodorsally, the jugal, and possibly the maxilla and palatine ventrally, and possibly the premaxilla rostrally.

Dorsal surface: In lateral view, the dorsal margin of the vertex is evenly rounded as in *Hypsilophodon* (NHMUK R2477) [43], but in contrast to the sharper "L" shaped angle formed by the lacrimal of *Th. neglectus* (NCSM 15728). Additionally, this region is relatively featureless and lacks the rounded dorsolateral boss present in *Th. neglectus* (NCSM 15728). In dorsal view, a small groove can be seen near the vertex, which would have facilitated contact with the prefrontal.

Rostral lateral surface: The lateral surface of the lacrimal rostral to the vertex is slightly concave dorsoventrally to form the dorsal rim of the antorbital fenestra. The ventral margin of this depression likely continued as a thin lamina, although much of its ventral extent is missing.

Rostral medial surface: The medial surface of the lacrimal rostral to the vertex bears a distinct concavity, which would have formed the contact with the lateral rim of the nasal. In contrast, the lacrimal of *Th. neglectus* is overlapped laterally by the nasal [18].

Caudal lateral surface: Caudal to the vertex, the preserved portion of the lateral surface is weakly convex dorsoventrally (as figured), until the caudal end, which bears a small concavity that likely accepted the rostral process of the jugal.

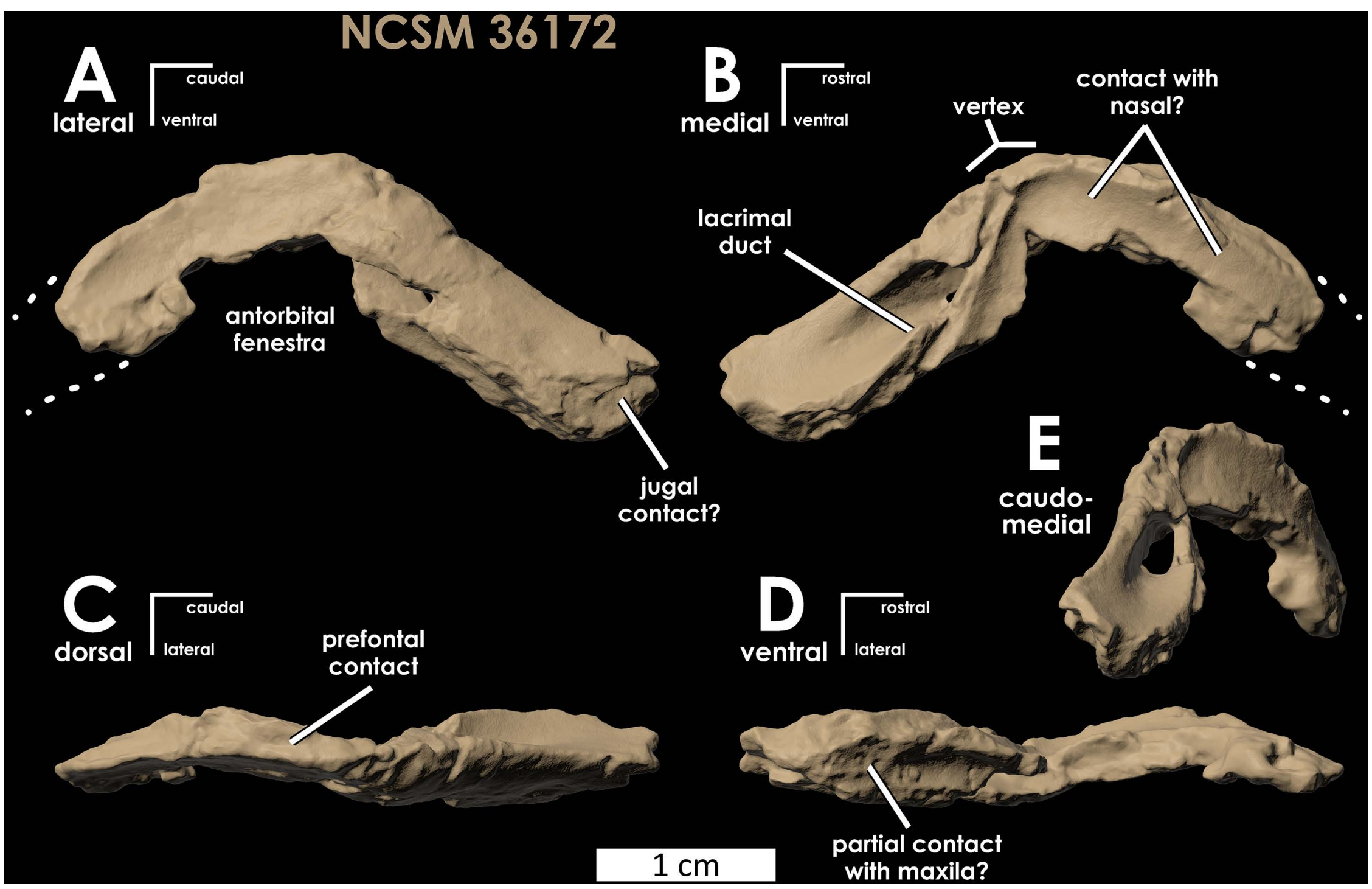

Fig 15. Lacrimal of *Fona herzogae*. Dashed lines represent missing bone.

https://doi.org/10.1371/journal.pone.0353169.g015

Caudal medial surface: The medial surface is deeply concave to form the path of the prominent lacrimal duct. The duct pierces through the element rostrally, emerging directly ventral to the vertex. The rostral margin of the duct in medial view is formed by a distinct diagonal groove, which seems to form a demarcation between the rostral and caudal portions of the element. In *Th. neglectus* (NCSM 15728), the lacrimal duct faces more caudally.

Ventral surface: The ventral surface of the lacrimal, caudal to the vertex, bears a distinct rostrocaudally oriented groove that likely contributed to the caudodorsal margin of the antorbital fenestra. The lateral and medial margins of this groove may have borne several thin laminae that draped across the lateral and medial surfaces and contributed to the inner wall of the antorbital fenestra, as in *Th. neglectus* (NCSM 15728). More caudally, this groove may have formed a partial contact with the dorsal border of the maxilla.

Rostrocaudal extent: The rostral extent of NCSM 36172 seems to be incomplete. However, the lateral groove on the nasal of NCSM 36144 is continuously concave from the caudal contact for the lacrimal to the slightly deeper rostral facet with the premaxilla. Therefore, if the entirety of this groove bore the lacrimal and was not interrupted by any contribution from the dorsal border of the maxilla, it would have contacted the caudal process of the premaxilla, a condition shared only with *Jeholosaurus* among members of Thescelosauridae [19]; possibly present among several other phylogenetically unstable SBEDOs (see description of premaxilla and nasal).

Supraorbital

Preservation: Two partially preserved right anterior supraorbitals (= palpebrals) were recovered from the Mini Troll locality (NCSM 36147 and 36146).

General shape: The anterior supraorbital is a triradiate, rostrocaudally elongate, slightly curved, rod-like bone that is mediolaterally wider rostrally and tapers caudally (Fig 16).

Articulations: The anterior supraorbital emerges laterally from the rostrodorsal corner of the orbit, on the prefrontal, and projects caudally.

Lateral surface: The anterior supraorbital is slightly dorsoventrally flattened, and across its length, it bows slightly laterally and slightly dorsally.

Processes: In dorsal view, the rostral end is characterized by two small processes that project medially and rostrolaterally, respectively, forming a concave margin between them that forms the prefrontal contact. The ventral surface of the rostral end is incompletely preserved on both elements, but possibly formed a contact with the lacrimal, as in *Th. neglectus* [18].

Striations: On the well-preserved dorsal surface of NCSM 36146, faint striations are present along the rim of the medial margin. They appear most prominent at the element’s mid-length and are oriented rostrolaterally to caudomedially.

Posterior supraorbital: Several rod-shaped elements were considered to possibly represent a posterior supraorbital (also referred to as an accessory supraorbital, secondary supraorbital, or postorbital palpebral), which are present on *Th. neglectus* [18], *Haya* [25], *Orodromeus* [26], and some specimens referred to *Jeholosaurus* from the STMN (personal observation). However, confident identification was difficult to determine due to the simple and diminutive morphology of these elements, all of which were determined to more likely represent posterior dorsal or anterior sacral ribs.

Supraoccipital

Preservation: A single partially preserved supraoccipital is represented from the Mini Troll locality (NCSM 36165), and a second, poorly preserved supraoccipital is represented from the Manolo locality (FMNH PR 4581) (Fig 17).

General shape: The supraoccipital is a trapezoidal bone in dorsal view. It is characterized by a flat, caudodorsally tilted dorsal surface and a deeply concave ventral surface.

Articulations: It forms the dorsal margin of the foramen magnum and connects to the parietals dorsally, the prootic ventrally, the otoccipitals caudoventrally, and likely the laterosphenoid rostrally, although these contacts do not appear to be preserved.

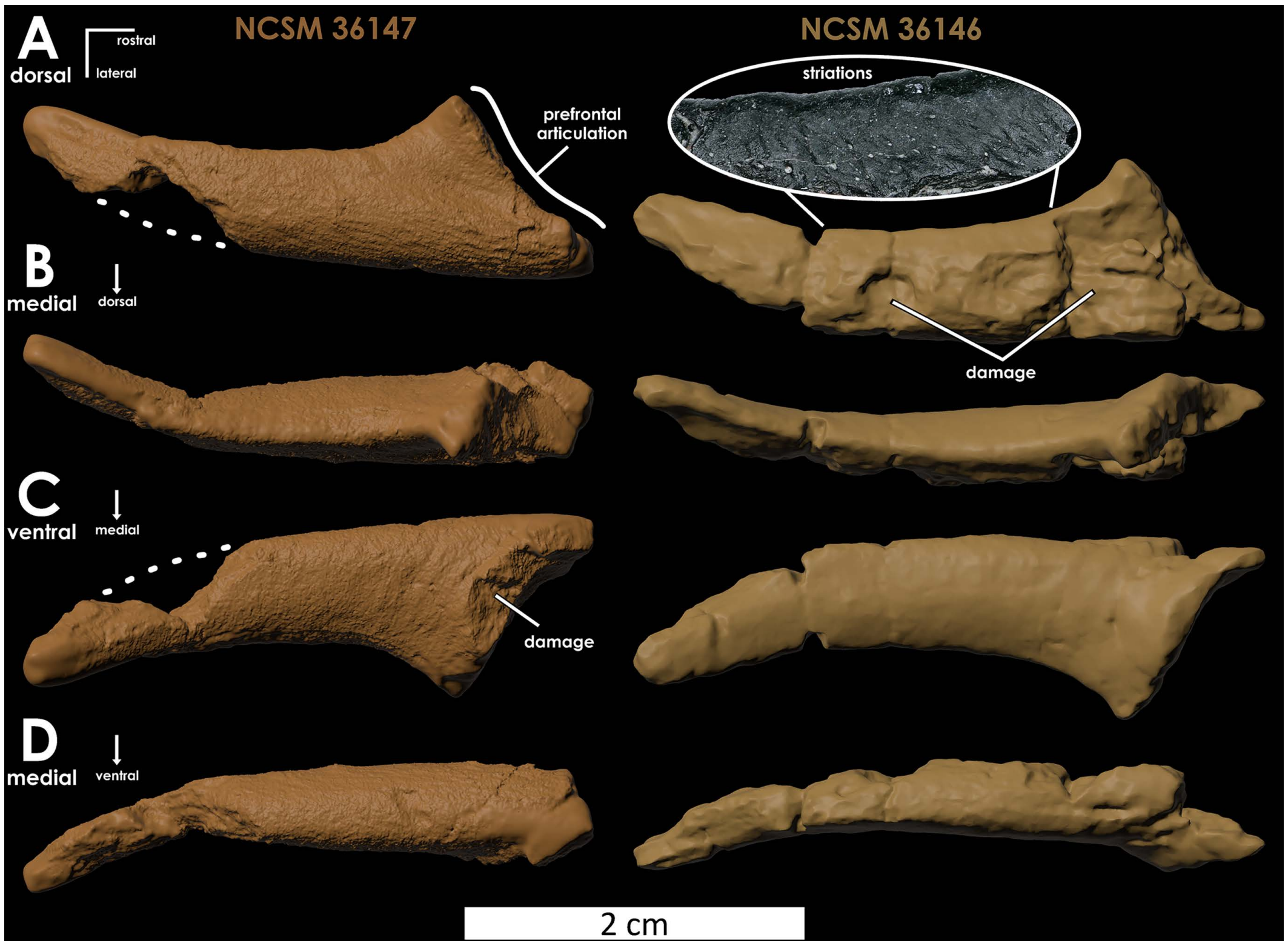

Fig 16. Supraorbitals of *Fona herzogae*. Dashed lines represent missing bone.

https://doi.org/10.1371/journal.pone.0353169.g016

Dorsal surface: The dorsal surface lacks the prominent midline nuchal crest present in many early diverging ornithischians. However, there is a slightly raised rostrocaudally oriented ridge running down the midline, a condition shared by *Th. neglectus* [18], *Th. assiniboiensis* [60], and *Haya* [25]. The presence and/or prominence of this feature may be ontogenetically variable in *Orodromeus* [82: character 77]. A lack of a nuchal crest was also reported in *Changmiania* [23]. There are two subtle concave depressions located lateral to the median ridge. There is no sign of a groove or canal for the vena capitis dorsalis within or near these concavities, as occurs in *Th. neglectus* and *Dysalotosaurus* [18,60,67].

Supraoccipital Foramen: There are no foramina across the dorsal surface, which are present in *Th. neglectus* and *Th. assiniboiensis* [18,60].

Ventral margin: The caudoventral margin contributes to the dorsal rim of the foramen magnum. This condition occurs in most early diverging ornithischians except for *Th. neglectus, Dryosaurus, Dysalotosaurus,* and some iguanodontians (e.g., *Tenontosaurus tilletti*) in which the contribution is significantly reduced or completely absent [4,67].

Rostroventral surface: The rostroventral surface is deeply concave mediolaterally, contributing to the roof of the braincase.

Ventrolateral process: Ventrally, two short, blocky processes emerge from the supraoccipital and swell slightly laterally. In ventral view, these ventrolateral processes have a square profile. Their surfaces are excavated, forming the sutures with the otoccipitals.

Foramen: Centrally, this surface houses the continuation of the posterior semicircular canal from the otoccipitals, which likely travels rostrally through the supraoccipital before meeting at a triple junction with the common crus and the anterior semicircular canal, as in *Dysalotosaurus* [67]. The cross-section of the anterior semicircular canal can be seen in ventral view along the contact with the prootic. The left anterior semicircular canal is bordered by two accessory foramina, one

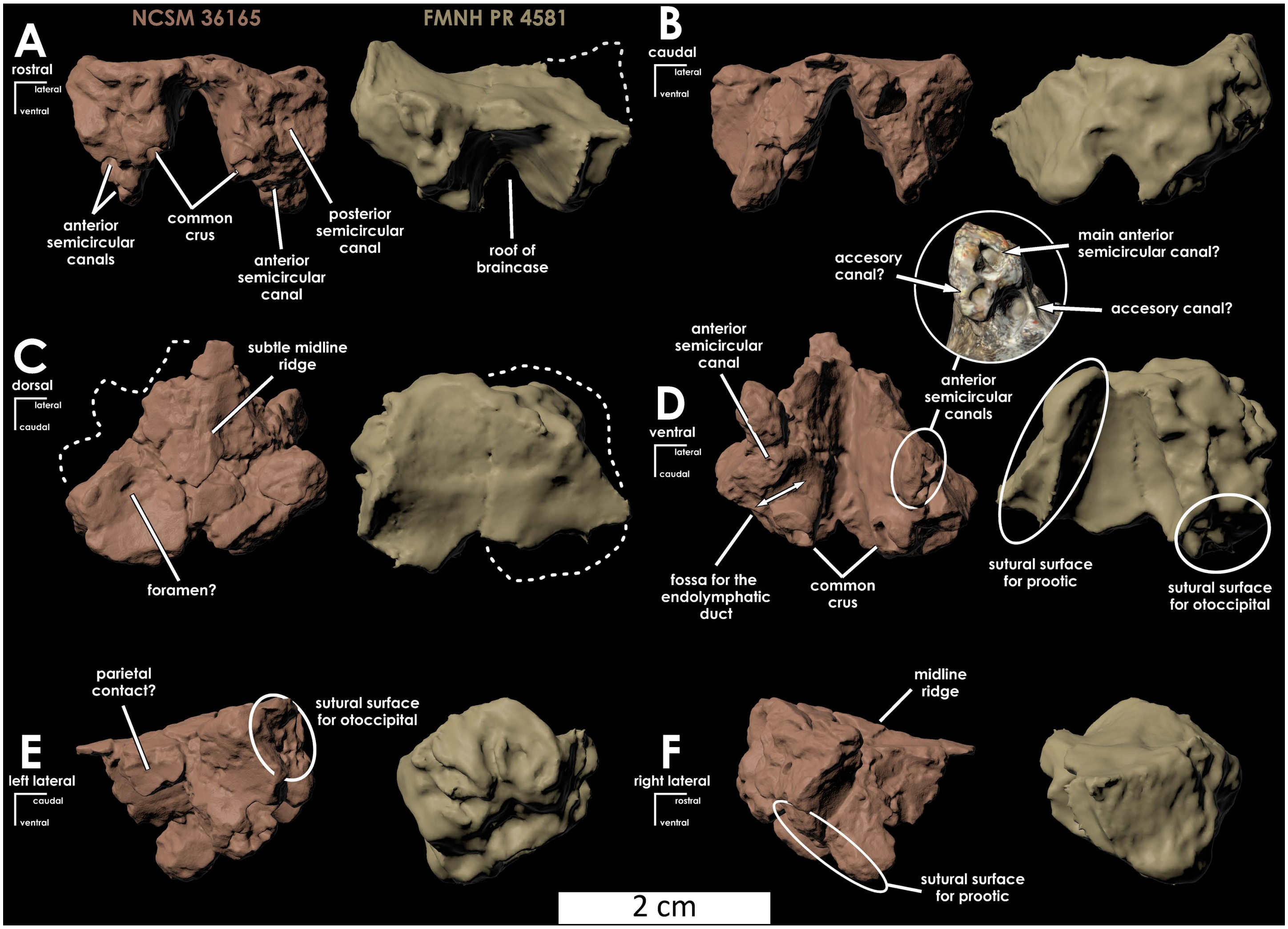

Fig 17. Supraoccipitals of *Fona herzogae*. Dashed lines represent missing bone.

https://doi.org/10.1371/journal.pone.0353169.g017

located caudomedially that is slightly larger and another caudolaterally that is significantly larger, a condition that may be unique to *Fona*. It is unknown if these accessory foramina connect internally with the main branch.

Fossa and common crus: In rostroventral view, two structures are present between the anterior and posterior semicircular canals. Near the ventral margin of the ventrolateral processes are dorsomedially directed fossae for the endolymphatic duct [67], formerly called the fossa subarcuata [69], which continue across the medial surface of the prootic. On the supraoccipital, the endolymphatic duct appears somewhat shallow. This morphology is closer to the description of *Th. neglectus* [18] versus *Orodromeus* and *Hypsilophodon*, which were described or figured as deep and elongate [29,69]. Directly ventral to these fossae are two elevated but short columnar projections that house the common crus, which likely connect internally to the semicircular canals, as in *Dysalotosaurus* [67].

Otoccipital

Preservation: Five otoccipitals are represented in total. Two well-preserved, complete lefts (NCSM 36133 and 36134) and a slightly larger, poorly preserved, partially complete right (NCSM 36134) were recovered from the Mini Troll Locality.

Fona from the Karmic Locality is represented by a mostly complete right (NCSM 33548), and a partially preserved left is represented from the Manolo locality (FMNH PR 4581).

General shape: The otoccipital is a partially flattened, roughly rectangular bone characterized dorsally by a prominent rectangular process that is dorsolaterally inclined and ventrally by a more robust region dominated by cranial nerve openings and a prominent condyle (Fig 18). The demarcation between the dorsally located opisthotic and the ventrally located exoccipital is indistinct and lacks a visible suture.

Articulations: The otoccipital forms the dorsal and lateral margins of the foramen magnum and connects to the basioccipital ventrally, the prootic rostrally, the supraoccipital dorsomedially, the squamosal dorsally, and the proatlas caudally.

Ventral region: The ventral portion of the otoccipital consists of a ventrally directed, mediolaterally constricted pedicle, with a ventral edge that runs rostromedially to caudolaterally. This pedicle emerges from the ventral corner of the rectangular paroccipital process and becomes slightly mediolaterally thicker at its ventral margin. This ventral surface forms the contact with the basioccipital and is densely rugose. The caudal aspect of this pedicle forms a bluntly rounded knob that constitutes the dorsolateral portion of the occipital condyle. A ridge emerges from the dorsal aspect of the occipital condyle and runs dorsally to merge with the tetrahedral boss that likely articulates with the proatlas (Fig 18 B), as occurs in *Th. neglectus* [18]. In *Haya*, this boss is either absent or may be located more ventrally [25]. In medial view, there is a dorsoventrally elongate fossa on the medial surface of the occipital condyle located directly caudal to the medial opening of CN XII. This fossa is not present in *Orodromeus, Oryctodromeus*, or *Th. neglectus*. However, it may be present in *Th. assiniboiensis* (personal observation).

Paroccipital process: The paroccipital process is the largest structure of the otoccipital. It is a rostrocaudally compressed, dorsolaterally elongate, rectangular process that arises dorsolaterally from the main body of the otoccipital. In caudal view, the dorsolateral corner of the process extends ventrally, giving the process a 'pendant' or 'hatchet-shape', as occurs in *Th. neglectus* [18], *Zephyrosaurus* [21], *Haya* [25], *Orodromeus* [26], and *Oryctodromeus* [83].

Along the dorsal margin of the paroccipital process of NCSM 36133, near the dorsolateral corner, there is a subtle dorsal step, a condition shared with *Oryctodromeus* (MOR 1636), *Haya* (MPC-D 100 2019), *Th. neglectus* [18], *Lesothosaurus* [3], *Te. tilletti* [4], and weakly in *Iani* [1]. A dorsal step is absent in the other three otoccipitals that preserve this region, suggesting it may be intraspecifically variable. It is also absent in *Zephyrosaurus* [21], *Hypsilophodon* (NHMUK PV R2477), and *Te. dossi* (FWMSH 93B2).

The central portion of the caudal surface of the paroccipital process bears a subtle bulge running dorsolaterally, a condition shared with *Oryctodromeus* (MOR 1636) and *Orodromeus* (MOR 1136). It is located more dorsally on NCSM 33548, is more prominent on NCSM 36135, and is absent in *Th. neglectus* (NCSM 15728) and *Haya* [25]. The process lacks the broad depression located near the dorsolateral margin of the foramen magnum on *Haya*, which is thought to represent the insertion site for *m. rectus capitis posterior* [25]. The lateral surface of the process also lacks the prominent striations present in *Haya* [25], which are also absent in *Th. neglectus* (NCSM 15728).

Posttemporal fenestra: There is a crescent-shaped indentation located along the dorsal margin of NCSM 36134, which represents the posttemporal fenestra, sometimes referred to as the groove for the vena capitis dorsalis [67]. The placement of this groove on the dorsal margin of the paroccipital process is the same in *Th. neglectus* [18,60], *Dysalotosaurus* [67], *Orodromeus* [29], and *Te. tilletti* [4]. In contrast, this opening is fully enclosed on the paroccipital process in *Zephyrosaurus* [21], *Haya* [28], *Gasparinisaura* [56], *Hypsilophodon* [7], and possibly *Lesothosaurus* [3]. In *Jeholosaurus,* the posttemporal fenestra was initially described as enclosed within the process [22], however recent phylogenetic work has coded it as equivalent to the former condition [74,84]. A groove for the vena capitis dorsalis is absent or possibly very faint on the other three otoccipitals, suggesting that this feature may be ontogenetically or intraspecifically variable in some taxa.

Paroccipital process (rostral surface): The rostral surface of the paroccipital process is marked by a prominent ridge that emerges from the center of the paroccipital process near the distal end and runs ventromedially, reaching its greatest

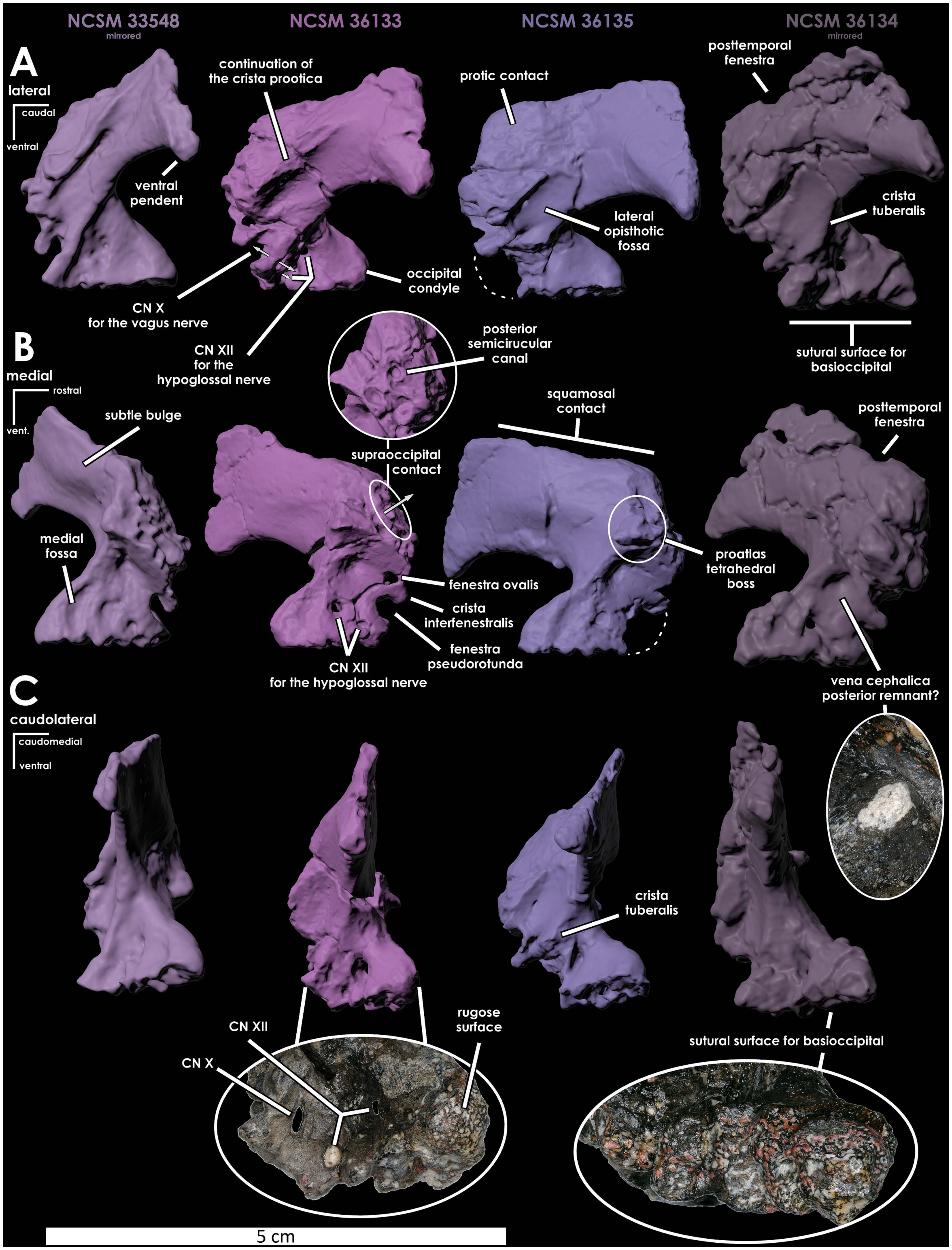

Fig 18. Otoccipitals of *Fona herzogae*. Dashed lines represent missing bone.

https://doi.org/10.1371/journal.pone.0353169.g018

prominence near the ventral margin of the otoccipital, slightly dorsal to the fenestra ovalis. This ridge represents the continuation of the crista prootica of the prootic and does not appear to be as prominent in *Th. neglectus* [18]. Ventral to this ridge is the lateral opisthotic fossa. This fossa also occurs in *Orodromeus* and *Zephyrosaurus* [21,29], but is absent in *Th. neglectus* [18]. On NCSM 33548, the crista prootica is strongly inflected ventrally, creating a lateral opisthotic fossa with much steeper margins.

Dorsal to the crista prootica is a triangular contact for the prootic. At the ventromedial corner of the crista prootica is the foramen for the lateral semicircular canal. This canal likely travels caudally through the element to meet the posterior semicircular canal in the posterior ampullary recess, as occurs in *Dysalotosaurus* [67].

Supraoccipital contact: On the medial-most margin of the paroccipital process, dorsal to the fenestra ovalis and medial to the proatlas boss, is the contact with the supraoccipital, which bears an excavated surface to facilitate this connection, as in *Zalmoxes* [40]. This surface bears the foramen for the posterior semicircular canal, which continues onto the supraoccipital.

Crista tuberalis: In ventral view, the crista tuberalis, also referred to as the metotic strut [67] or the crista metotica [4], is a prominent ridge that emerges dorsal to the rostral end of the contact with the basioccipital and runs dorsolaterally to merge with the ventral margin of the paroccipital process. The ventral portion of this ridge is pierced by a foramen (CN X) for the vagus nerve, which exits the braincase through the fenestra pseudorotunda and travels caudally through CN X. The foramen is oriented more laterally as in *Th. neglectus* [18] and *Dysalotosaurus* [67], and in contrast to the more caudal orientation in *Haya* [25].

Fenestra pseudorotunda: The fenestra pseudorotunda, also referred to as foramen metoticum [69] or the fenestra metotica [4], is the largest opening of the otoccipital. As in *Th. neglectus* [18] and *Dysalotosaurus* [67] it is not fully enclosed. This fenestra probably facilitated the passage of three cranial nerves from the braincase, which includes the glossopharyngeal nerve (CN IX), the spinal accessory nerve (CN XI), and the vena cephalica posterior [67].

Fenestra ovalis: Directly dorsal to the fenestra pseudorotunda is a small, rostromedially projecting process called the crista interfenestralis, which separates the fenestra pseudorotunda from the fenestra ovalis. The fenestra ovalis, which sits directly dorsal to the crista interfenestralis, probably served as the insertion point for the stapes, as in *Th. neglectus* [18].

Vestibule: Dorsomedial to the fenestra ovalis is a deep concavity that penetrates the paroccipital process known as the vestibule, which likely connects to the lateral and posterior semicircular canals in a posterior ampullary recess, as occurs in *Dysalotosaurus* [67].

Caudomedial surface of ventral portion: In caudomedial view, the caudomedial surface of the ventral portion of the otoccipital is broadly concave and forms the caudolateral wall of the braincase. As in *Th. neglectus* [18] this concave region is pierced by two closely spaced foramina, representing the rostral and caudal branches of the hypoglossal nerve (CN XII). The foramen located more dorsolaterally housed the caudal ramus of the hypoglossal nerve, whereas the more ventromedially positioned foramen housed the rostral ramus. This condition is not present in *Jeholosaurus,* which is reported to have a single foramen rather than two for the hypoglossal nerve. It is also dissimilar to the condition in *Haya,* in which the two foramina are oriented rostrocaudally to each other [25]. Additionally, there are two foramina in *Te. tilletti* that Thomas [4] tentatively assigned as housing the glossopharyngeal nerve (CN IX) and the spinal accessory nerve (CN XI). These foramina are located in a similar position to the ventral-most foramen of *Fona*, possibly indicating more variation in cranial nerve morphology across early diverging ornithischians than previously recognized.

Vena cephalica posterior: There is a small shallow fossa present on the medial surface, slightly dorsal to CN X and ventral to the boss that articulates with the proatlas, likely representing the same depression on the otoccipital of *Th. neglectus* noted by [18]. This fossa may represent the remnant of the vena cephalica posterior [69] (termed the posterior cerebral vein in Sobral et al. [67]), although this feature may have been previously misinterpreted [67].

Prootic

Preservation: A left (NCSM 36157) and two well-preserved, equally sized right prootics (NCSM 36155 and NCSM 36156) are represented from the Mini Troll locality. A single well-preserved right prootic (NCSM 33548) is represented from the Karmic locality.

General shape: The prootic is a mediolaterally compressed, complex, quadrangular-shaped bone (Fig 19 and 20). It is characterized by extensive rugose sutural surfaces lining the edges and corners, a caudodorsally directed sheet of bone, and a large foramen that pierces directly through the center of the element.

Articulations: The prootic formed the mid-lateral walls of the braincase and connects with the otoccipital caudally, the basioccipital caudoventrally, the basisphenoid rostroventrally, the laterosphenoid rostrally, and the supraoccipital dorsally.

Dorsal surface: In dorsal and lateral views, the dorsal surface of the prootic is smooth, slightly rostrocaudally concave, and inclined ventrolaterally. At the caudodorsal corner of the prootic, the surface emerges into a triangular sheet of bone called the caudal process (posterior process in Oelrich [85]), that overlaps a triangular suture on the paraoccipital process of the otoccipital. The subtle concavity of the dorsal surface may represent the fossa that occurs with greater prominence in the sauropodomorph *Panphagia protos* [86], which was interpreted as representing either the recessus tympanicus dorsalis or an attachment site for the *M. adductor mandibularis externus profundus*.

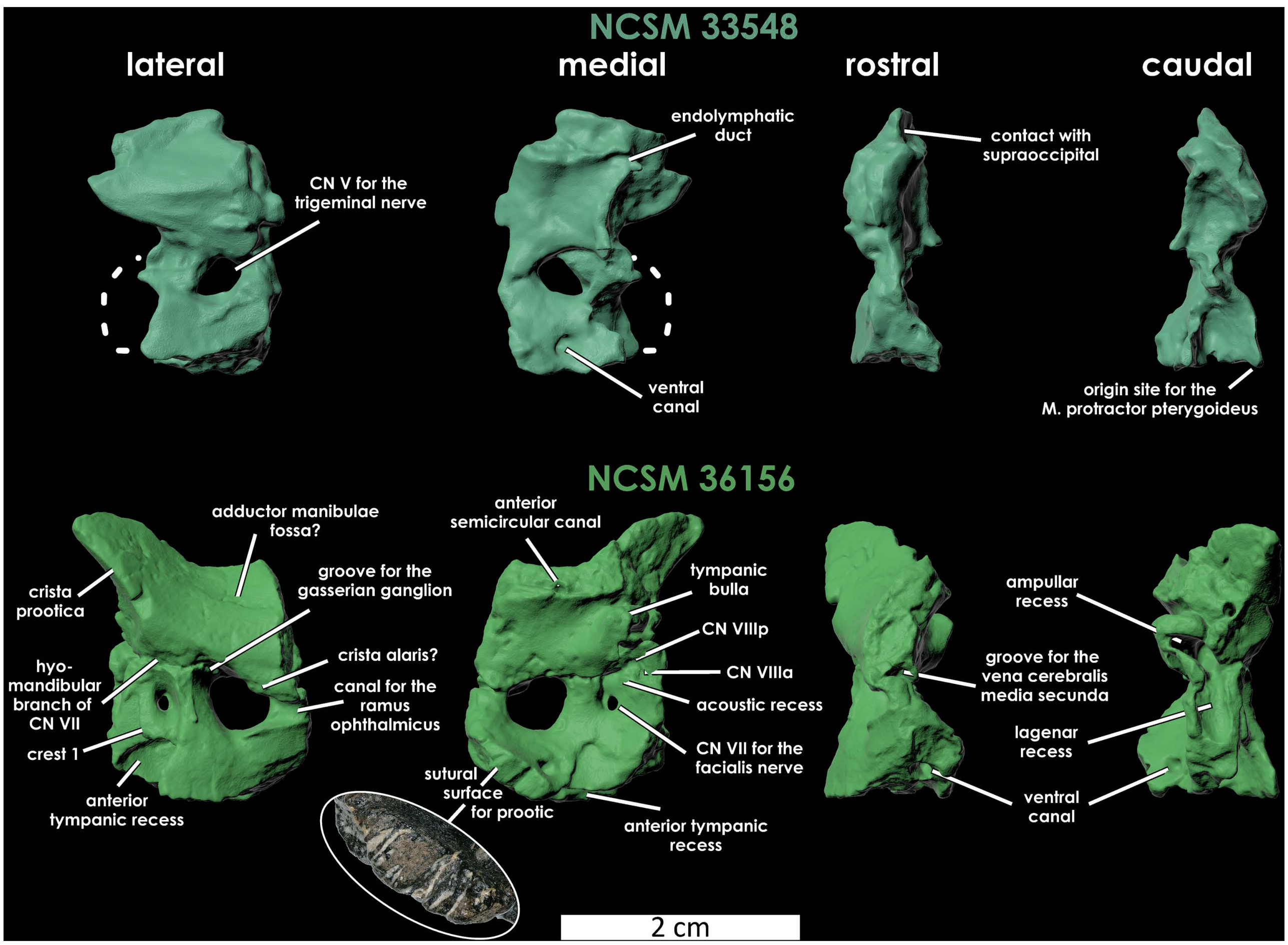

Fig 19. Prootic of *Fona herzogae*. Dashed lines represent missing bone.

https://doi.org/10.1371/journal.pone.0353169.g019

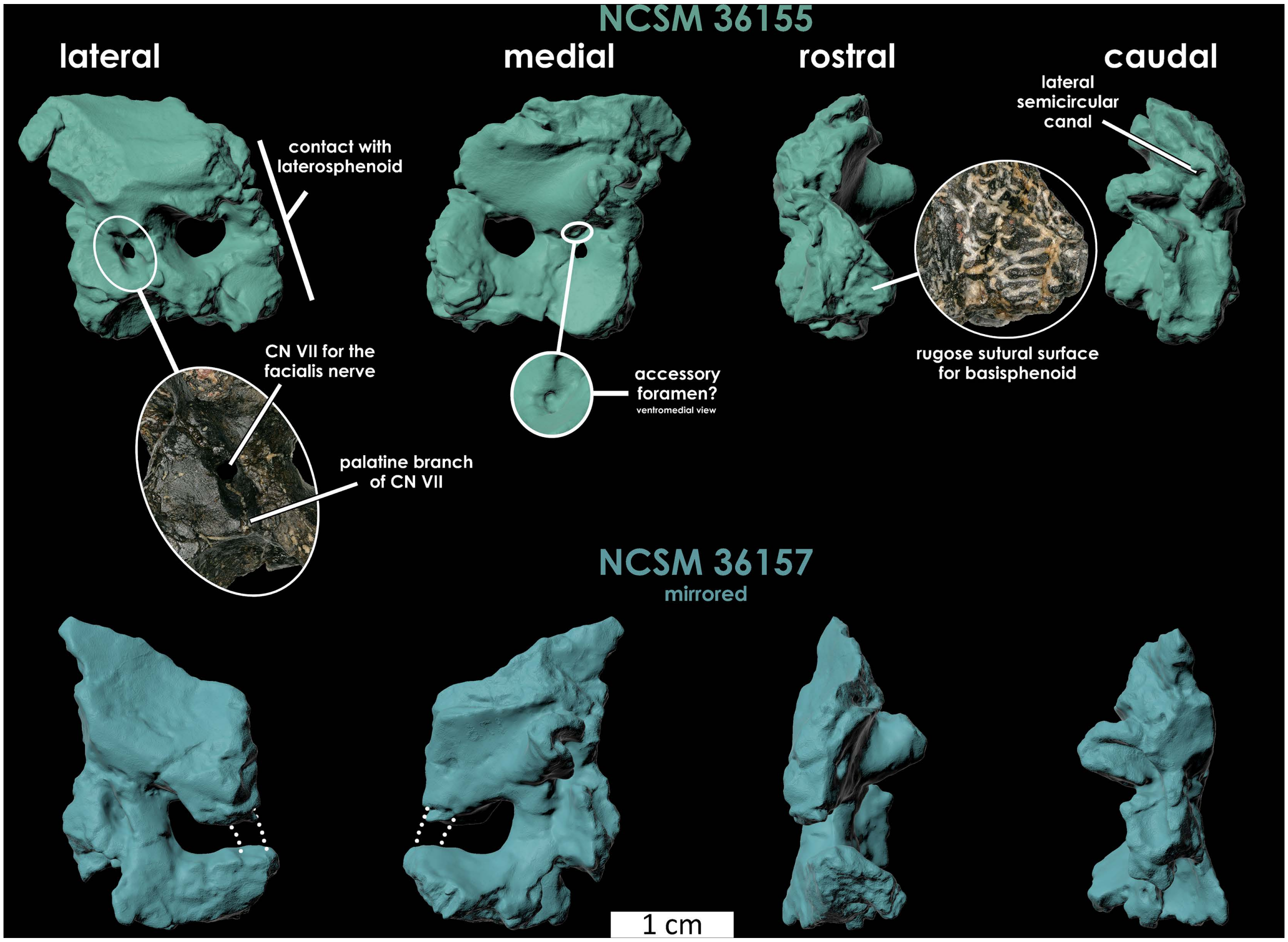

Fig 20. Prootic of *Fona herzogae*.

https://doi.org/10.1371/journal.pone.0353169.g020

Crista prootica: In lateral view, the ventrolateral margin of the caudal process forms a rounded ridge called the crista prootica, also termed the otosphenoidal crest [87]. This ridge begins at the paraoccipital process and becomes more pronounced as it continues onto the lateral surface of the caudal process, where it serves as the attachment site for the *M. levator pterygoideus* [39]. The crista prootica follows an arcing rostroventral path, creating the division between CN VII and CN VII, and continues ventrally on its way to the basipterygoid, as in *Dysalotosaurus* [67].

Lateral surface: The lateral surface of the prootic is relatively flat compared to the dorsoventrally concave medial surface.

CN V: In lateral and medial views, there are two foramina (CN V and CN VII) located side by side and separated by the ventral descent of the crista prootica. Both foramina pierce mediolaterally through the prootic. The larger of the two (CN V) houses the trigeminal nerve and the middle cerebral vein [67]. It is located rostral to the smaller CN VII and is completely enclosed within the prootic. All prootics of *Fona* bear fully enclosed CN V, in contrast to *Th. assiniboiensis* [60] and *Hypsilophodon* [NHMUK R 2477; 69], in which the laterosphenoid participates in the rostral margin of the foramen. In lateral view there are two medially depressed canals associated with CN V. The larger canal, which indicates the position of the gasserian ganglion [88,89] (termed CN V3 in the sauropodomorph *Massospondylus carinatus* [90]), begins broadly across the caudodorsal margin of CN V, projects caudodorsally, and becomes less medially deep as its dorsal and ventral

margins converge. The smaller canal, which housed the ramus ophthalmicus [69], extends rostrally from the rostral margin of CN V and likely continued onto the laterosphenoid [69], whereas in *Th. neglectus,* this canal extends rostrally from the rostrodorsal corner of CN V [18].

Crista alaris: There is a narrow ledge that appears to emerge as a bifurcation of the crista prootica before its descent between CN VII and CN V. This ledge runs rostrally and slightly ventrally, forming the dorsal margins of CN V and the ramus ophthalmicus canal. In *Th. neglectus,* this ledge may correspond to the crista alaris [18,85,88]. Sobral et al. [67] states that the rostral portion of the crista alaris in *Dysalotosaurus* shows muscle attachment markings for an origin site of the *M. pseudotemporalis superficialis*, a condition that does not appear to be present in *Fona* or *Th. neglectus*. The crista alaris may have also served as the attachment site of the prootic membrane, a condition present in extant squamates [85,88]. Additionally, there is a prominent rostrocaudally oriented groove located dorsal to the crista alaris on NCSM 36155 that is reduced on the others.

Terminological inconsistencies: There seem to be some inconsistencies between the terminological definitions and statements of homology regarding various crests across the surface of the prootic. For example, Sobral et al. [67] and Thomas [4] consider the crista prootica of *Dysalotosaurus* and *Te. tilletti* to be rostrally continuous across the lateral surface of the prootic, contacting the caudal aspect of the laterosphenoid dorsal to CN V. Likewise, Thomas [4] refers to the crest that descends ventrally between CN V and CN VII as the crista tuberalis. In contrast, Sampson and Witmer [87] and Sobral and Müller [88] consider the crista prootica/otosphenoidal crest to descend rostroventrally between CN V and CN VII on its way to the basipterygoid in *Mesosuchus* and *Majungasaurus,* and Sobral and Müller [88] refer to the prominent crest located caudodorsal to CN V as the crista alaris. We follow the later interpretations.

CN VII: The smaller foramen (CN VII) houses the facialis nerve and is located directly caudal to CN V. In lateral view, its circular margin does not begin as a sharp drop-off but rather lies within a deep elliptical recess as in *Dysalotosaurus* [89]. In contrast to *Dysalotosaurus,* the ventral margin of CN VII does not form the prominent channel that carries the palatine branch of CN VII (also termed the ramus palatines [69]). The dorsal margin of CN VII on prootic NCSM 36155 forms a prominent caudodorsally oriented channel that carries the hyomandibular branch of the facial nerve as in *Dysalotosaurus* [67] and the archosaur *Mesosuchus browni* [88].

Crest 1: In lateral view of NCSM 36156 and 36157, there is a subtle dorsoventrally oriented crest located caudodorsal to the facial nerve, which may correspond to crest 1 in the sauropodomorph *Massospondylus carinatus* [90].

Lateroventral surface: In lateral view, the lateral surface of the prootic, rostral to the crista prootica and ventral to CN V, is a flat surface. The rostral portion of this region is referred to as the anterior inferior process in the archosauriform *Euparkeria capensis* [89] and the archosauromorph *Mesosuchus browni* [88]. The surface of this region is relatively smooth and lacks the extensive rugosities reported in *Dryosaurus* and *Tenontosaurus* [69]. The caudal aspect of this surface forms an acute angle with the curve of the crista prootica, also forming a mediolaterally thin sheet that overlaps the anterior tympanic recess and additionally serves as the origin for the superior part of the *M. protractor pterygoideus* [67,69].

Medial surface: In medial view, the medial surface of the prootic is smooth and slightly dorsoventrally concave. The medial opening of CN VII is housed within a shallow acoustic recess (termed the fossa acustica interna in the sauropodomorph *Panphagia protos* [86]) located ventral to the medially extruded tympanic bulla (termed the otic capsule in the sauropodomorph *Pantydraco caducus* [91]). This acoustic recess is present in *Dysalotosaurus* and *Th. neglectus* [18,67].

CN VIIIa and VIIIp: The exact quantity and location of the foramina for the acoustic nerve (CN VIII) are somewhat difficult to diagnose due to poor preservation in the caudodorsal portion of the acoustic recess, however, these canals do seem to be present and similarly arranged to those in *Dysalotosaurus* and *Th. neglectus* [18,67]. NCSM 36155 is more damaged in this region and has two apertures that pierce through the caudodorsal wall of the acoustic recess, however, under the microscope, no clear canal wall could be identified. The acoustic recess of NCSM 36156 is better preserved, and when viewed under the microscope, it is clear that the walls of its two openings represent original bone surface. The

more rostrally located opening likely represents the anterior ramus of the acoustic (or auditory) nerve (CN VIIIa) (termed the vestibulocochlear in Brown et al. [60]), this foramen travels dorsolaterally through the ventral wall of the tympanic bulla, opening into the ampullar recess (termed the utricular recess in Boyd [18], the cavity of the anterior vestibule in Brown et al. [60], the vestibular recess in [86], and the otic cavity in [91]). The more caudally located opening likely represents the posterior ramus of the acoustic nerve (CN VIIIp) and travels caudolaterally, opening into the caudal aspect of the lagenar recess (termed the sacculus in Chapelle and Choiniere [90]).

Accessory foramen: In medial view of NCSM 36155, there is a foramen located directly dorsal to CN VII. This foramen has not been described before and appears to be absent in the other prootica that preserve this region.

Ventral tympanic crest: In medial view, there is a caudally directed, laterally oriented crest located at the ventral margin of the tympanic bulla. On NCSM 36156, there is a large hole located directly ventral to this crest on the tympanic bulla, but this is likely due to damage.

Vena cerebralis media secunda: In medial view, there is a rostrocaudally oriented groove at the rostrodorsal margin of CN V, which likely housed the vena cerebralis media secunda [92], as in *Dysalotosaurus* and *Th. neglectus* [18,67]. When seen in rostral view, this groove is located directly opposite, and slightly dorsal to, the canal for the ramus ophthalmicus.

Endolymphatic duct: At the caudodorsal region of the prootic in medial view, there is a shallow groove located dorsal to the tympanic bulla, called the endolymphatic duct. This feature has been previously misidentified as the fossa subarcuata [18,69] and the vena cerebralis media [21]. This terminological inconsistency was clarified by Sobral et al. [67], who took into consideration the morphology of the osseous labyrinth to determine that the structure was more likely to be the channel for the endolymphatic duct.

Dorsum sellae: In medial view, the rostroventral corner of the prootic forms a medially directed prominence, matching the medial height of the tympanic bulla. This prominence may represent the dorsum sellae, which emerges from the clinoid process in the sauropodomorph *Panphagia protos* [86]. However, this structure is far more robust than the thin, fan-shaped lamina in *Panphagia protos* and, therefore, may not be homologous.

Ventral canal: There is a canal located at the rostroventral region of the prootic, which pierces through the prootic as is travels caudally, and somewhat medially, through the dorsum sellae. When viewed in rostral view, the rostral opening of this canal is positioned along the contact with the basisphenoid. This canal has not been described on the prootic before and may represent a continuation of the carotid canal of the basisphenoid (for hadrosaurid examples, see figure 2A of [93] for *Kerberosaurus manakini* and figure 11 D of [94] for *Kundurosaurus nagornyi*).

Caudal surface: The caudal surface of the prootic is characterized by a series of deeply concave recesses and cavities. The ampullar recess (see above for the plethora of terminological definitions previously used regarding this feature) is a deeply inset cavity, excavating halfway into the rostral length of the prootic. In caudal view, the opening for this cavity forms a semi-elliptical shape, with its long axis oriented dorsomedially. Ventral to the ampullar recess is the shallower lagenar recess. It should be noted that the homology of this depression to other taxa is unclear due to possible terminological inconsistencies. Nonetheless, if it represents the same feature referred to as the lagenar recess in *Dysalotosaurus*, it would have housed the perilymphatic cistern and the lagena [67].

Semicircular canal: In caudal view, there is a small circular hole located between the ampullar recess and the caudal process that represents the lateral semicircular canal and continues caudally on to the opisthotic. The rostral path this canal takes through the prootic is unknown but seems similar to the morphology in *Dysalotosaurus,* likely joining with the anterior semicircular canal inside the ampullar recess [67]. The anterior semicircular canal can be seen in medial view. It borders the dorsal margin and continues into the supraoccipital.

Anterior tympanic recess: In caudal and ventral views, at the caudoventral region of the prootic, there is a deeply concave recess that occupies over half the rostrocaudal length of the ventral surface, with a dorsally directed depth that nearly reaches the ventral margin of CN VII. This recess is referred to as the anterior tympanic recess in the

sauropodomorph *Panphagia protos* [86] and is also present on a possible isolated stegosaurian prootic [95]. This recess is laterally overlain by a thin sheet of bone formed by the ventral descent of the crista prootica and is broken on NCSM 36155.

Basisphenoid/Parasphenoid

Preservation: A well-preserved, fully fused basisphenoid/parasphenoid/basioccipital is preserved from NCSM 33548. Additionally, two partially preserved basisphenoid/parasphenoids (NCSM 36128 & 36129) are preserved from the Mini Troll locality, both lacking a fusion with a basioccipital.

General shape: The basisphenoid/parasphenoid is a complex quadradiate bone (Fig 21).

Articulations: It forms the rostroventral portion of the braincase and connects to the pterygoid rostrally, the prootic dorsally, and the basioccipital caudally.

Ventral view: In dorsoventral view, the basisphenoid has a transversely constricted hourglass shape. Excluding the cutriform process, it is equivalent in rostrocaudal length to the basioccipital, in contrast to *Th. neglectus,* which is shorter than the basioccipital [18].

Basipterygoid process: In lateral view, the basipterygoid processes, which fit into concave facets on the pterygoids, emerge from the rostral corners of the main body and are directed rostroventrally, relative to the long axis of the ventral margin, as in *Lesothosaurus, Heterodontosaurus, Haya,* and *Th. neglectus* [3,18,25,36]. This contrasts with the more ventrally directed condition in *Zephyrosaurus, Dysalotosaurus,* and *Hypsilophodon* [7,21,67].

The basipterygoid processes are relatively longer than those in *Haya* [25] but not as elongate as *Te. tilletti* [4]. On NCSM 36129, only the left process is preserved. It does not taper along its length, and its distal tip ends in a flat surface; however, it may be incomplete. The processes of NCSM 36128 and 33548 are relatively longer and have rounded ends. The basipterygoid processes are ovoid in cross section, with the rostral margin tapering to a rounded edge, as occurs in *Th. neglectus* [18], but in contrast to *Oryctodromeus* (MOR 1642), which taper to a slightly broader but much thinner edge.

In rostral view, the angle formed between the medial surfaces of the processes' distal ends and the ventral surface of the basisphenoids' main body ranges from 60° – 70°. This is in contrast to *Th. neglectus* (~80°), *Th. assiniboiensis* (~88°), *Hypsilophodon* (~80°), *Lesothosaurus* (~74° –~ 103°), and *Zephyrosaurus* (~59°). The processes of NCSM 33548 are directed ventrally at their origin before becoming laterally kinked at their mid-length.

The lateral surface of the left process of NCSM 33548 bears a prominent fossa that is absent on the right processes as well as the other two *Fona* basisphenoids. These fossae are present on both processes of the fused basisphenoid/basioccipital of *Oryctodromeus* (MOR 1642). Near the distal end of the processes, the lateral surface forms a broad, slightly convex expansion that is present among a number of SBEDOs.

The lateral surface of the basipterygoid process of *Oryctodromeus* (MOR 1642) bears a distinct ridge with well-defined margins that runs diagonally from the caudoventral end of the processes to the rostral margin at mid-length. In *Zephyrosaurus* (MCZ 4392), this ridge is less pronounced and broadly rounded. Additionally, the medial surfaces of *Zephyrosaurus* bear a distinct ridge that mirrors the orientation of the lateral ridge, giving the process a spade-shaped profile in rostral view. This medial ridge is absent in *Fona* and *Oryctodromeus.*

Preotic pendants: The preotic pendants emerge as thin, sheet-like projections from the caudodorsal margin of the basipterygoid processes and likely represent the ventral continuation of the otosphenoidal crest of the prootic. They drape caudally over the lateral surface of the basisphenoid body to form deep, caudoventrally facing basipterygoid recesses. As in *Th. neglectus,* the lateral surfaces of the rostral processes and preotic pendants are slightly concave [18], likely in order to accommodate the vena capitis lateralis, as occurs in *Dysalotosaurus* [67].

Parasphenoid: On NCSM 33548 and 36129, there is no evidence of a demarcation between the parasphenoid rostrally and the basisphenoid caudally. NCSM 36128 does show a possible suture boundary rostrodorsally. However, this is more likely the result of damage and cracking, which is prevalent across this specimen. The dorsal margin of the

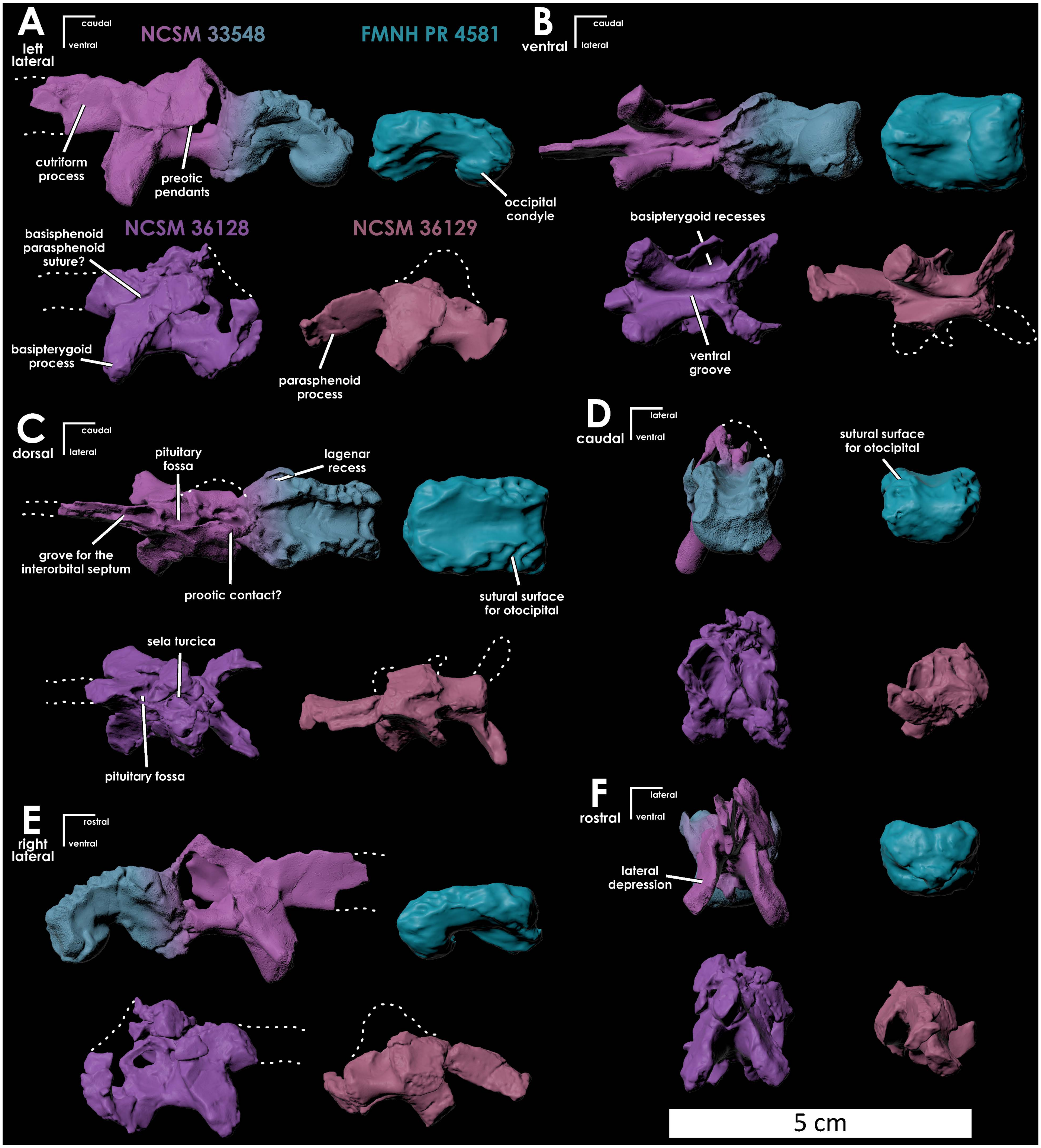

Fig 21. Basisphenoid, parasphenoid, and basioccipital of *Fona herzogae*. Dashed lines represent missing bone.

https://doi.org/10.1371/journal.pone.0353169.g021

parasphenoid is level with the basisphenoid, in contrast to *Hypsilophodon* and *Dysalotosaurus,* in which the parasphenoid emerges more ventrally [47,67].

Cultriform process: The rostral portion of the cultriform process is missing on NCSM 33548, yet the remaining caudal portion is well preserved. Only the base of the process is preserved on NCSM 36128. Although a substantial portion is preserved on NCSM 36129, it is partially taphonomically deformed. The cultriform process is triangular in cross section with a wider dorsal margin. The dorsal surface bears a distinct groove that runs down the length. This groove housed the cartilaginous interorbital septum and is separated from the pituitary fossa, as occurs in *Lesothosaurus* and *Haya* [3,25], and in contrast to *Th. neglectus* in which a foramen connects this groove with the sella turcica [18].

Although the ventral margin of the process does taper to a rounded edge, the distinct ventrally descending keel that is present in *Haya* [25]*, Th. neglectus* [18], and possibly *Orodromeus* (MOR 1136) and *Zephyrosaurus* (MCZ 4392), seem to be absent, at least caudally. *Lesothosaurus*, *Dysalotosaurus* [3,67], and *Oryctodromeus* (MOR 1642) also lack a ventral keel.

Caudal process: The mediolaterally thin caudal processes formed the contact with the basioccipital and curve ventrolaterally and dorsally from their emergence at the caudolateral corners of the main body. They are less broad and more dorsally inflected than *Th. neglectus* [18], and are much longer than *Haya* [25].

Dorsal surface: In dorsal view, the pituitary fossa has a triangular shape. Caudal to the pituitary fossa, the opening for the sella turcica is only preserved, albeit poorly, on NCSM 36128. The poor preservation prevents identification of the presence or absence of the foramen for the abducens nerve (CN VI) that occurs in *Th. neglectus* [18]. Caudal to the sella turcica, the main body of the basisphenoid bears a somewhat spade-shaped cross-section and forms a thin sheet that rises dorsally. This sheet is only preserved on NCSM 36128 and is perforated, possibly by the carotid foramen.

Ventral view: In ventral view, there is a deep midline groove that begins at the caudal contact with the basioccipital and runs rostrally, bifurcating medial to the base of the basipterygoid processes, directly caudal to the emergence of the cultriform process. This groove varies extensively in width and prominence between all three individuals of *Fona,* being extremely narrow with distinct, parallel lateral margins on NCSM 36129, wider yet well-defined across the parallel lateral margins on NCSM 33548 (a condition shared with *Zephyrosaurus* (MCZ 4392) and *Oryctodromeus* (MOR 1642)), and widest with poorly defined, slightly rostrally diverging lateral margins on NCSM 33548, a condition shared by *Haya* (MPC-D 100/2017) [25]. A similar extent of variability seems to occur in other SBEDOs. For example, the ventral groove of *Lesothosaurus* from the braincase block of NHMUK PV RU B17 [3] completely lacks defined margins, exhibiting a broad, gently concave surface, whereas a second basisphenoid/parasphenoid from the palate block shows a groove closer in morphology to *Fona* NCSM 33548. In contrast, the two species of *Thescelosaurus* are consistent in that they lack any ventral concavity altogether, instead being broadly convex across the ventral surface [18,60]. In *Dysalotosaurus*, *Hypsilophodon,* and possibly *Orodromeus* (MOR 1136), there is no midline groove. Rather, these taxa possess a deep pit between the basipterygoid processes that represents an embryonic hypophyseal fenestra [7,67].

Basioccipital

Preservation: *Fona* is represented by a well-preserved basioccipital fused to the basisphenoid/parasphenoid (NCSM 33548) and another that is well-preserved and unfused (FMNH PR 4581).

General shape: The basioccipital is a bluntly rounded hourglass/saddle-shaped bone (Fig 21).

Articulations: It forms the caudoventral aspect of the braincase and articulates with the otoccipitals dorsally, the basisphenoid rostrally, and the atlas of the axial skeleton caudally.

Lateral surface: In lateral view, the ventral margin of the basioccipital is deeply concave, and in dorsal view, the lateral margins are slightly medially constricted. In lateral view of NCSM 33548, the floor of the foramen magnum and the dorsal surface of the basioccipital, which is heavily excavated to form the suture with the otoccipitals, is arched dorsally ~25° from the long axis of the basisphenoid (with the vertex placed at the caudal end of the occipital condyle), a condition

similar to *Th. neglectus* and *Th. assiniboiensis* [18,60]. In contrast, the basioccipital of *Oryctodromeus* (MOR 1642) is flat and oriented parallel with the basisphenoid, a condition shared with *Zephyrosaurus* and *Hypsilophodon* (NHMUK R2477). However, the other basisphenoid of *Oryctodromeus* (MOR 1636) is tilted ventrally by as much as ~50°, a condition shared with the early diverging iguanodontian *Iani* [1], suggesting the orientation of the basioccipital and basisphenoid may be extensively intraspecifically or ontogenetically variable.

Dorsal surface: There is no small midline ridge on the dorsal surface of the foramen magnum near the basisphenoid contact, in contrast to *Zephyrosaurus* (MCZ 4392 & YPM 56695), *Oryctodromeus* (MOR 1642), *Th. neglectus* [18] and possibly *Th. assiniboiensis* [60]. This midline ridge is also absent in *Oryctodromeus* (MOR 1636), suggesting that it may be intraspecifically variable.

Ventral surface: In ventral view, the basioccipital is equivalent in rostrocaudal length to the basisphenoid (excluding the cultriform process). This is in contrast to *Jeholosaurus* [22], *Thescelosaurus,* and a number of dryosaurids and iguanodontians [18,60], in which the basioccipital exceeds the length of the basisphenoid, or *Orodromeus* (MOR 1136) and *Lesothosaurus* [3], in which the basisphenoid is longer than the basioccipital. In ventral view, the shape of the occipital condyle's rostral margin is variable, being mediolaterally flat on FMNH PR 4581, whereas on NCSM 33548, it forms a prominent, rostrally directed curve. A similar range of variation is present in *Zephyrosaurus,* suggesting this feature may be intraspecifically variable. *Oryctodromeus* (MOR 1642) bears two bilateral foramina near the craniodorsal corner of the occipital condyle, which are absent in *Oryctodromeus* MOR 1636 as well as *Fona.*

Rostrally, the basioccipital forms a "V" shaped profile to insert into the basisphenoid as in *Th. neglectus* [18] and *Hypsilophodon* (NHMUK R2477), but in contrast to the "W" shaped profile in *Zephyrosaurus, Orodromeus* (MOR 1136), and *Lesothosaurus* [3].

The rostral half of the basioccipital of NCSM 33548 is characterized by a distinct ventral midline ridge or keel. This keel is prominent and descends below the ventral margin of the basisphenoid, a condition shared with *Zephyrosaurus* (YPM 56695) and possibly *Oryctodromeus* (MOR 1642; the full extent is unknown due to damage). In contrast, this midline keel is less prominent in FMNH PR 4581 and *Zephyrosaurus* (MCZ 4392), more closely resembling the condition in *Thescelosaurus* [18,60], *Hypsilophodon* (NHMUK R2477), *Orodromeus* (MOR 1136), and *Lesothosaurus* [3], suggesting it may be intraspecifically variable. This keel is not laterally bordered by two small knobs in contrast to *Th. neglectus* [18].

Oryctodromeus was originally described as having a steeply sided ventral 'box' [83] below the occipital condyle. This condition is shared by NCSM 33548, in which the ventrolateral surfaces of the basioccipital form a subtle shelf at the element's mid-length. However, this box is absent on the other *Oryctodromeus* basioccipital (MOR 1642) as well as one of *Fona* (FMNH PR 4581), suggesting that this feature is intraspecifically variable.

Pterygoid

Preservation: A single well-preserved left pterygoid is preserved from the Mini Troll locality (NCSM 36158).

General shape: The pterygoid is a complex triradiate bone characterized by two short rami, a larger sheet-like ramus, and a medial projection (Fig 22).

Articulations: It forms the caudal-most element of the palate and connects to the quadrate caudally, the ectopterygoid ventrolaterally, and the palatine rostrally.

Quadrateramus: The quadrate ramus, also referred to as the alar process, is a large sheet-shaped process that makes up approximately 50% of the pterygoid length. It is mediolaterally thin and dorsoventrally wide, but not as much as *Th. neglectus* and *Lesothosaurus* [3,18]. It broadens slightly from the main body of the pterygoid as it projects caudally to overlap the medial surface of the quadrate's pterygoid wing. The lateral surface is slightly convex and shows faint rugosities for articulation with the quadrate, as in *Th. assiniboiensis* and *Hypsilophodon* [7,60]. There is no sign of the lateral pterygoid ridge across the lateral surface, as is present in *Th. neglectus* [18].

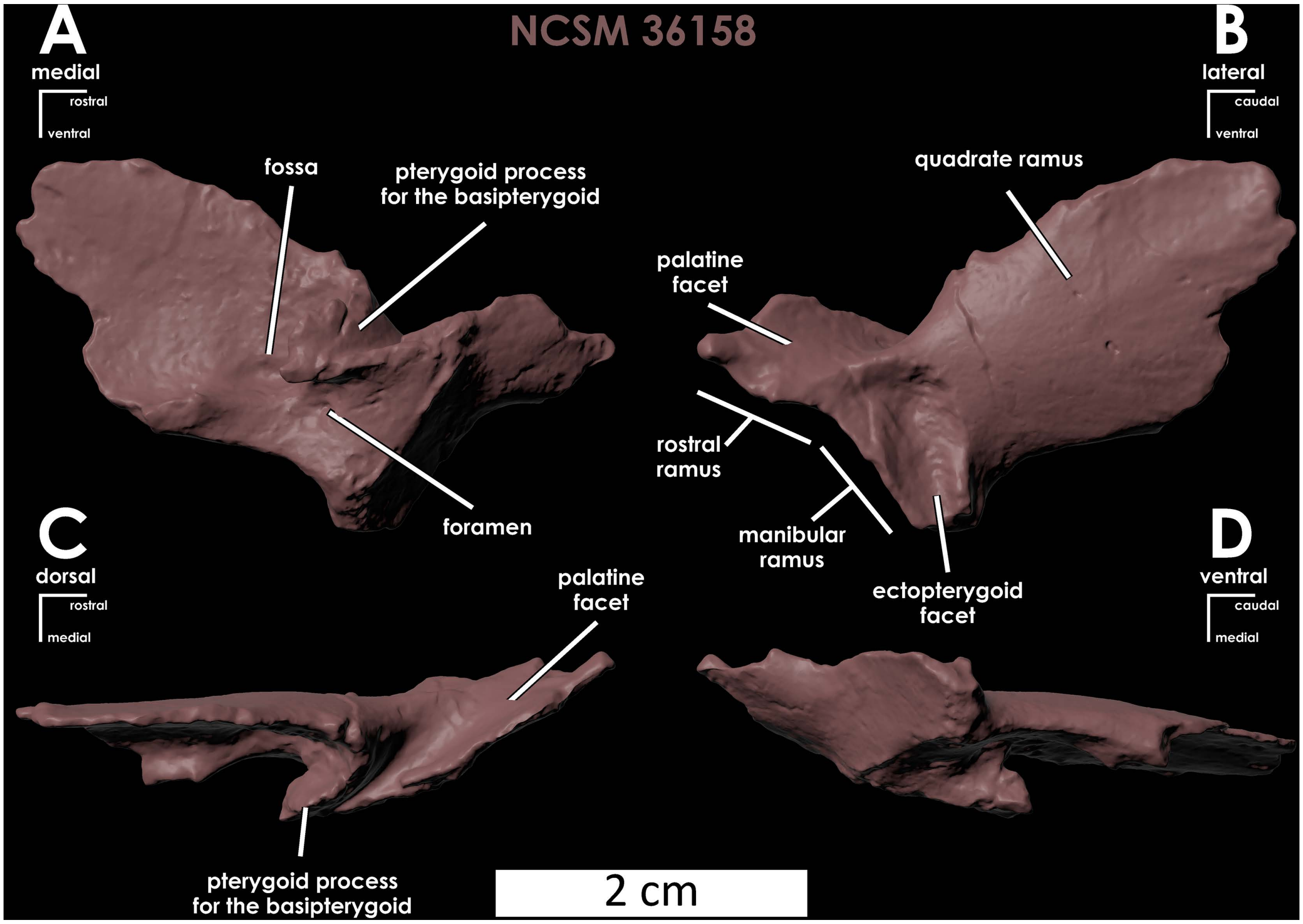

Fig 22. Pterygoid of *Fona herzogae*.

https://doi.org/10.1371/journal.pone.0353169.g022

Pterygoid process: The medial surface is slightly dorsoventrally concave. At the base of the quadrate ramus near the dorsal margin, there is a distinct thumb-like projection called the pterygoid process. It has a "U" shaped cross-section and projects caudoventrally. The lateral surface of this process accepts the basipterygoid process of the basisphenoid, whereas the medial surface possibly forms a contact with the contralateral pterygoid. In *Lesothosaurus,* this process has a more caudal orientation [3].

There is a rostrodorsally directed, medially expanded ridge at the base of the quadrate ramus along the ventral margin, as occurs in *Lesothosaurus*, *Jeholosaurus,* and *Changchunsaurus* [3,19,22]. It is more prominent ventrally, attenuating rostrodorsally as it merges with the base of the pterygoid process. Dorsal to the junction between this ridge and the pterygoid process is a subtle concave fossa on the medial surface of the ramus. Additionally, a small foramen occurs directly ventral to the pterygoid process.

Mandibular ramus: The mandibular ramus, also referred to as the pterygoid flange, extends ventrally from the base of the quadrate ramus. It is a short process, and its maximum length is difficult to determine due to damage. The lateral surface of the ramus is rostrocaudally concave and forms a thin lamina of bone that extends rostrodorsally toward the rostral ramus, terminating at a thin rostral ridge that demarcates the boundary between the two rami. This concave region accepts the medial head of the ectopterygoid.

Rostralramus The rostral ramus extends rostrolaterally from the main body of the pterygoid and possibly contacts the vomer medially, as in *Lesothosaurus* [3], although its full length is not preserved. It is a mediolaterally thin process with a flat lateral surface that merges evenly with the mandibular ramus. Its lateral surface facilitates articulation for the palatine.

Palatine

Preservation: Two partially preserved left palatines are represented from the Mini Troll locality (NCSM 36145) and the Karmic locality (NCSM 33548).

General shape: The palatine is a flat triradiate bone characterized by two short projections and a larger sheet-like ramus (Fig 23).

Articulations: It connects to the pterygoid caudally and the maxilla rostrolaterally, likely the contralateral palatine medially, and possibly the ectopterygoid caudolaterally.

In dorsal view, the palatine is an Ogham reversed feather mark "᚜" shaped bone. It is dorsoventrally flattened with a rostrocaudal length nearly double its mediolateral width. The palatine of *Haya* [25], *Iani* [1], and *Lesothosaurus* [3] are also longer than they are wide, in contrast to *Th. neglectus,* which has a more equivalent length-to-width ratio [18]. It forms a thin sheet across its caudal aspect, which overlaps a process on the pterygoid. The ventral surface of this lamina is smooth and flat, lacking the ventral prong seen in *Th. neglectus* (personal observation of left palatine NCSM 15728). The rostrolaterally directed process is the most robust region of the element, forming a thickened and caudally hooked sutural surface that likely articulated with the maxilla and possibly the lacrimal and jugal. In contrast, the rostromedial process is much thinner dorsoventrally, likely incomplete on both specimens, and may have contacted the contralateral palatine.

The ventral surface is characterized by a narrow, rostrocaudally oriented groove formed by two thin lamina running in parallel and located along the midline of the element. This groove is bordered laterally and medially by two broad and weakly concave surfaces. This is in contrast to the much larger, laterally positioned grooves that serve to embrace the maxilla in *Iani* and *Th. neglectus* [1,18].

A prominent caudolaterally projected process was noted in *Iani*, which emerges along the curved caudolateral margin between the pterygoid and maxillary process [1]. Reprocessed CT scans of *Th. neglectus* reveals it to be a shared feature (personal observation). It appears to be absent in *Fona.*

Ectopterygoid

Preservation: A single well-preserved right ectopterygoid is preserved from the Mini Troll locality.

General shape: The ectopterygoid is a sideways "L" shaped bone characterized by a central shaft that turns into a larger medial head (Fig 24).

Articulations: It connects to the pterygoid medially, the jugal laterally, and possibly the palatine and maxilla rostrally.

Medial head: The medial third of the ectopterygoid forms a main body that is broader dorsally and tapers ventrally, with a mediolaterally concave caudal surface and a flat medial surface that facilitates articulation with a concave groove on the mandibular ramus of the pterygoid. The rostromedial corner does not have the postpalatine fenestra present in *Th. neglectus* [18].

Lateral shaft: The rostrolateral two-thirds of the ectopterygoid form a shaft that emerges from the main body. Its lateral surface is strongly dorsoventrally convex, whereas its medial surface is flat and bears a deep groove that likely accepts the palatine; this contact is present in *Th. neglectus* [18], *Haya* [25] and *Lesothosaurus* [3], but is absent in *Changchunsaurus* [19] and *Hypsilophodon* [7]. Its dorsal and ventral margins converge gradually across its length, whereas its lateral and medial margins run in parallel until the tip, diverging from each other as the shaft twists slightly about its axis. This forms an ax head-shaped lateral edge that fits into a groove on the medial surface of the jugal. The shaft is not bowed along its length, nor is it as dorsally oriented, in contrast to *Th. neglectus* and *Lesothosaurus* [3,18]. The overall morphology of the ectopterygoid is similar to that of *Talenkauen* [37], although it lacks the prominent dorsal lobe. There is a small

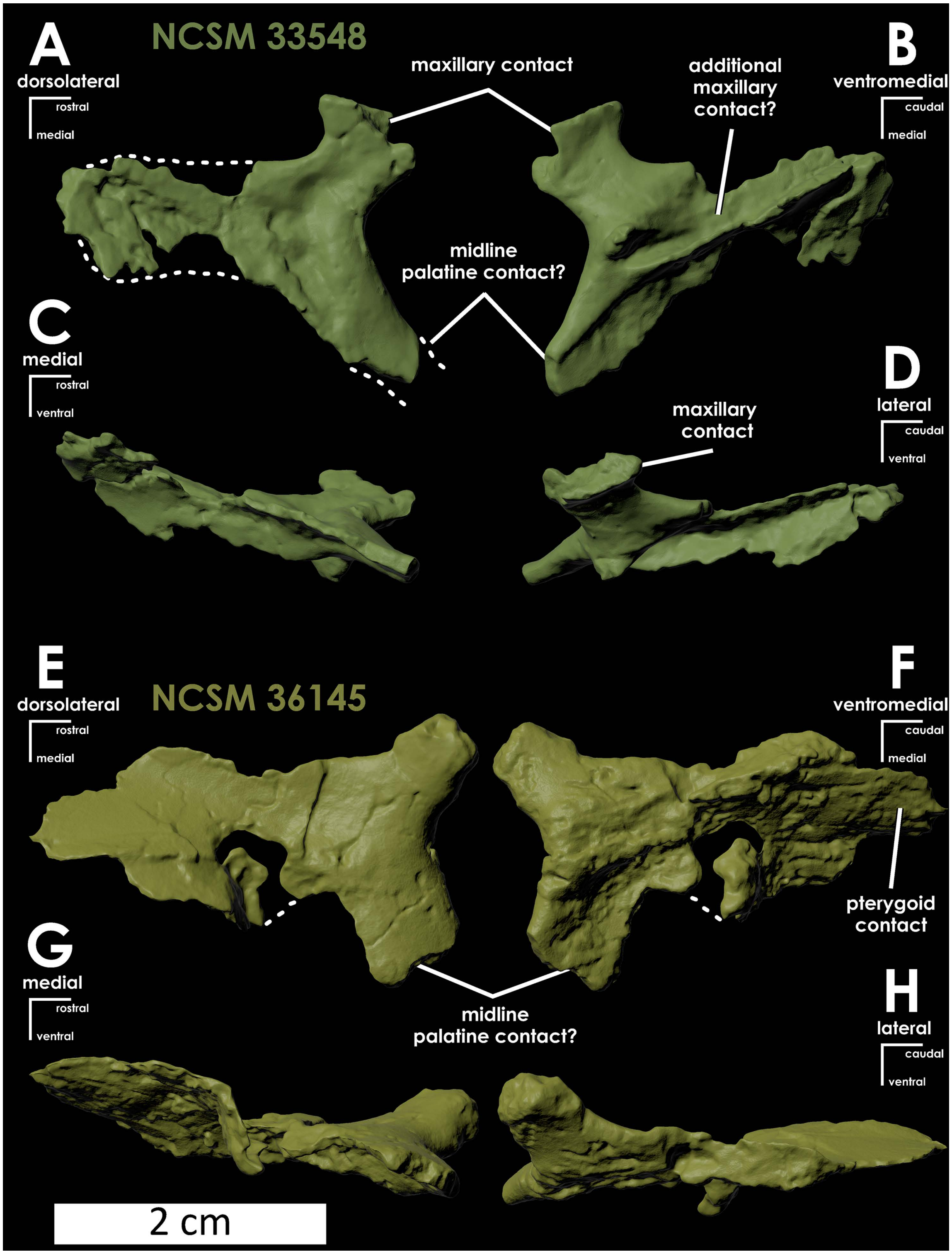

Fig 23. Palatine of *Fona herzogae*. Dashed lines represent missing bone.

https://doi.org/10.1371/journal.pone.0353169.g023

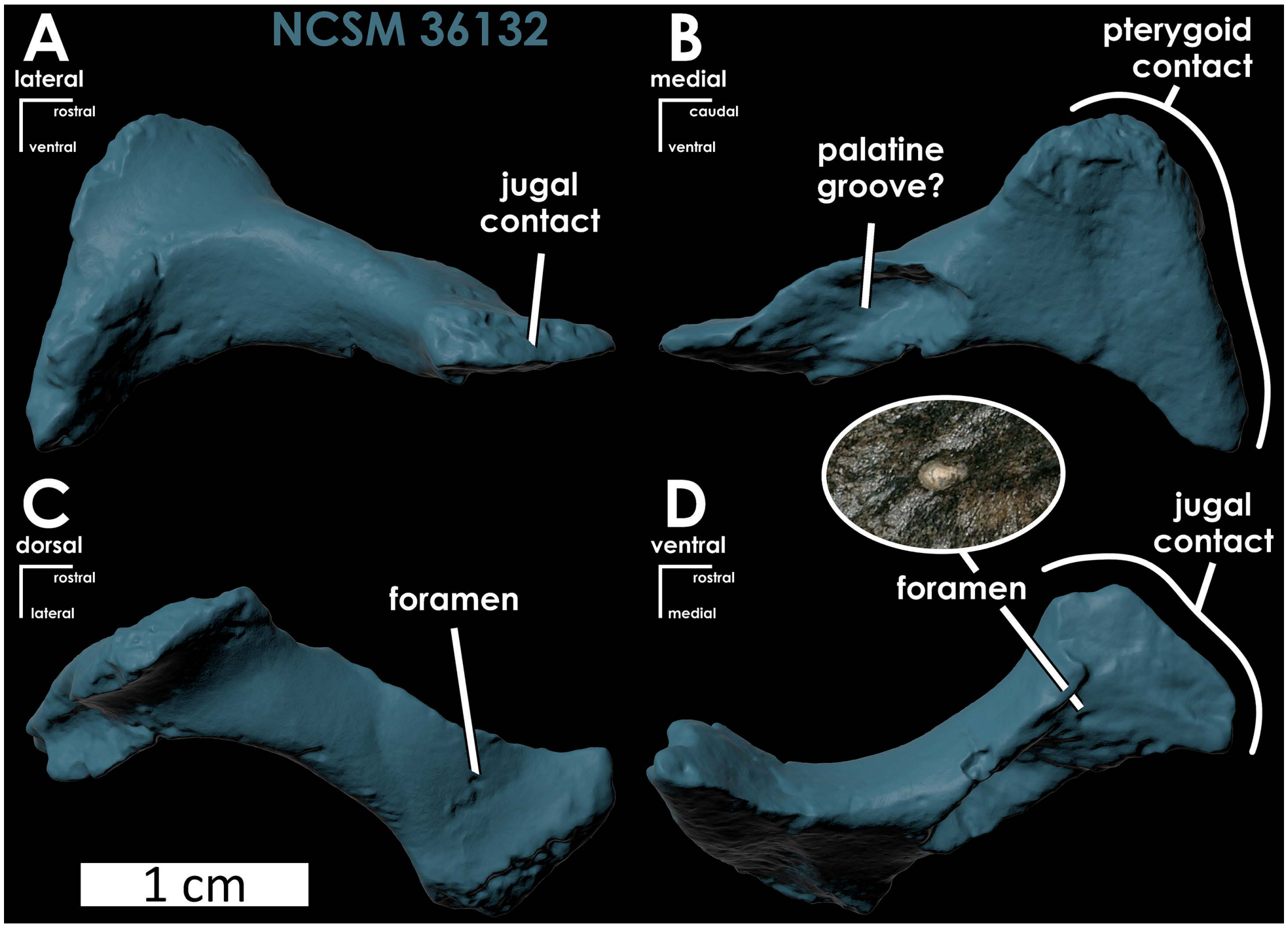

Fig 24. Ectopterygoid of *Fona herzogae.*

https://doi.org/10.1371/journal.pone.0353169.g024

foramen located lateral to the palatine groove and another on the dorsal surface near the jugal contact, possibly representing the same canal structure.

Dentary

Preservation: A well-preserved complete left dentary is represented from the Karmic locality (NCSM 33548) (Fig 25). A well-preserved, complete left (NCSM 36131) (Fig 26) and a moderately well-preserved, partially complete right (NCSM 36130) dentary (Fig 27) are represented from the Mini Troll locality. A fourth poorly preserved dentary (S1.20 File) is preserved from the Manolo locality (FMNH PR 4581).

General shape: The dentary is a rostrocaudally elongate, laterally narrow bone characterized by a single row of teeth running along the dorsal margin.

Articulations: The dentary is the largest element of the mandible and is overlapped by the splenial and coronoid caudomedially. It connects to the predentary rostrally and the angular, prearticular, and surangular caudally.

Medial Surface: The medial surface of the dentary is slightly convex rostrocaudally and dorsoventrally. The medial surface becomes slightly dorsoventrally concave near the rostral fourth of the dentary, as the ventral margins of the rostral dentary twist to form the medially expanded medial process.

Connection with predentary: As in *Th. neglectus,* the medial process is positioned nearly level to the ventral margin of the dentary [18]. In dorsal view, the rostral portion of the dentary, including the medial process, forms a spout-shaped tip that is present in all ornithischians except *Eocursor* [96] and heterodontosaurids [97]. The dorsal and ventral margins

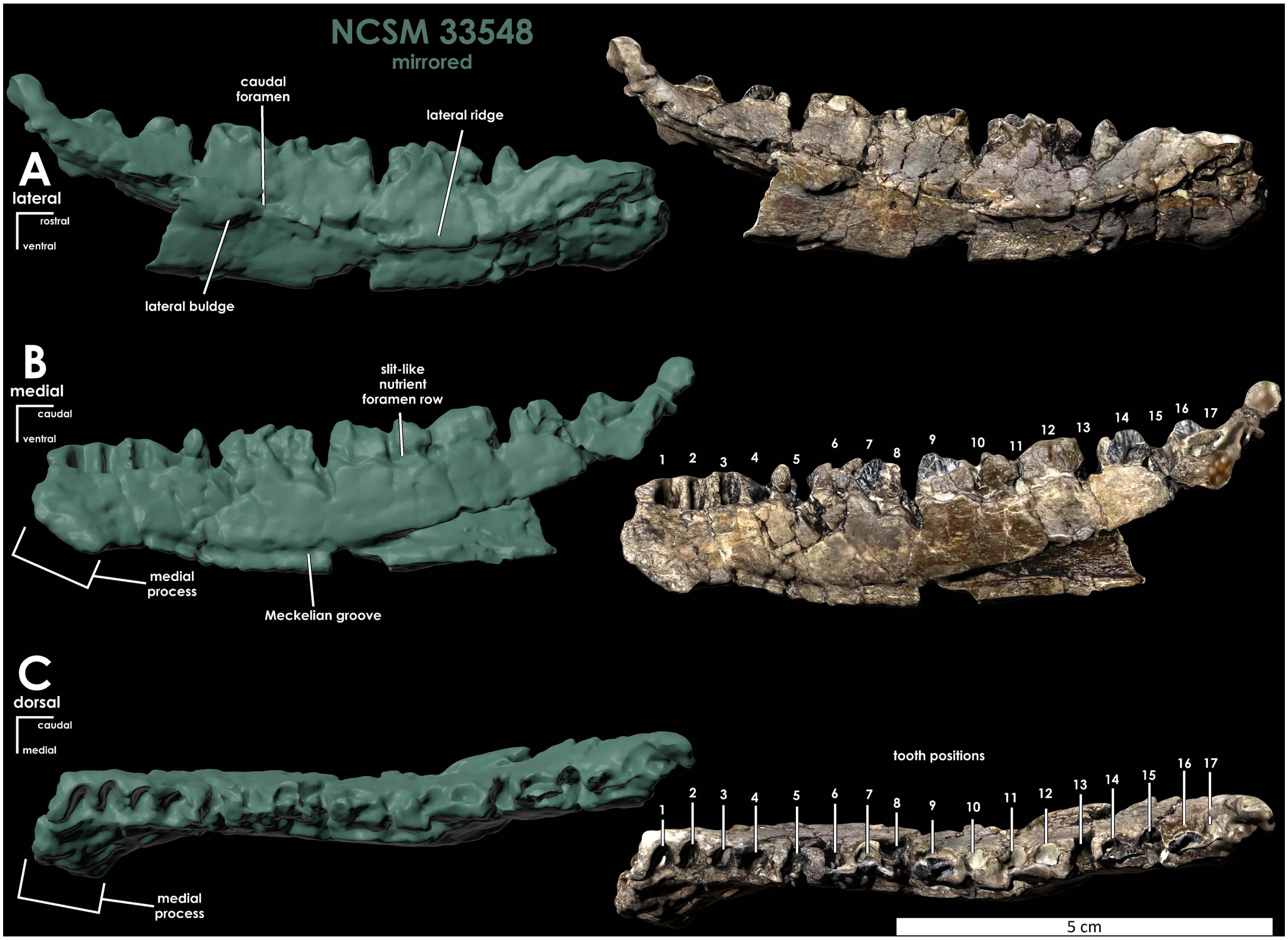

Fig 25. Dentary of *Fona herzogae.* Dashed lines represent missing bone. Images on the right show photographed elements. Numbers above teeth represent dentation number.

https://doi.org/10.1371/journal.pone.0353169.g025

of the spout-shaped tip form facets for the predentary. The spout-shaped tip is everted ventromedially, causing the sutural surface with the predentary to be angled medially.

Lateral Surface: The lateral surface is dorsoventrally convex and rostrocaudally straight along the rostral two-thirds. At the twelfth tooth position, the caudal third of the dentary becomes slightly caudolaterally deflected, except on NCSM 36130, in which this deflection is more prominent, likely due to the poor preservation and extensive cracking of the specimen along its caudal region.

The angle of the rising coronoid process relative to the tooth row is 160°. A similar shallow angle is shared by *Lesothosaurus* (MNHN LES 17) [45] and possibly *Parksosaurus.* This is in contrast to *Th. neglectus, Orodromeus, Haya, Gasparinisaura,* and *Hypsilophodon,* in which this angle is closer to 140°. There is a subtle lateral ridge that extends across the length of the dentary and is positioned halfway between the dorsal and ventral margins of the dentary. The caudal-most portion of the ridge begins at the base of the coronoid process, as in *Th. neglectus* [74]. This ridge follows an arching

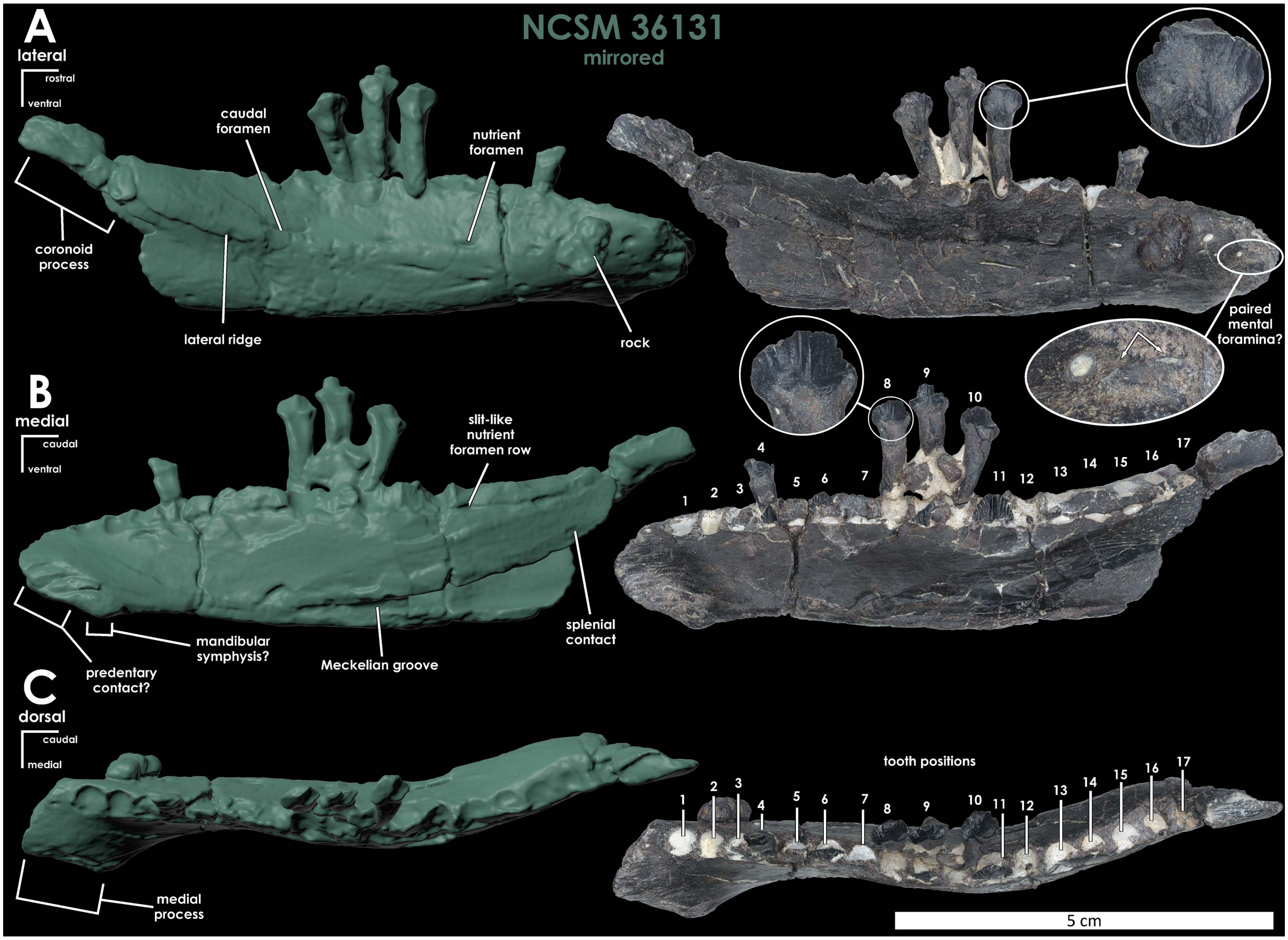

Fig 26. Dentary of *Fona herzogae*. Dashed lines represent missing bone. Images on the right show photographed elements. Numbers above teeth represent dentation number.

https://doi.org/10.1371/journal.pone.0353169.g026

path, running slightly rostroventrally until the midpoint of the dentary's length, where it curves rostrodorsally and becomes less pronounced.

Nutrient foramina: Directly dorsal to the lateral ridge is a row of nutrient foramina that vary from three to five between elements. These nutrient foramina are present in all genasaurians [77] and the heterodontosaurids *Fruitadens* [98] and *Tianyulong* [38]. Directly ventral to the tooth row on the medial surface is a row of closely spaced, rostrocaudally elongate, oval foramina (often referred to as special foramina). These foramina are also present in *Changchunsaurus* [19], *Haya* [25], *Hypsilophodon* [7], and a number of other ornithischians [99]. These foramina are connected to each other via a shallow dental groove.

A larger, caudally placed foramen is present ventral to the twelfth tooth position. This foramen is present in *Changchunsaurus* [19] and *Th. neglectus* [18], but not *Haya* [25].

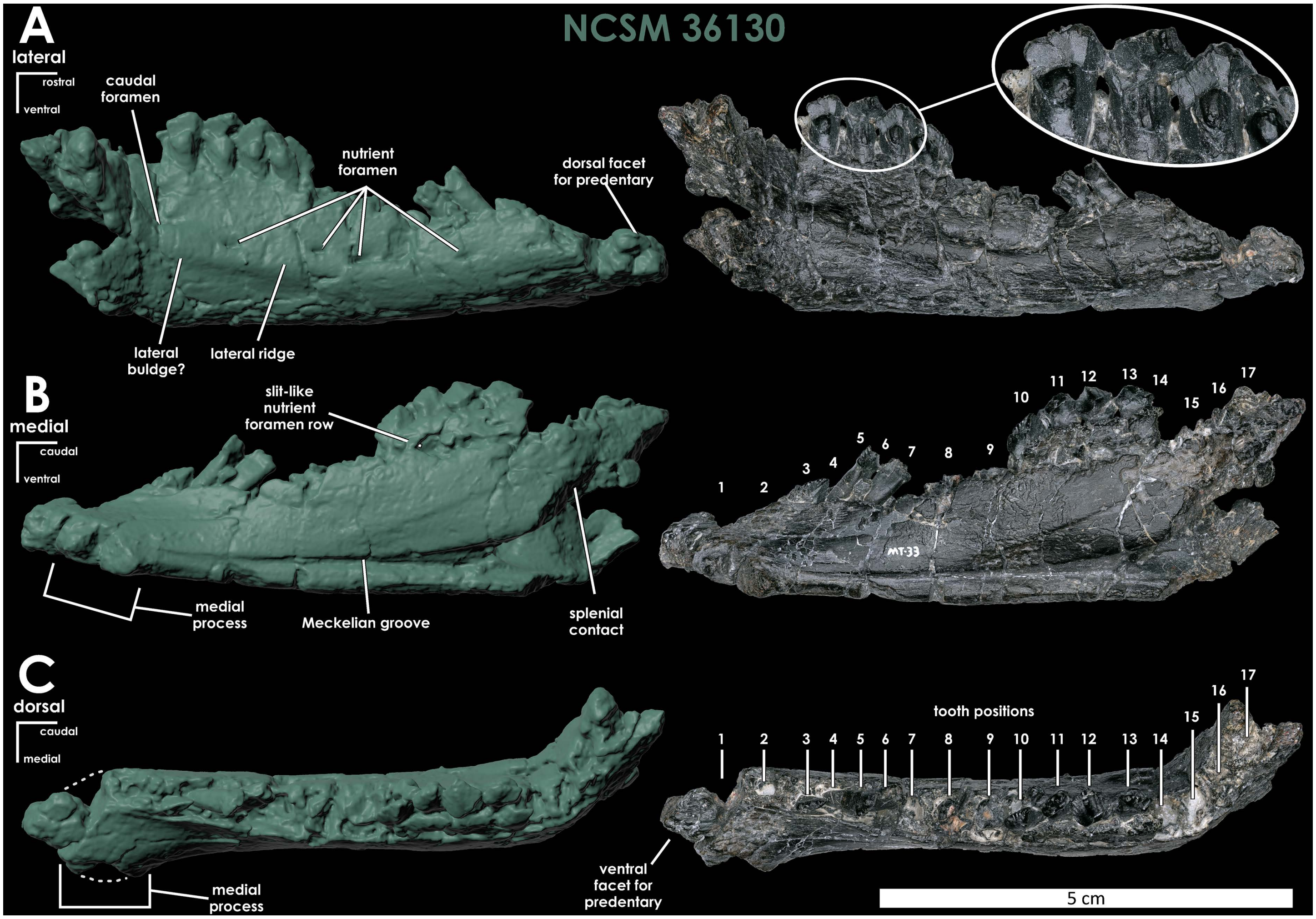

Fig 27. Dentary of *Fona herzogae*. Dashed lines represent missing bone. Images on the right show photographed elements. Numbers above teeth represent dentation number.

https://doi.org/10.1371/journal.pone.0353169.g027

The lateral surface of the dentary is rough and bears several elongate excavations and numerous small foramina, especially towards the rostral tip. The paired mental foramina of *Haya* [25], which are situated within the lateral groove that continues from the predentary, are possibly present in *Fona* (NCSM 36131) (Fig 26 A). However, if this groove and foramina complex are homologous to the structure seen in *Haya,* the lateral groove is comparatively less distinct in *Fona*.. A single foramen located in this lateral groove is also present in *Orodromeus* (MOR 1136). The rostral end of the dentary lateral surface does possess a series of randomly scattered foramina of variable size, a condition shared by a number of neornithischians, including *Th. neglectus* and *Jeholosaurus*.

Meckelian groove: The Meckelian groove extends across the length of the ventral portion of the medial face and runs parallel to the ventral margin, attenuating rostrally until immediately caudal to the mandibular symphysis. Rostrally, the Meckelian groove is shallow and dorsoventrally broader than mediolaterally deep, as in *Th. neglectus* [18]. As it continues caudally, the groove increases in dorsoventral height and significantly deepens laterally into the dentary. CT scans indicate that the margins of the groove then redirect dorsolaterally to converge ventrolaterally to the roots of the dentary teeth.

Ventral Margin: The ventral surface of the dentary is strongly convex mediolaterally and slightly rostrocaudally, creating a ventral margin that bows slightly ventrally in lateral view. Straight to slightly ventrally bowed ventral margins also occur with varying degrees of prominence in a number of SBEDOs, such as *Changmiania* [23], *Orodromeus* [29], *Jeholosaurus* [22], *Parksosaurus* [80], TMP 1997.128.0002 [61], *Oryctodromeus* [20], and *Haya* [28,100]. In contrast, some SBEDOs have ventral margins that are rostrocaudally concave such as *Th. neglectus* [18], *Changchunsaurus* [19], and *Weewarrasaurus* [55].

Dorsal Margin: The dorsal and ventral margins are not parallel but gently converge rostrally as the dorsal margin (= tooth row) slants slightly rostroventrally relative to the ventral margin. There is no evidence of rugosity near the rostrodorsal portion of the dentary, as in *Changchunsaurus* [19].

Dorsal view: In dorsal view, the tooth row is located evenly between the lateral and medial surfaces. The tooth row is not deeply recessed, and there is no prominent buccal platform as in *Changmiania* [23], *Th. neglectus* [18], *Jeholosaurus* [22], and *Changchunsaurus* [19].

In dorsal view, the tooth row is relatively straight rostrocaudally on NCSM 33548 and NCSM 36130, as in TMP 1997.128.0002 [61], *Weewarrasaurus* [55], and *Qantassaurus* [43]. In contrast, NCSM 36131 appears broadly, medially bowed, a condition shared by *Yandusaurus* [66], *Th. neglectus*, *Changchunsaurus* [19], unnamed ornithopod material from the Late Jurassic Lourinhã Formation of Portugal [101].

Dental wear patterns: The dentaries from the Mini Troll (NCSM 36130 and NCSM 36131) possibly represent a contralateral pair. The dentaries preserve fully erupted distally located teeth with varying degrees of tooth wear across their labial surfaces. The teeth of NCSM 36130 show a moderate stage of wear, with distinct vertical labiodistal and labiomesial wear surfaces, the former terminating basally at a distinct unworn shelf, whereas the latter continues ventrally toward the root. In contrast, the distal teeth of NCSM 36131 show a substantially progressed stage of wear, such that the distinction between the labiodistal and labiomesial surfaces is obscured, and the prominence of the basal shelf is substantially reduced. Detailed descriptions of the premaxillary, maxillary, and dentary dentitions are presented at the end of the osteology section, following the descriptions of all cranial elements.

Coronoid

Preservation: A single right coronoid is preserved from NCSM 33548.

General shape: It is a mediolaterally compressed, “L” shaped bone, independent from the rising coronoid process of the dentary (Fig 28).

Articulations: The coronoid likely connects to the dentary laterally, the surangular caudodorsally, and the splenial ventromedially.

Mediolateral view: In medial view, the coronoid forms an “L” shaped profile, with a larger dorsally directed process and a smaller rostrally directed process.

Dorsal process: The dorsal process of the coronoid is rostrocaudally expanded, terminating in a sharp point at its apex, as in *Hypsilophodon* [10], in contrast to the more lobate process of *Th. neglectus* that lacks a distinct dorsal point [18], or the more rectangular processes of *Lesothosaurus* [3], *Heterodontosaurus* [102], and *Manidens* [2]. The lateral surface of the dorsal process is marked by a moderately pronounced ridge oriented dorsoventrally and offset slightly from the rostral margin, which would have facilitated the contact with the rising coronoid process of the dentary. A similar surface is present in *Th. neglectus,* which appears to be formed by a more concave facet [18: fig. 16 I]. The caudal corner of the dorsal process bears a deep facet, which likely received the dorsal corner of the surangular, as it does in *Hypsilophodon* [10].

Rostral process: The rostral process is short, but this is likely due to incomplete preservation. The rostral margin between the two processes is gently curved and forms an angle of ~130°. This angle is similar to *Th. neglectus* [18], whereas in *Hypsilophodon,* this angle is wider (~145°) [10] and in *Lesothosaurus,* the rostral process is nearly parallel with the dorsal process at 170° [3]. The medial surface of the coronoid’s rostral process is slightly concave, possibly to accept

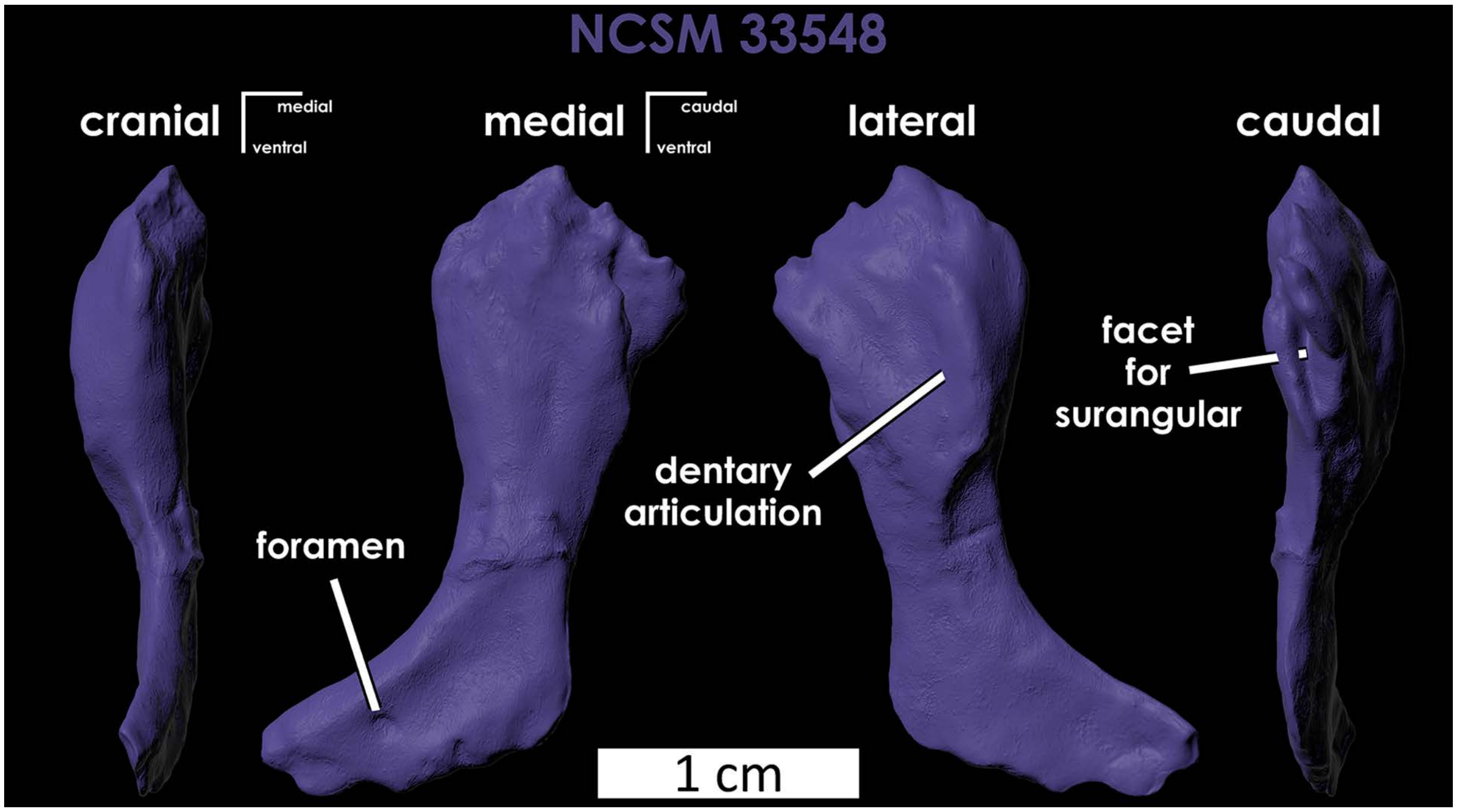

Fig 28. Coronoid of *Fona herzogae*.

https://doi.org/10.1371/journal.pone.0353169.g028

the flat dorsal process of the splenial, which contacts this region in *Th. neglectus* [18]. A single foramen occupies this concavity. The caudoventral corner of the coronoid lacks the short ventral process present in *Th. neglectus* [18], *Hypsilophodon* [10], and *Lesothosaurus* [3].

Surangular

Preservation: In total, four surangulars of *Fona* are preserved, representing at least three different individuals. The Mini Troll locality preserves a complete right (NCSM 36166) (Fig 29), a poorly preserved nearly complete right (NCSM 36167) (Fig 30), and an incomplete left (NCSM 36168) (Fig 31). The Karmic locality preserves the caudal half of a well-preserved left (NCSM 33548) (Fig 32). Although all four elements are comparable in size, varying at most by only ~5% when measured across the dorsoventral height of the retroarticular process, they display a number of morphological differences.

General shape overview: The surangular is a tripartite, sigmodal-shaped bone reminiscent of an Arabic sad initial form "صـ" in lateral view. It is characterized rostrally by an obtuse triangle-shaped, mediolaterally flattened, sheet-like bony plate, a more robust central region bearing two distinct processes, and caudally by a mediolaterally compressed process that bows slightly ventrally.

Articulations: The surangular forms the caudoventral portion of the mandible and connects to the coronoid rostrodorsally, the dentary rostrally, the splenial rostromedially, the angular ventrolaterally, the articular ventromedially, the prearticular caudally, and articulates with the quadrate caudodorsally.

Rostral process: The triangular-shaped process of bone that forms the rostral two-thirds of the surangular is mediolaterally compressed and oriented flat across the sagittal plane. In medial view, the medial surface is concave, following the early diverging ornithischian condition [18], whereas the lateral surface is mostly flat to slightly convex, as in *Th. neglectus* and *Changchunsaurus* [18,19].

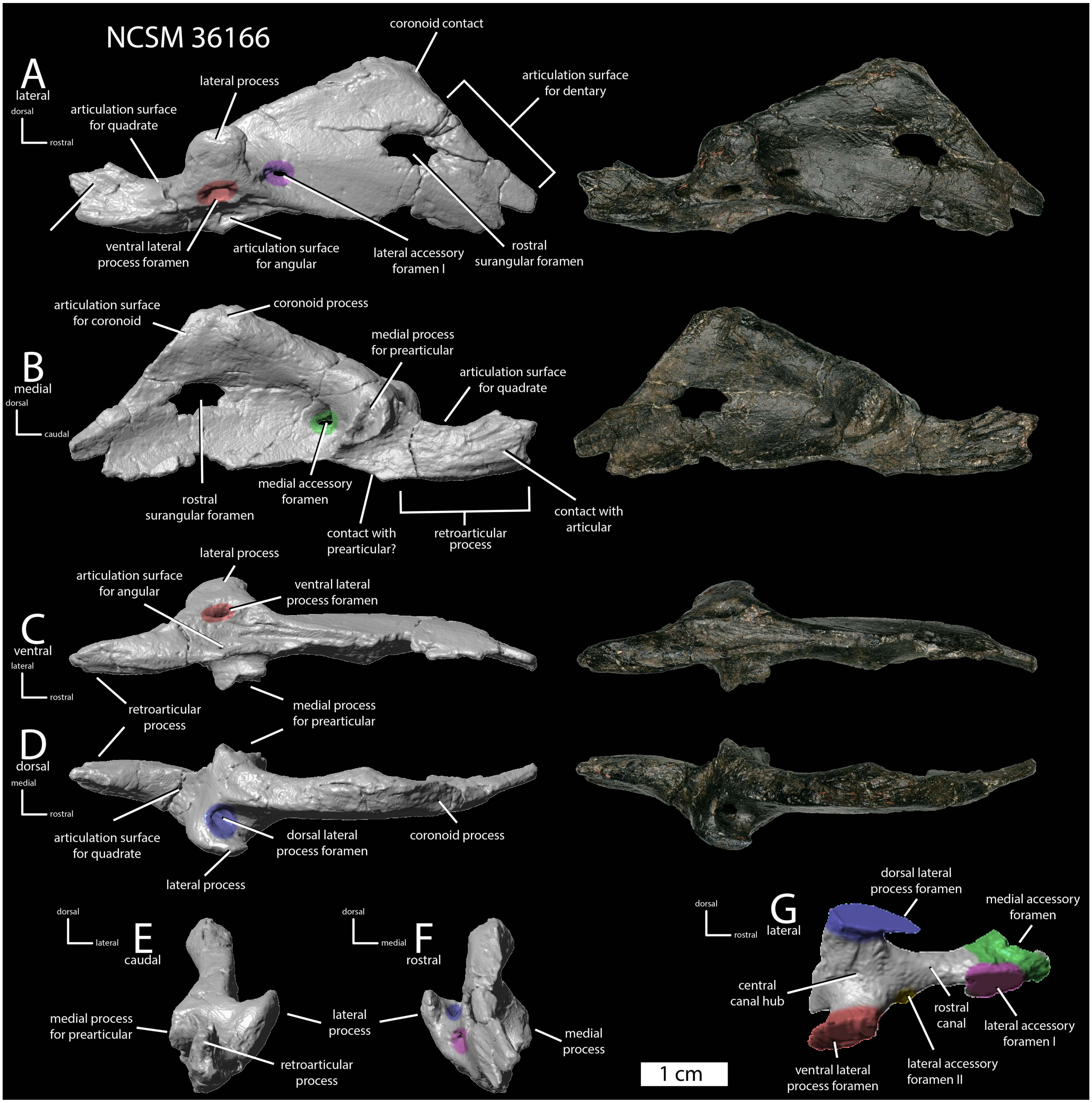

Fig 29. Surangular of *Fona herzogae.* Images on the left show 3D model. Images on the right show image stitched photograph. **G,** lateral view of the internal vascular canal labyrinth.

https://doi.org/10.1371/journal.pone.0353169.g029

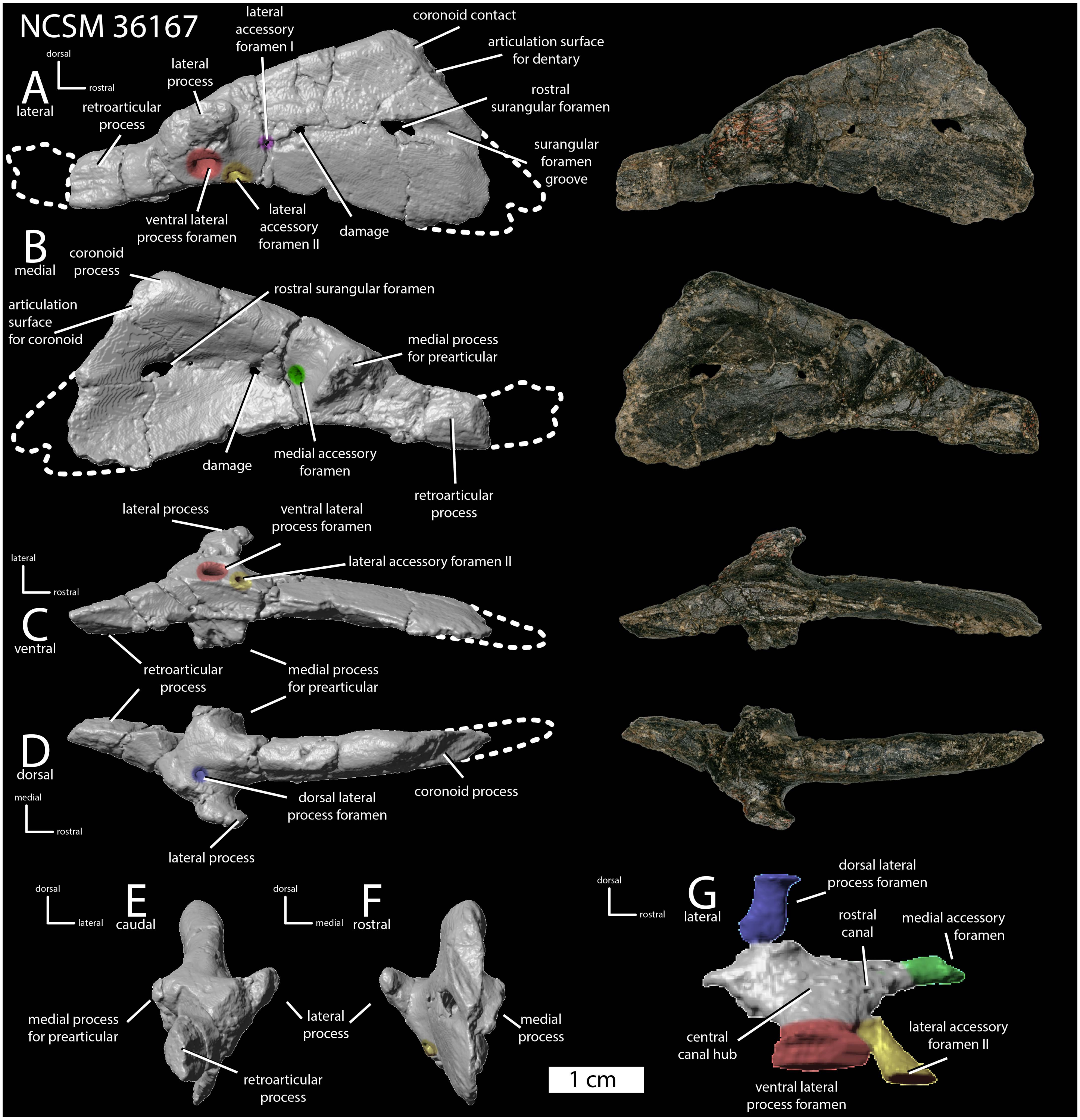

Fig 30. Surangular of *Fona herzogae*. Images on the left show 3D model. Images on the right show image stitched photograph. **G,** lateral view of the internal vascular canal labyrinth. Dashed lines represent missing bone.

https://doi.org/10.1371/journal.pone.0353169.g030

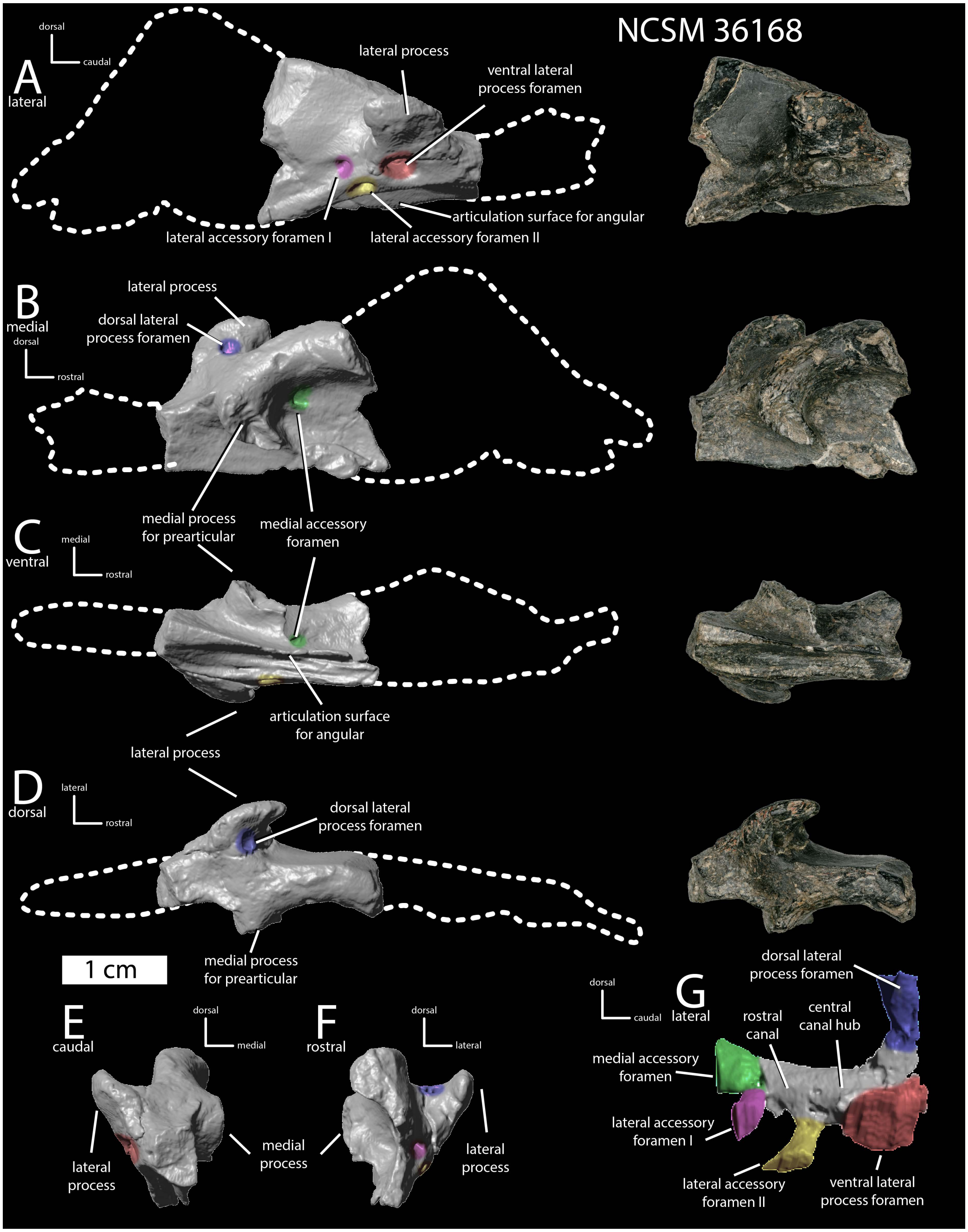

Fig 31. Surangular of *Fona herzogae.* Images on the left show 3D model. Images on the right show image stitched photographs. **G,** lateral view of the internal vascular canal labyrinth. Dashed lines represent missing bone.

https://doi.org/10.1371/journal.pone.0353169.g031

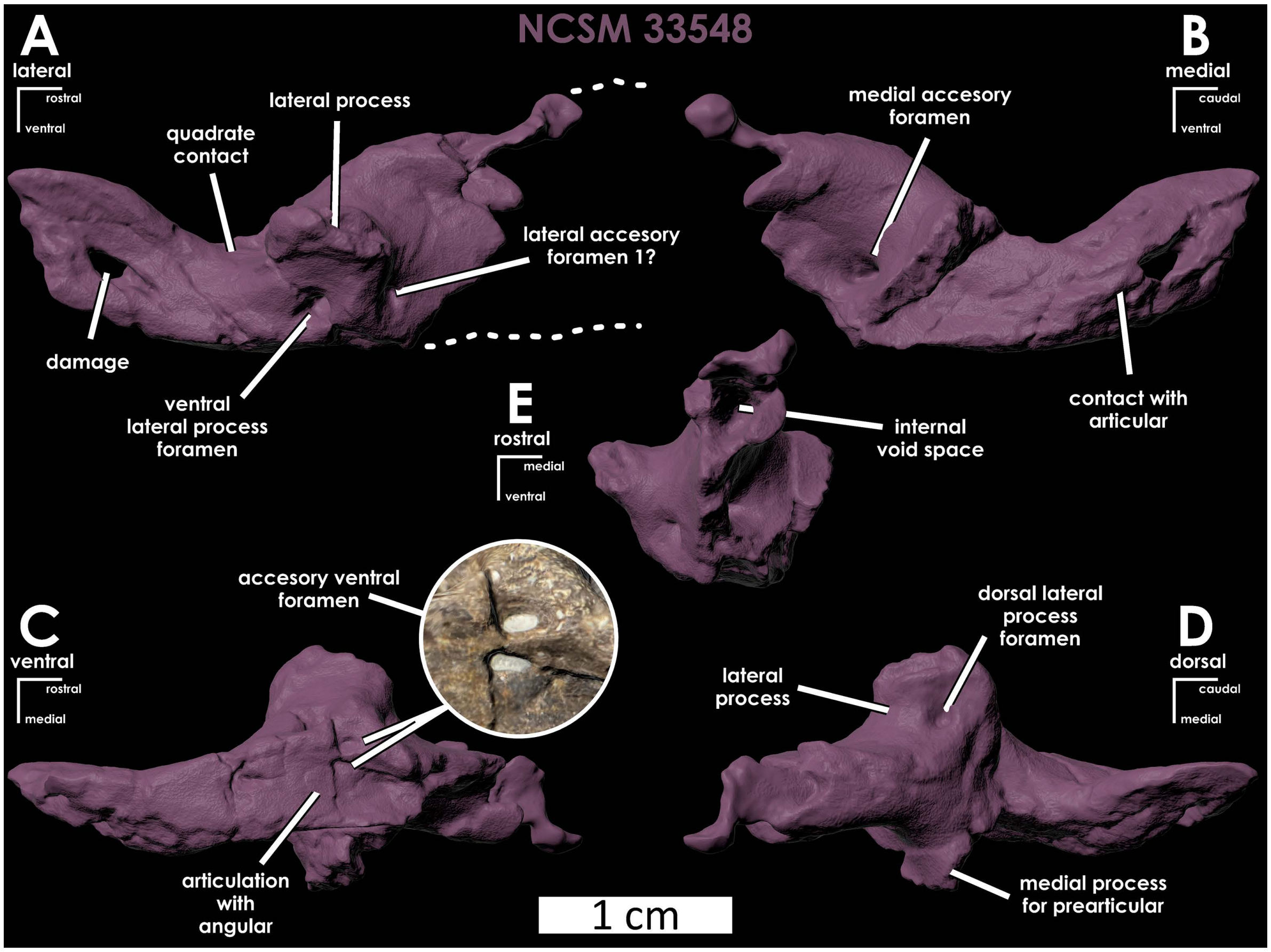

Fig 32. Surangular of *Fona herzogae*. Dashed lines represent missing bone.

https://doi.org/10.1371/journal.pone.0353169.g032

This rostral triangular process is thickest along its caudodorsal margin, which is defined by a medially inset cylindrically thickened region that forms a rostrodorsally oriented angle of approximately twenty-six degrees from the long axis of the surangular. This thickened region terminates at the dorsal-most point of the surangular, where it likely contacts the coronoid.

The rostral-most region of the triangular sheet is slightly medially deflected in dorsal view. The rostrodorsal edge is overlapped by the caudomedial portion of the dentary.

lateral process of surangular: Directly caudal to the triangular sheet is the most robust region of the surangular. This region is characterized by two prominent processes located on the medial and lateral surfaces.

The lateral process is the larger of the two. It appears as a finger-like process that emerges laterally from the surangular, deflecting dorsally and somewhat rostrodorsally. A similar process is found on *Th. neglectus* [18], *Talenkauen* [37], the heterodontosaurid *Manidens* [2], and the iguanodontians *Te. tilletti* [4] and *Zalmoxes robustus* [40]. However, the process is most similar to the structure seen in *Th. neglectus*, being dorsoventrally taller rather than rostrocaudally wide. This process is not present in *Orodromeus*, *Zephyrosaurus*, *Changchunsaurus, Haya*, or *Hypsilophodon* [7,19,21,28,29,103]. Rather, these taxa possess a low boss located in the same region [18].

Medial process of surangular: The medial process of the surangular is located on the opposite side of the surangular from the lateral process and forms the contact with the prearticular. It projects medially and is marked by a prominent

concave depression that encompasses the entirety of the medial head of the process. This process is also present in *Lesothosaurus* [3], *Hypsilophodon* [7], *Th. neglectus* [18] and possibly *Talenkauen* [37].

Articulation surface for the angular: The articulation surface for the angular is located along the ventral margin of the surangular, directly ventral to the lateral and medial process, and appears as either a thin, medially offset sheet of bone on NCSM 36166 and 36168, or simply a shallow fossa on NCSM 36167 and 33548.

On the most complete surangular (NCSM 36166), the ventral margin of the surangular follows a nearly straight path starting at the retroarticular process, across the articulation surface for the angular, and ending at the rostral tip of the surangular. In contrast, the ventral margin of the less well-preserved right surangular (NCSM 36167) appears to bow dorsally by ~160°.

Retroarticular process: The caudal fourth of the surangular is characterized by the retroarticular process and is well preserved on NCSM 36166 and 33548, whereas much of the process is damaged on the other two. This process is mediolaterally compressed and oriented rostrocaudally. It is mediolaterally thicker rostrally, attenuating in transverse thickness caudally.

In lateral and medial views, the ventral margin of the process bows slightly ventrally. The dorsal margin of the process begins directly caudal to the dorsal aspect of the medial process and bows ventrally by 130 degrees, forming an articulation surface for the ventral condyles of the quadrate. The caudal half of the retroarticular process is characterized by a series of rostrocaudally oriented grooves on the lateral and medial surfaces, similar to those in *Th. neglectus* [18]. These grooves are more pronounced on the medial surface, which is slightly convex, whereas the lateral surface is slightly concave. They likely served as the attachment site for the *m. pterygoideus ventralis* [104].

Foramina: There are three distinct groups of foramina located on the surangular: the lateral process foramina and the accessory foramina, which contribute to the internal vascular canal labyrinth, and the surangular foramina.

Surangular foramen: Numerous terminological inconsistencies persist regarding the inferred homology of surangular foramina across ornithischian clades [sensu 1]. In lateral and medial view, there is a large mediolaterally oriented foramen, often described as the surangular foramen, which may in fact represent the remnant of the external mandibular fenestra [78], located slightly rostral to the center of the large rostral triangular plate-like process and just caudal to the contact with the dentary. It is about twice as large on NCSM 36166 versus NCSM 36167. It pierces the surangular along a rostrolateral orientation, as evidenced by the fossa located directly rostral to the opening. This rostral surangular foramen is present in *Orodromeus*, *Oryctodromeus*, *Th. neglectus, Haya, Changchunsaurus, Gasparinisaura, Hypsilophodon,* and *Jeholosaurus*, the early diverging ceratopsian *Yinlong,* the heterodontosaur *Manidens,* and many early diverging iguanodontians [2,7,18,19,22,25,29,39,56,105].

Dorsal lateral process foramen: There are two foramina located on the dorsal and ventral margins of the lateral process. The dorsal foramen of the lateral process originates at the central canal hub of the internal vascular canal labyrinth and projects directly dorsally. The opening is located at the embayment between the lateral surface of the surangular and the base of the lateral process; within this embayment, the opening is offset caudally. The diameter of the opening on NCSM 36166 is large and similar in size to the ventral lateral process foramen. In contrast, the diameter of the opening on NCSM 36167 and 36168 is significantly reduced, relative to their respective ventral foramina, while on NCSM 33548, they are equivalent in size.

Ventral lateral process foramen: The ventral foramen of the lateral process originates at the central canal hub of the internal vascular canal labyrinth and projects ventrolaterally. It is roughly equally sized on all four surangulars and opens at the ventrolateral portion of the process. On NCSM 36166, the opening is positioned centrally along the vertical long axis of the process, whereas on NCSM 36167, and especially NCSM 36168, the opening is offset rostrally.

The placement of the ventral opening on the left surangular of *Th. neglectus* (NCSM 15728) is located slightly rostral to the ventral margin of the lateral process [18], whereas its right surangular has a second ventral opening located in a similar position to the ventral opening on *Fona*. The lateral process foramen of *Th. neglectus* (NCSM 15728) does not exit

dorsally, as in *Fona*, but rather is oriented rostrally and exits along the medial surface of the surangular, slightly rostral to the medial process [18]. Additionally, Boyd [18] notes that other neornithischian taxa possess similar foramina located in this area, despite lacking a lateral process (e.g., *Changchunsaurus, Haya, Hypsilophodon*).

Accessory foramen: There are three accessory foramina that differ in size and location across all surangulars. These structures may be variably homologous to foramina present in similar regions of the surangular of other neornithischians such as *Changchunsaurus* and *Hypsilophodon* [7,19].

Medial and lateral accessory foramen I: The medial and lateral accessory foramen I (laf I) are located on the caudal portion of the rostral triangular process and are present on each surangular. Together, they represent the rostral-most branch of the internal vascular canal labyrinth and diverge in close proximity to one another. In NCSM 36166, the bifurcation occurs so near the external surface that the two openings effectively form a single mediolaterally piercing window. In contrast, the bifurcation occurs slightly more caudally in NCSM 36167 and 36168, resulting in more distinct external openings. In NCSM 36167, the opening of laf I coincides with a crack in the surangular and is therefore difficult to identify externally without microscopic examination. The damage in this region also obscures the internal course of the canal in the CT data. In NCSM 33548, the identity of laf I cannot be confirmed with certainty. The foramen occurs in the region where laf II would be expected based on the morphology of the three surangulars from the Mini Troll locality; however, it is positioned mediolaterally opposite the medial accessory foramen, a configuration more consistent with laf I. Unfortunately, most of the rostral triangular process is missing in NCSM 33548, preventing confident assessment of the canal morphology and homologous identity of this opening.

Lateral accessory foramen II: The lateral accessory foramen (laf II) is present in all three surangulars from the Mini Troll locality. This foramen originates caudal to laf I, but rostral to the ventral lateral process foramen branches directly from the central canal hub of the internal vascular labyrinth. It projects rostrolaterally and ventrally, forming the ventral-most external opening on the surangular. The opening is positioned along the ventrolateral margin of the surangular, slightly rostral to the articulation surface for the angular. In NCSM 36166, the opening of laf II is partially obscured by damage; however, a shallow external depression and remnants of the canal indicate that the foramen was originally present. In NCSM 33548, either laf I or laf II is present, along with two additional pinhole-sized foramina located slightly rostroventral to the ventral lateral process foramen within the fossa for the angular articulation (Fig 32C).

Accessory foramina associated with, or adjacent to, a lateral process or lateral boss, and distinct from the accessory foramina that pierce the rostral triangular sheet, are also present in *Hypsilophodon* [7: fig 10] and *Manidens* [2: fig 13].

Angular

Preservation: A single well-preserved angular was recovered from the Karmic locality (NCSM 33548), whereas three angular were recovered from the Mini Troll locality: a well-preserved right (NCSM 33548), a well-preserved right missing portions of its rostral and caudal tips (NCSM 36125), and a partially preserved left missing its caudal half (NCSM 36127).

General shape: The angular is a rostrocaudally elongate, trough-shaped bone, characterized by a larger lateral wing, a smaller medial wing, and a deeply concave "scoop-shaped" rostral half (Fig 33).

Articulations: It forms the caudoventral and lateral portion of the mandible and connects with the surangular dorsolaterally, the articular caudodorsally, likely the prearticular caudomedially, and the dentary rostrally.

Lateral surface: The lateral wing of the angular is dorsoventrally taller than the medial wing, as in *Th. neglectus* [18]. In lateral view, the dorsal margin of the lateral wing forms a gently raised convex margin. The lateral surface is flat and lacks the prominent depression that is present in *Th. neglectus* [18] and *Te. tilletti* [4]. In lateral view, the rostral half of the angular would have contacted the caudomedial surface of the dentary by inserting into the Meckelian canal.

Dorsal surface: In dorsal view, the angular is mediolaterally concave, forming a distinct rostrocaudally oriented trough. The caudal half of this trough is shallow, whereas the rostral half becomes twice as deep. This differential is demarcated by a sudden ventral step at the mid-length of the angular on NCSM 36126 and 36127. This change in depth is slightly less

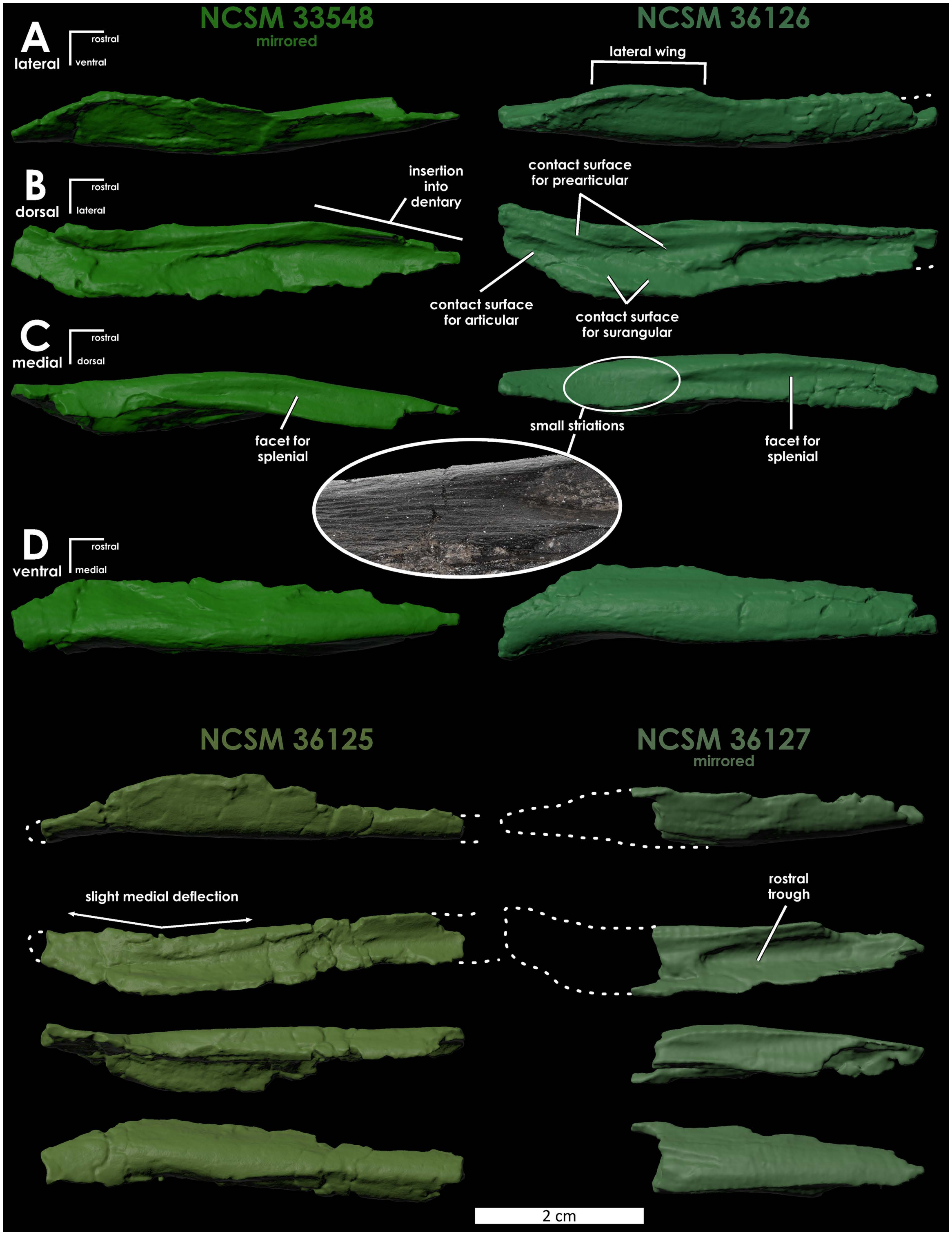

Fig 33. Angular of *Fona herzogae*. Dashed lines represent missing bone.

https://doi.org/10.1371/journal.pone.0353169.g033

pronounced on NCSM 36125, possibly due to poor preservation of the bone surface. On NCSM 33548, the ventral deepening of the trough follows a gentle slope with faint demarcation located more caudally than the angular from the Mini Troll locality. Caudal to the lateral trough, the shallow convexity forms a quadruple junction cradle that would have embraced the surangular laterally, the articular dorsally, and the prearticular medially, as in *Th. neglectus* [18]. A notable medial deflection also characterizes the caudal-most portion of the angular, a condition shared by *Th. neglectus* [18].

Ventral surface: The majority of the ventral margin of the angular is strongly mediolaterally rounded and slightly rostrocaudally convex as in *Hypsilophodon* [7], *Jeholosaurus* [22], *Changchunsaurus* [19], and *Orodromeus* [26], *Th. neglectus* [18]. This is in contrast to the condition in *Haya* [25] and *Changmiania* [23], in which the ventral surface is rostrocaudally concave. The caudal fifth of the angular becomes slightly concave, as in *Haya* [25]. In dorsal or ventral view, the angular is mediolaterally narrowest rostrally and becomes gradually wider caudally, where it forms the floor of the retroarticular process.

Medial surface: There is a shallow, rostrocaudally elongate groove that runs across the rostral two-thirds of the medial surface of the angular. This groove likely facilitated a connection with the splenial, as in *Th. neglectus* [18]. This groove is less pronounced on NCSM 36125. Caudal to this groove, the bone texture of the medial surface shows a pattern of thin, diminutive, densely packed, horizontal striations, a condition shared with *Lesothosaurus* [3].

Splenial

Preservation: A well-preserved, partially deformed, left splenial (NCSM 36162) and another incomplete left splenial (NCSM 36175) were recovered from the Mini Troll locality.

General shape: The splenial is a mediolaterally flattened, triradiate, sheet-like bone. It is characterized by a main body with rostral, dorsal, and caudal projections, forming an upside-down "T" shaped outline in mediolateral view (Fig 34).

Articulations: The splenial forms the caudoventral and medial portion of the mandible and connects with the surangular dorsally and the dentary rostrally.

Lateral surface: The medial surface is dorsoventrally and slightly rostrocaudally convex, whereas the lateral surface is dorsoventrally and slightly rostrocaudally concave. The medial surface is characterized by a shallow groove that runs adjacent to the ventral margin, gradually attenuating caudally. This groove would have contacted the angular.

The ventral margin is the thickest portion of the splenial and follows a straight path rostrally, converging with the rostroventrally angled margin of the dorsal process. This tapered point is deformed on NCSM 36162; however, it was likely situated near the Meckelian groove, as in *Th. neglectus* [18]. The caudal margin of the dorsal process is more dorsoventrally horizontal than the rostral margin, forming the rostral half of the internal mandibular fenestra, as in *Lesothosaurus* [3].

Foramen: No distinct rostrally positioned foramen is observed, in contrast to the condition in *Changchunsaurus* [19], *Haya* [25], and saurischian dinosaurs [106]. However, the rostral ends of both elements are damaged or incomplete, precluding definitive assessment of this feature.

Caudal process: The caudal process forms the caudal third of the splenial and would have overlapped the medial surface of the prearticular, as in *Th. neglectus* [18]. The dorsal and ventral margins of this process run in parallel and lack the bifurcation present in *Changchunsaurus*, *Archaeoceratops* [19], and possibly *Lesothosaurus* [3].

Articular

Preservation: A single well-preserved right articular is represented by NCSM 33548.

General shape: The articular is shaped like a slightly flattened tetrahedron with its pyramidion pointing laterally. It is characterized by a smooth medial surface and a complex lateral surface with multiple ridges, grooves, and facets (Fig 35).

Articulations: It connects to the prearticular medially, the angular ventrally, the surangular laterally, and the quadrate rostrodorsally.

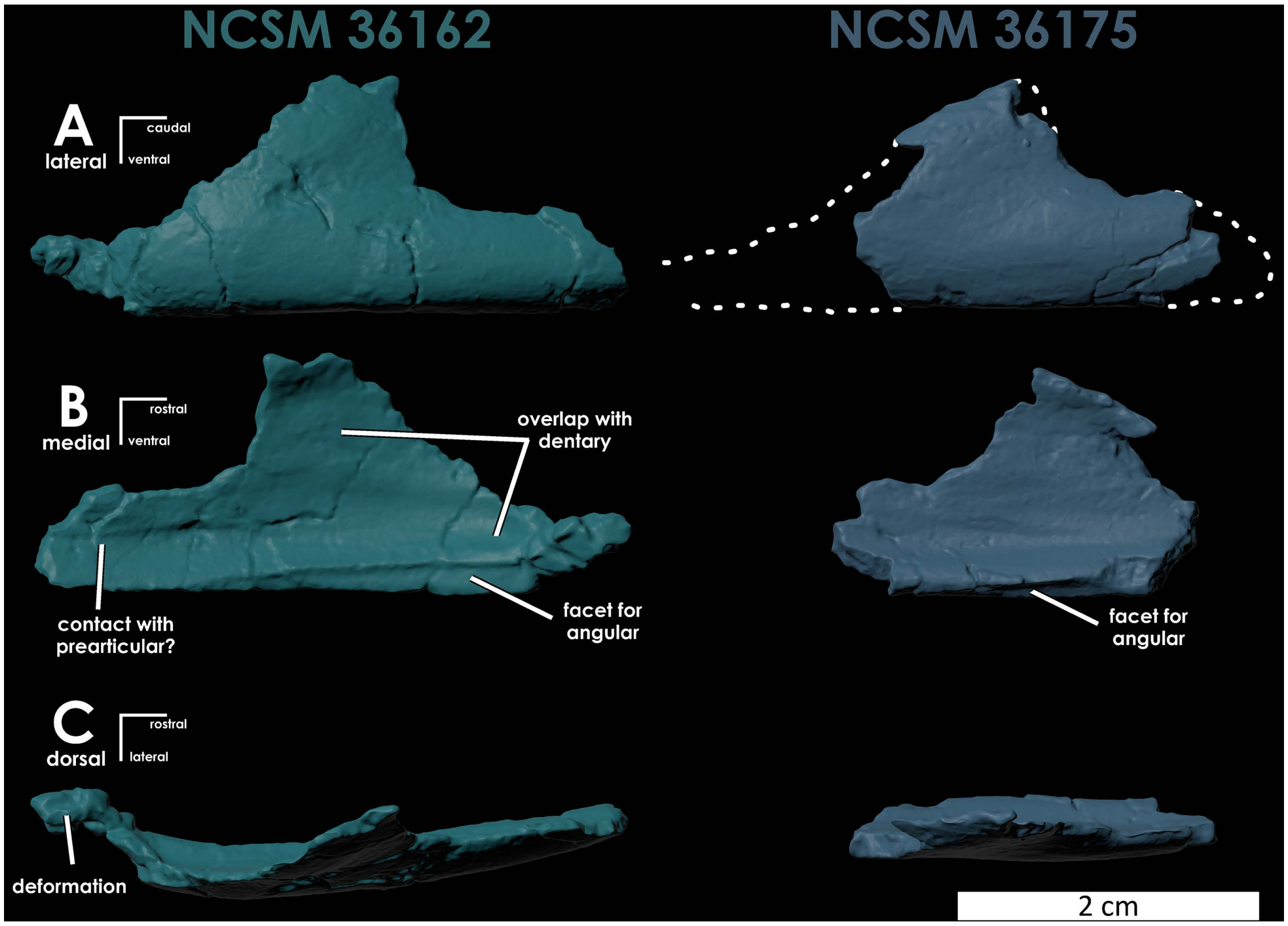

Fig 34. Splenial of *Fona herzogae*. Dashed lines represent missing bone.

https://doi.org/10.1371/journal.pone.0353169.g034

Lateral surface: In lateral view, the articular gives a triangular profile trisected by three ridges that radiate from the centroid and continue towards the three corners, with the regions between these forming slightly depressed surfaces. Comparisons with internal scans of *Th. neglectus* (NCSM 15728) suggests the current rotation of the element along the sagittal plane represents the most likely orientation. Therefore, the ventral facet would have accepted the medial surface of the retroarticular process of the surangular, whereas the rostrodorsal surface would have contributed to the glenoid for the ventral condyles of the quadrate. The facet for the surangular is dominated by several foramina of varying sizes and connects to a prominent circular depression immediately ventral to the centroid. The rostrodorsal surface for the quadrate is slightly less irregular than the surface for the surangular, yet still bears an undulating pattern. Lastly, the caudal margin of the lateral surface is dominated by a prominent and comparatively narrower concavity, the purpose of which remains unknown, which could represent the true facet for the surangular if the correct orientation of the element is represented by a roughly −110° rotation about the sagittal plane. However, a similar concavity is noted on *Haya* [25].

Medial surface: The medial surface is broadly flat and smooth and forms a slight concavity at its caudoventral corner, likely representing the contact for the lateral surface of the prearticular.

Foramen: A single foramen is present on the medial surface, slightly offset rostrally from the centroid, which likely connects with a similar-sized foramen positioned directly opposite on the lateral surface. This may represent the path of the chorda tympani branch of c.n. VII, noted to be present on a number of archosaurs, possibly including *Psittacosaurus,* yet absent on *Haya* [25].

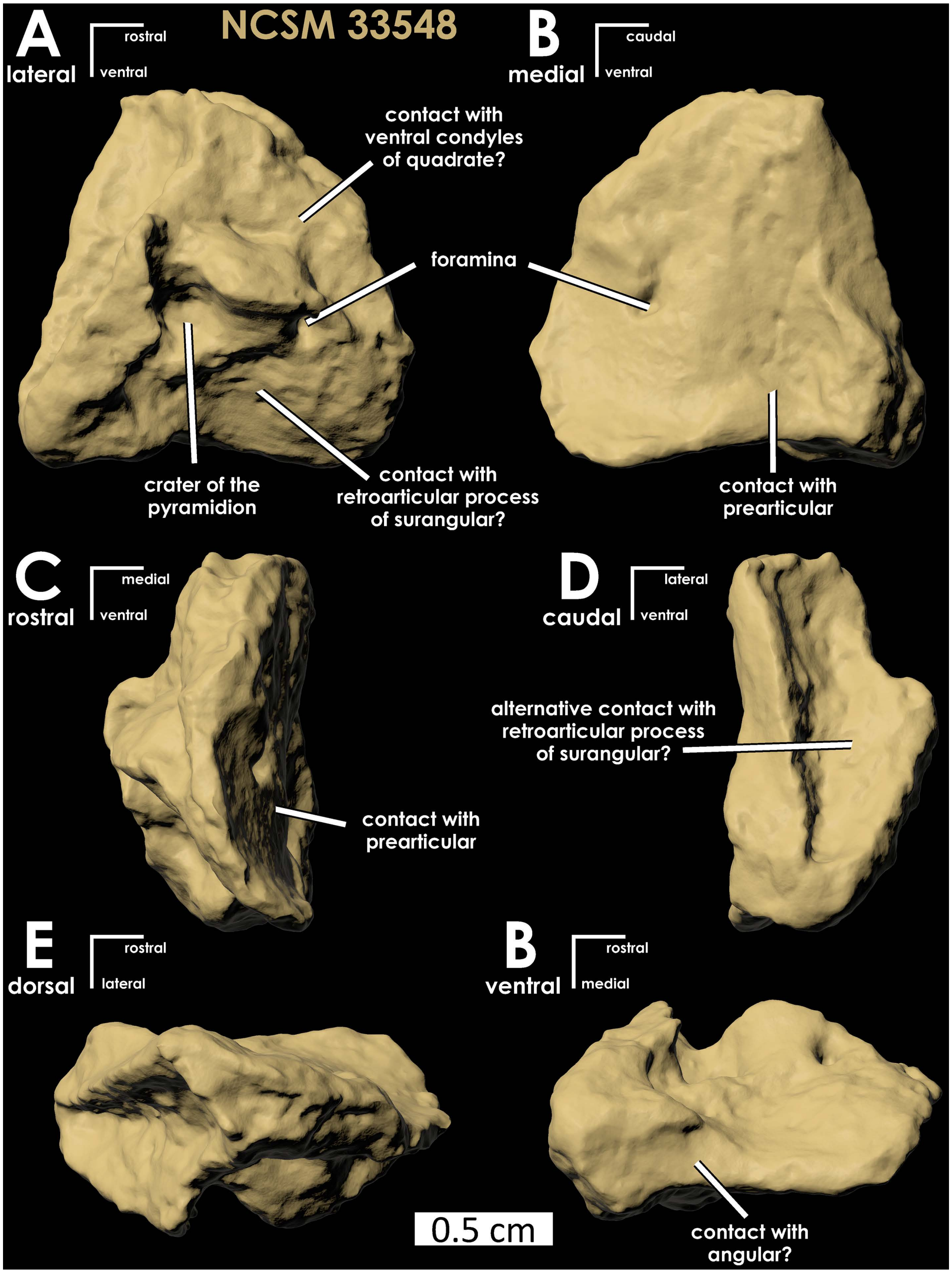

Fig 35. Articular of *Fona herzogae*.

https://doi.org/10.1371/journal.pone.0353169.g035

Premaxillary dentition

Preservation: FMNH PR 4581 is the only premaxilla that is fully preserved with erupted teeth (2, 4, 5). Approximately 20 isolated premaxillary teeth were collectively recovered from number of localities across the Mussentuchit Member including the Karmic (NCSM 33548), Mini Troll, LCT, POD, and Magic Man localities. Isolated teeth from the Karmic and Mini Troll localities are referable to *Fona herzogae,* whereas those from the other localities are currently only referable to the level of Thescelosaurinae.

General: The premaxillary teeth are labiolingually compressed and have constricted bases. They are oval in cross-section, with a slightly more convex lingual face. Teeth are slightly curved lingually, and the apices of the crowns are recurved distally (Fig 36 and S1.24–S1.31 Files).

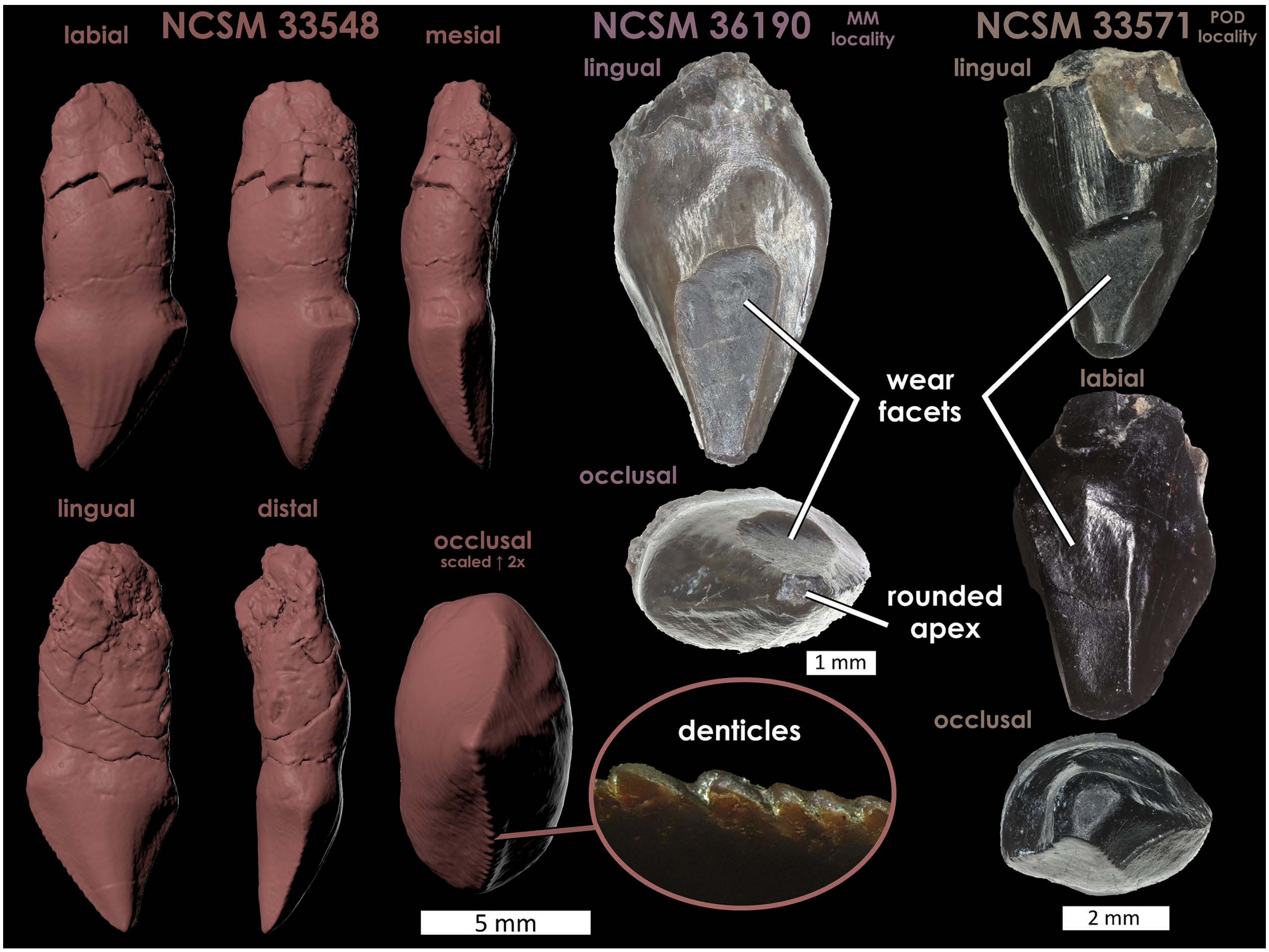

Fig 36. Premaxillary dentation of *Fona herzogae.* NCSM 36190 is an isolated tooth from the Magic Man (MM) locality which is an active fossil quarry currently preserving a single thescelosaurine [9]. NCSM 33571 is an isolated tooth from the Pit of Despair (POD) locality in the Mussentuchit, which preserves a number of multitaxic microfossils along with larger non-thescelosaurine body fossils.

https://doi.org/10.1371/journal.pone.0353169.g036

Carina and serrations: On some premaxillary teeth, the mesial and distal carinae bear weak serrations, a condition shared by *Hypsilophodon* [7], *Lesothosaurus* [24], and *Agilisaurus* [48], but not *Th. neglectus* (NCSM 15728), *Haya* [25,100], *Jeholosaurus* [22]. *Orodromeus* also lacks denticles [contra 29]. However, the teeth of MOR 1141 and 1136 show extensive wear, and it is likely that any denticles would have been worn away. Nonetheless, the carinae do occasionally show a distinct wavy pattern, possibly representing a remnant of denticles. The serrations of *Fona* are not as prominent as those in the pachycephalosaur *Stegoceras* [107] or thyreophorans such as *Scutellosaurus* [33] and *Huayangosaurus* [31].

Striations: The lingual surface is smooth, whereas the labial surface bears two to three longitudinal ridges. Subtle striations are also present in *Haya* [25]. In contrast, *Th. neglectus* bears a dense array of prominent striations that maintain a uniform thickness across both lingual and labial surfaces [18,107]. Striations are absent in *Changchunsaurus* [19], *Jeholosaurus* [22], *Hypsilophodon* [7], *Zephyrosaurus* [21,51], and *Orodromeus* (MOR 1141 and 1136).

Wear facets: Wear facets display a multitude of patterns. When teeth are minimally worn, the apex bears a small, angled wear facet on the lingual surface, as in *Th. neglectus* [18] and *Zephyrosaurus* [21]. Increased wear results in a more bluntly rounded or flattened apex, as occurs in *Th. neglectus* [18], *Jeholosaurus* [22], and *Orodromeus.* This is in contrast to *Haya* [25] and *Lesothosaurus* [3], which lacks wear facets altogether. More progressively worn premaxillary teeth bear bluntly rounded apices and a loss of denticles. NCSM 33548 and 36190 bear a prominent, flat wear facet across the lingual surface that extends halfway down the length of the tooth.

Roots: As in *Th. neglectus* [18] the cross-section of roots can vary between circular and elliptical [18].

Maxillary dentition

General: Unworn maxillary teeth are labiolingually compressed, slightly taller than wide, and mesiodistally expanded relative to their roots, forming a diamond-shaped outline in labial view (Fig 37 and S1.32 – S1.34 Files). The lingual surface is convex, whereas the labial surface is mesiodistally concave on either side of the median ridge, as occurs in *Changchunsaurus* [19].

Carina and serrations: In labial view, a small median ridge runs from the apex to the cingulum. *Lesothosaurus* [3], *Th. neglectus* [103], *Haya* [28], *Changchunsaurus* [19], *Zephyrosaurus* [21], *Changmiania* [23], *Jeholosaurus*, and a number of other early diverging ornithischians [22] possess a small median ridge or eminence and lack the prominent primary ridge seen in *Hypsilophodon* [59], *Dryosaurus* [47], *Anabisetia* [108], *Camptosaurus* [109], some Australian ornithopods [43: fig 24], some early diverging ceratopsians such as *Auroraceratops* [64] and *Heterodontosaurus* [110].

The median ridge lacks the apical spike that occurs in *Hypsilophodon* [59]. Tooth 14 on maxilla NCSM 36143 is minimally worn and preserves the entire apex. It bears a maximum of eleven denticles, with six mesial to the median ridge and five distal to it, as occurs in *Haya* [28]. Denticles extend down the length of the carina towards the base, and each denticle is confluent with a ridge that can extend to the cingulum but usually decreases in prominence basally. These ridges are more strongly delineated in *Th. neglectus* [18,103], *Parksosaurus* (ROM 804), but absent in *Orodromeus* [29] and unnamed orodromine teeth from the Belly River Group (possibly *Albertadromeus*) [61]. These ridges are narrower and more numerous than those of *Galleonosaurus* and *Leaellynasaura,* but not as numerous as those in *Atlascopcosaurus* and *Muttaburrasaurus* [43], the neornithischian *Convolosaurus* [35], or the rhabdodontid *Matheronodon* [111]. In tooth 14, the three denticles directly distal to the median ridge appear to be slightly bifurcated or subdivided. Bifurcated denticles are also known to occur in *Nanosaurus* [112] (potentially *nomina dubium* [113]), *Hypsilophodon* [59: fig. 17.15], *Changchunsaurus* [114; fig 7B], isolated neornithischian teeth from the Barremian Uña Formation in Spain [115], and are similar to the crenulated denticles in the heterodontosaur *Manidens* [54,116].

Wear facets: Each crown possesses a single wear facet on the mesiolingual surface in contrast to some of the crowns in *Zephyrosaurus* that bear two [21]. These facets form a continuous occlusal surface, as in *Th. neglectus* [18] and are angled approximately 45 degrees from the long axis of the tooth, as in *Changchunsaurus* [19] and *Galleonosaurus* [43].

When unworn, crowns appear symmetrical in labial view, as occurs in *Lesothosaurus* [24] and heterodontosaurids [52], but in contrast, the asymmetrical crowns of a number of early diverging ornithopods, such as the neornithischian *Convolosaurus* [35], *Galleonosaurus* [43] and *Talenkauen* [37]. Worn crowns show a slightly asymmetrical outline, with shallower mesial edges and steeper distal edges, creating a lateral outline that is similar to the teeth of *Hypsilophodon* [59], but not to the same degree. All teeth are asymmetrical in mesiodistal view.

Enamel: The distribution of enamel across the maxillary crowns is unknown in the absence of histological sectioning. However, on partially erupted teeth, the enamel appears to be evenly distributed between the labial and lingual surfaces, and the lingual surface bears ridges as in *Zephyrosaurus* [21]. Enamel on the labial surface of fully erupted teeth is distinctly shiny and smooth, whereas across the entire lingual surface, it appears either absent or thin, with a rough surface texture. When enamel is preserved, it bears a series of minute scratches that likely represent feeding marks. These scratches run distobasally, deepening and sometimes curving distally along their lengths.

Cingulum: Teeth are arranged en echelon. On the labial surface, the cingulum is convex across the distal edge but forms a sharp, laterally inflected ridge mesially, creating a broad, flat, mesiolabial surface that seems to serve at least partially as an occluding surface with the distolingual surface of the mesially adjacent neighbor. In lingual view, the cingulum is broadly convex, and its entire margin is strongly slanted apicodistally. This is in contrast to the more horizontal lingual cingulum present in *Lesothosaurus* [3] and *Changchunsaurus* [19]. A cingulum appears absent in *Atlascopcosaurus* [117,118].

Roots: Roots are mostly straight, broadly spaced apart, and do not contact each other.

Dentary dentition

General: All dentaries preserve 17 tooth positions. Unworn dentary teeth are labiolingually compressed, slightly taller than wide, and mesiodistally expanded relative to their roots, forming a diamond-shaped outline in labial view (Fig 37 and S1.35–S1.37 Files). The lingual and labial surfaces are convex, creating an oval cross-section.

Carina and serrations: In lingual view, there is a well-defined primary ridge, as opposed to the faint median ridge on the labial surface of maxillary teeth. This primary ridge is not present on the labial surface, as in *Changchunsaurus* [19].

Mesialdentary tooth: Tooth three on dentary NCSM 36130 is the only mesial tooth preserved and is nearly completely exposed (Fig 27A). It appears generally smaller and more mesiodistally narrow than distal crowns, as in *Haya* [25], *Orodromeus* [29], and *Th. neglectus* [18]. It is straight and symmetrical in both labiolingual and mesiodistal views, as opposed to the distally curved mesial teeth of *Th. neglectus* [18] or the recurved teeth of *Agilisaurus* [48] and *Stegoceras* [107]. In lingual view, the tooth bears at least 13 denticles, with seven mesial to the primary ridge and at least six distal to it.

Distal dentary tooth: Unworn distal teeth are symmetrical in labiolingual view, as in *Th. neglectus* [18], *Oryctodromeus* (IMNH 44946), and *Haya* [25]. Teeth are asymmetrical in mesiodistal view, as in *Changchunsaurus* [19].

Teeth bear 12–14 denticles that span the length of the carinae. Each denticle is confluent with a ridge that extends towards the crown base, as occurs in a number of early diverging ornithischians [18]. These ridges often curve along their lengths slightly inward toward the primary ridge. They are just as prominent as the ridges on the maxillary teeth, in contrast to *Jeholosaurus* [22], in which the ridges are more distinct on the maxillary crowns. Tooth 12 on NCSM 36130 shows that ridges can be present on the lingual surface, as occurs in *Th. neglectus, Parksosaurus,* and *Zephyrosaurus* [61]. However, these ridges are faint and usually absent on most erupted and partially erupted dentary teeth.

Cingulum: Distal dentary teeth are arranged en echelon, with the distolabial surface of each crown occluding with the mesiolingual surface of its distally adjacent neighbor. However, the extent of overlap between crowns seems less extensive than the maxillary crowns. Near the base of the crown, the distal carina bears a medially inflected ridge, similar but less pronounced than those of the maxillary crowns. This ridge is also found on unnamed orodromine teeth from the Belly River Group (possibly *Albertadromeus*) and is present in a number of other early diverging ornithischians [61].

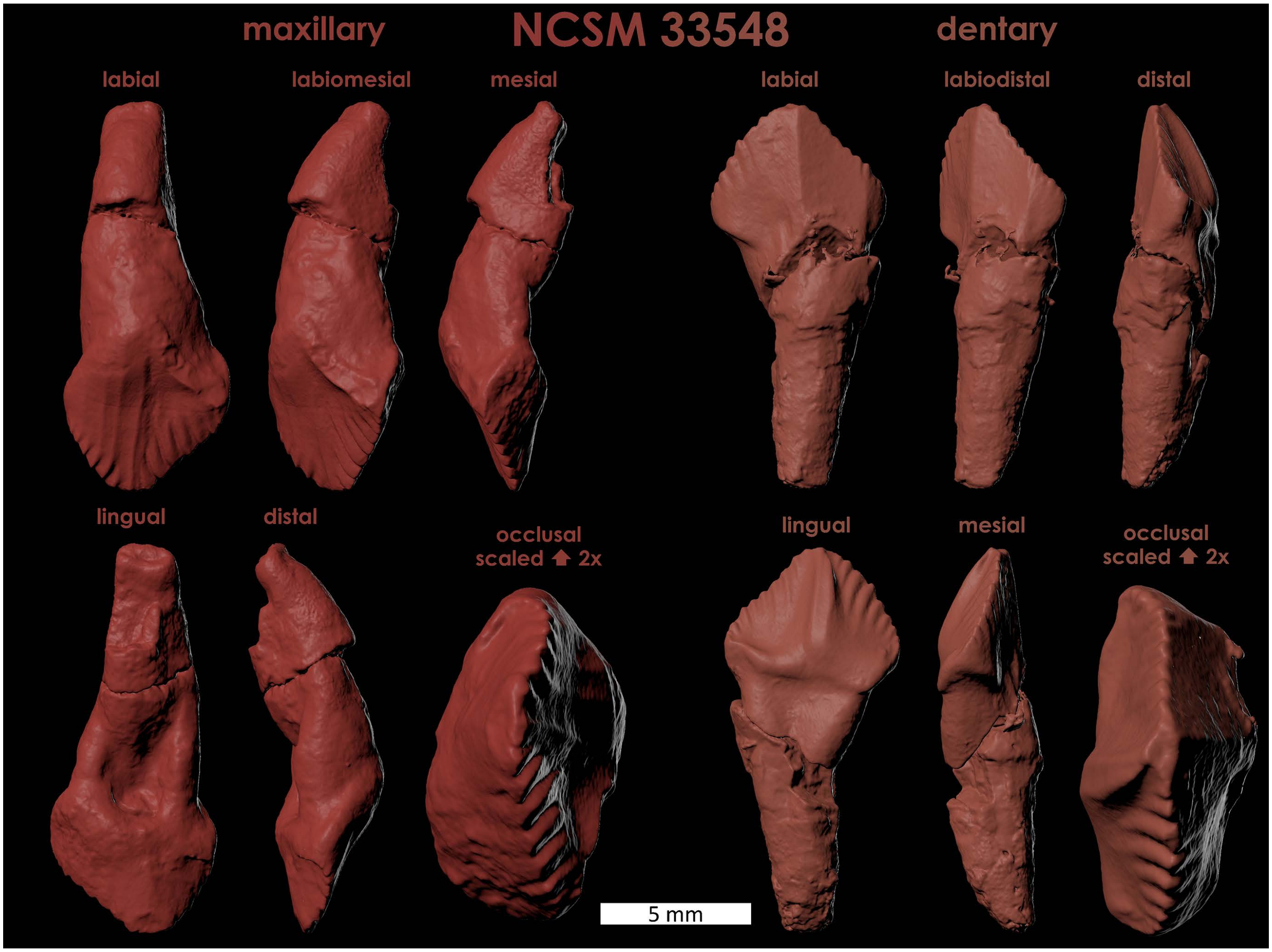

Fig 37. Maxillary and dentary dentation of *Fona herzogae.*

https://doi.org/10.1371/journal.pone.0353169.g037

Roots: Roots are straight to slightly curved, but not as curved as those of *Hypsilophodon* [7]. They taper basally, have circular to oval cross sections, and do not contact each other.

Wear facets: On NCSM 36130, distal crowns 10–13 are fully erupted, well-preserved, and display an interesting wear pattern (Fig 27A). Each crown has two vertical wear facets on the labial surface, in contrast to the continuous occlusal surface of the maxillary teeth. These facets form two flat surfaces, oriented apicobasally, that converge at a midline eminence, indicating that the labial surface of each dentary crown likely occluded with the lingual surfaces of two maxillary crowns during mastication. This wear pattern is also present on a *Fona* dentary from the Manolo locality (FMNH PR 4581). A somewhat similar pattern of wear is seen in the mesial teeth of *Th. neglectus* [18]. All wear facets seem to be planar and oriented nearly vertically, forming a 90-degree angle with the long axis of the tooth. This is in contrast to the more concave facets that are angled at 70° in *Changchunsaurus* [19]. Vertical wear facets are also present in the cheek teeth of *Manidens* [116].

The labiodistal wear facets of well-preserved crowns ten and 13 are limited to the apical half of the crown, whereas the labiomesial wear facet seems to continue basally. The labiodistal wear facet terminates abruptly, forming a flat but angled shelf that slants apically towards the midline eminence. Basal to this shelf, the remainder of the cingulum is minimally worn and appears as a bulbous inflation. A third possible mesiodistally flat wear facet runs apicobasally along the midline eminence of teeth 11 and 14, as in *Stegoceras* [107; fig 4G].

On NCSM 36131, wear appears more extensive. Crowns are apicobasally shorter, and wear extends down the entire length of the labial surface. There are no facets with abruptly terminating shelves, nor is there a clear distinction between mesial and distal wear surfaces. However, the basal-most part of the crown and cingulum along the midline remains minimally unworn relative to the rest of the labial surface.

Enamel: The distribution of enamel across the maxillary crowns is symmetrical, as revealed by histological sectioning of an isolated dentary tooth [119].

Discussion

The updated phylogenetic analysis, incorporating several targeted revisions to the matrix of Avrahami et al. [9], yields a number of notable results, most prominently improved resolution within Thescelosauridae. Several SBEDOs that were previously recovered in ambiguous or weakly supported positions within Thescelosauridae are now resolved as more stable members of Thescelosaurinae (Fig 38). Within Thescelosaurinae, taxa are arranged in an early branching to late-branching sequence beginning with a poorly supported clade comprising *Diluvicursor*, *Othnielosaurus*, and *Changmiania*. This assemblage is recovered as sister to a later-branching grouping containing *Changchunsaurus*, *Jeholosaurus*, *Haya*, and *Koreanosaurus*. *Fona* and *Oryctodromeus* form a clade that is recovered as the sister group to *Thescelosaurus* (including T. *neglectus, T. garbanii,* and *T. assiniboiensis*). The close and well-supported relationship between *Fona, Oryctodromeus,* and *Thescelosaurus* is consistent with previous analyses [9], whereas the stemward placement of *Changchunsaurus*, *Jeholosaurus*, *Haya*, and *Koreanosaurus* were previously recovered in less resolved or alternative positions within Thescelosauridae.

The time-calibrated Bayesian analysis (Fig 39) recovers a topology broadly congruent with the parsimony results in terms of thescelosaurine composition, but with subtle differences in branching order. In this analysis, *Jeholosaurus* and *Changchunsaurus* occupy successive stemward positions, followed by a clade uniting *Haya* and *Koreanosaurus*. Crownward of this grouping, *Fona* and *Oryctodromeus* form successive sister taxa to *Thescelosaurus* (*T. neglectus, T. garbanii* + *T. assiniboiensis*). A broadly similar arrangement was recovered in the Bayesian analyses of Avrahami et al. [9]; however, the inclusion of *Koreanosaurus* within Thescelosaurinae is further supported here, along with stronger posterior probabilities for the inclusion of *Jeholosaurus*, *Changchunsaurus,* and *Haya. Koreanosaurus* was independently recovered as a Thescelosaurinae by Jung et al. [120; supplementary information 1] following their own rescoring of *Koreanosaurus* within the Avrahami et al. [9] matrix.

The Bayesian analysis also differs from the parsimony analysis in that the inclusion of poorly supported wildcard taxa (e.g., *Othnielosaurus* and *Changmiania*) are excluded from Thescelosauridae and recovers *Diluvicursor* as the earliest diverging member of Orodrominae, albeit with weak node support (posterior probability = 0.18).

A particularly notable result of both analyses is the placement of *Koreanosaurus* within Thescelosaurinae, rather than within Orodrominae as recovered in prior parsimony and Bayesian analyses [9]. Following extensive character recoding based on firsthand examination and 3D surface data, *Koreanosaurus* is consistently resolved within Thescelosaurinae in both analytical frameworks. In the parsimony analysis, it is positioned between *Haya* and the clade formed by *Oryctodromeus* + *Fona*. In the Bayesian analysis, *Koreanosaurus* is recovered as the sister taxon to *Haya*, together forming a clade that is one node stemward of *Fona*. This placement receives strong node support in both analyses.

Our new topologies permit us to reevaluate synapomorphic character support for Orodrominae and Thescelosaurinae. Several synapomorphies distinguish Orodrominae and Thescelosaurinae and support their position within

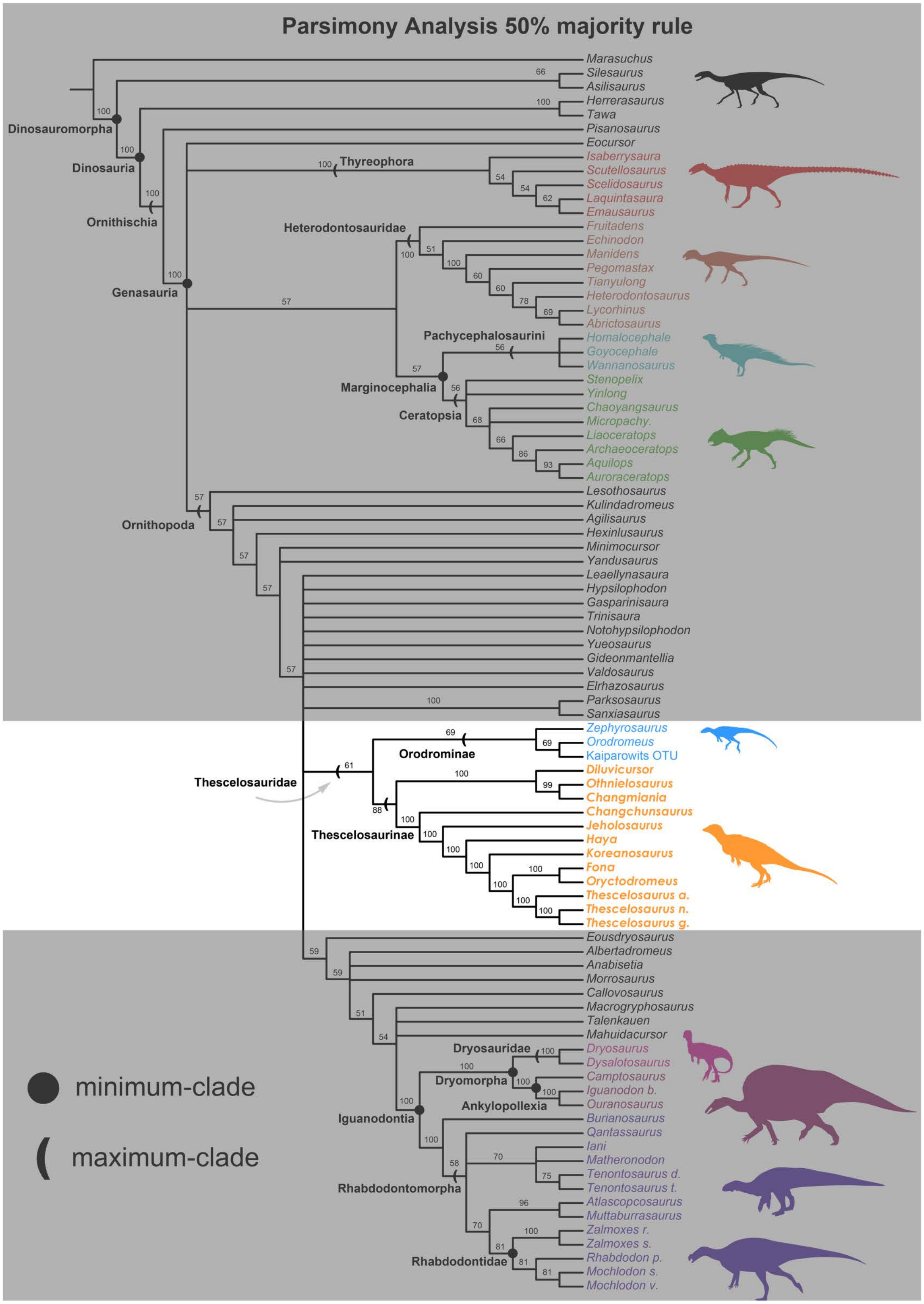

Fig 38. Phylogenetic relationships of early-diverging ornithischians with emphasis on Thescelosauridae. Strict parsimony analysis conducted in TNT using an updated version of the Avrahami et al. [9] character–taxon matrix, incorporating firsthand observation and recoding of *Koreanosaurus*.

Topology shown is the 50% majority-rule consensus of most parsimonious trees from a non-time-calibrated analysis. Numbers at nodes represent clade frequencies (%) across the pool of most parsimonious trees, indicating the proportion of equally optimal trees in which each clade occurs (not resampling or decay support values). Silhouettes representing Dinosauromorpha, Heterodontosauridae, Thyreophora, Ceratopsia, and Ankylopollexia by Scott Hartman; Pachycephalosaurini by Jack Mayer Wood; and Dryosauridae by Tasman Dixon, all downloaded from Phylopic.org.

https://doi.org/10.1371/journal.pone.0353169.g038

Thescelosauridae based on the topology recovered from the 50% majority rule parsimony tree (Fig 40). Although some of these character states also occur in other ornithischian taxa outside of Thescelosauridae (e.g., certain elasmarians and earlier diverging neornithischians), most are unambiguous in diagnosing the split between orodromines and thescelosaurines in the context of thescelosaurid interrelationships.

Orodrominae (represented here by *Zephyrosaurus*, the Kaiparowits orodromine OTU, *Albertadromeus*, and *Orodromeus*) is distinguished from Thescelosaurinae by the presence of a discrete jugal horn (char. 60:1), which unites all orodromines preserving a jugal (*Zephyrosaurus*, Kaiparowits orodromine OTU, *Orodromeus*). By contrast thescelosaurines lack a true horn. In thescelosaurines preserving this element, the jugal is either unornamented (*Thescelosaurus*, *Haya*) or bears only diffuse rugosity on the lateral surface (*Fona*, *Oryctodromeus*, *Changchunsaurus*, *Jeholosaurus*).

The shape of the rostral process of the jugal (char. 67:1) was coded in previous analyses as exhibiting a subrectangular lateral profile with approximately parallel dorsal and ventral margins in orodromines preserving this region (e.g., *Zephyrosaurus*, *Orodromeus*). In contrast, thescelosaurines preserving the rostral jugal (*Changchunsaurus, Jeholosaurus, Haya, Fona, Oryctodromeus*, and *Thescelosaurus*) were coded as exhibiting a condition in which the dorsal and ventral margins taper rostrally. However, although this character is recovered as phylogenetically informative in the present analyses based on these historical codings, firsthand comparisons suggest that the morphological distinction between orodromines and thescelosaurines is comparatively subtle and likely does not represent as discrete of a transformation as observed in more derived ornithopods such as *Tenontosaurus*, for which this character concept was likely originally developed. Accordingly, the phylogenetic utility and broader applicability of this character within Thescelosauridae may warrant future reassessment through expanded character revision and broader taxonomic sampling.

The basipterygoid processes (char. 172:1) are oriented ventrally in lateral view in orodromines preserving these structures (*Zephyrosaurus*, *Orodromeus*), but are oriented rostroventrally in all thescelosaurines in which this region is preserved (*Changchunsaurus*, *Jeholosaurus*, *Haya*, *Fona*, *Oryctodromeus*, *Thescelosaurus*).

Finally, a newly added character introduced in the present analysis, the presence of a distinct tuberosity on the medial surface of the scapular neck near the base of the blade (char. 410:1), unites all thescelosaurines preserving the scapula in medial view (*Koreanosaurus*, *Fona*, *Oryctodromeus*, *Thescelosaurus*). This feature is absent in all orodromines in which the medial scapular surface can be assessed (*Zephyrosaurus*, Kaiparowits orodromine OTU, *Orodromeus*).

When considered within a paleobiogeographic framework, this topology further supports a Jurassic or earliest Cretaceous Asian origin for Thescelosaurinae, followed by one or more dispersal events into North America by the mid-Cretaceous. In contrast, Orodrominae remains geographically restricted to North America throughout its known temporal range, persisting from the Early through earliest Late Cretaceous before disappearing prior to the latest Cretaceous. Notably, a recently described Korean thescelosaurid, *Doolysaurus huhmini* [120], was independently recovered near the base of Thescelosaurinae and in close proximity to *Fona herzogae* using a modified version of the matrix presented in Avrahami et al. (2024), while also recovering *Koreanosaurus* within Thescelosaurinae. Although *Doolysaurus* was published after completion of the present analyses and therefore could not be incorporated into the current dataset, its phylogenetic placement in Jung et al. [120] provides additional support for an Asian–North American distribution of Thescelosaurinae, contrasting with the exclusively North American distribution currently observed in Orodrominae.

It is important to emphasize that the goal of this analysis is not to broadly reassess ornithischian phylogeny, but rather to refine taxon placement and clade support specifically among thescelosaurine and orodromine SBEDOs. Recent studies

Time Calibrated Baysian Analysis, Maximum Clade Credibility Tree

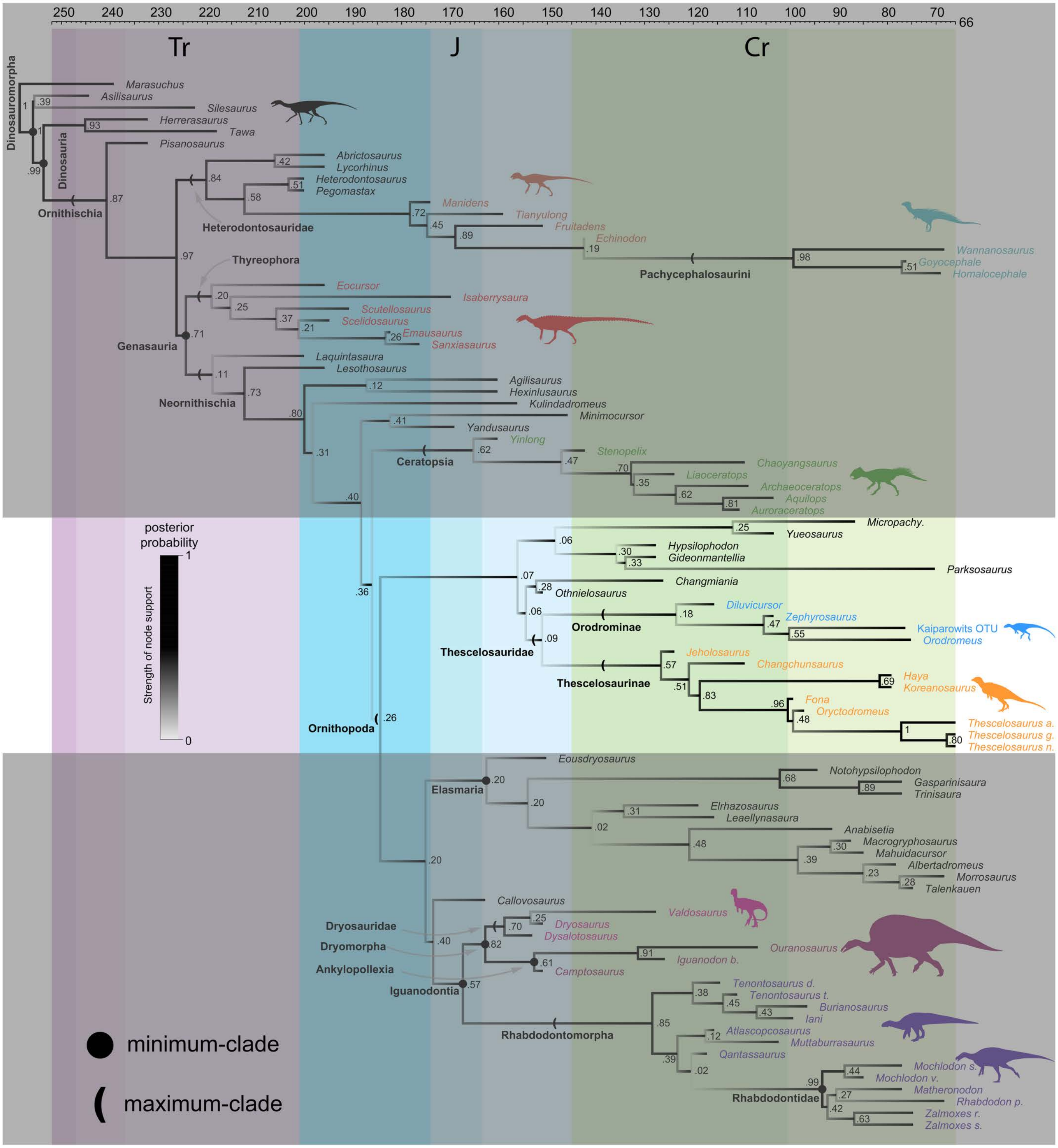

Fig 39. Phylogenetic relationships of early-diverging ornithischians with emphasis on Thescelosauridae inferred from a Bayesian time-calibrated analysis. The analysis employed an updated version of the Avrahami et al. (2024) character–taxon matrix, incorporating firsthand observation and recoding of *Koreanosaurus*. Topology shown is the maximum-clade credibility (MCC) tree derived from the posterior distribution of

time-calibrated trees. Branch lengths are proportional to time (Ma). Numbers at nodes indicate posterior probabilities. Major ornithischian clades are labeled for reference. Silhouettes obtained from PhyloPic.org. Dinosauromorpha, Heterodontosauridae, Thyreophora, Ceratopsia, and Ankylopollexia by Scott Hartman; Pachycephalosaurini by Jack Mayer Wood; and Dryosauridae by Tasman Dixon.

https://doi.org/10.1371/journal.pone.0353169.g039

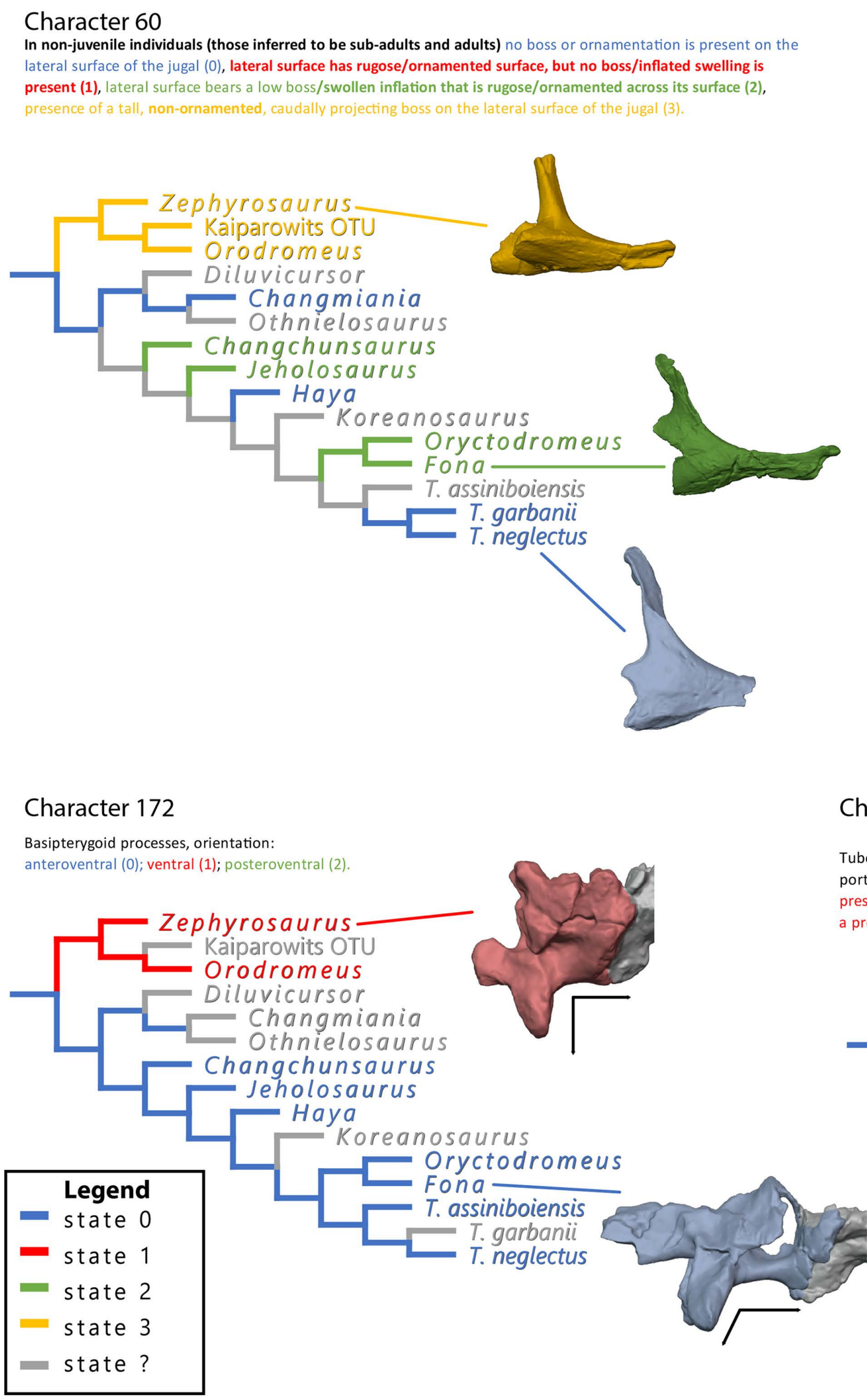

Fig 40. Selected synapomorphies diagnosing Orodrominae and Thescelosaurinae mapped onto the 50% majority-rule parsimony topology of Fig 38. Colored branches indicate mapped character states corresponding to those shown in the legend and in the text above each topology. Together, these characters diagnose the reciprocal monophyly of Orodrominae and Thescelosaurinae within Thescelosauridae in the context of the recovered parsimony topology. Character 67 is included as recovered in the present analysis based on historical codings; however, firsthand comparisons suggest that the morphological distinction between the orodromine and thescelosaurine conditions is subtle, and the phylogenetic utility of this character within Thescelosauridae should be reassessed in future character revision and broader taxonomic sampling.

https://doi.org/10.1371/journal.pone.0353169.g040

have recovered markedly disparate topologies [12,42,121,122], underscoring instability. Accordingly, the present analysis does not aim to evaluate topological changes among more distantly related outgroup clades (e.g., iguanodontians, elasmarians, marginocephalians, heterodontosaurids), but instead represents a targeted reassessment of thescelosaurids grounded in extensive firsthand observations, direct specimen documentation, and side-by-side comparisons using three-dimensional models. Future work will further refine these relationships through the inclusion of newly described orodromine material, direct observation and recoding of non-North American SBEDOs, and the integration of new taxa and characters introduced in recent studies [12,42,121,122]. Nonetheless, within the scope of thescelosaurine and orodromine SBEDOs, the results presented here provide the most robustly supported phylogenetic framework currently available.

Conclusion

Fona herzogae represents one of the most complete and extensively sampled cranial datasets currently known for a small-bodied early diverging ornithischian and provides an important anatomical framework for understanding thescelosaurid evolution. The abundance of disarticulated cranial material from multiple individuals permits detailed reconstruction and documentation of individual skull elements from multiple orientations, substantially expanding the known cranial anatomy of *Fona* and refining the interpretation of phylogenetically informative cranial morphologies across Thescelosauridae.

The revised phylogenetic analyses presented here recovers a more stable and well supported Thescelosaurinae clade, including the placement of *Koreanosaurus* within the clade in both parsimony and Bayesian frameworks. The recovered topologies further support an Asian component to thescelosaurine evolution, with taxa such as *Jeholosaurus, Changchunsaurus, Haya,* and *Koreanosaurus* reinforcing a broader Asian–North American distribution for Thescelosaurinae. In contrast, Orodrominae remains geographically restricted to North America throughout its known evolutionary history. Together, these results suggest that important components of thescelosaurine diversification and cranial evolution occurred in Asia prior to one or more dispersal events into North America during the Cretaceous.

More broadly, the present study highlights how historically underdescribed cranial material, combined with modern digital visualization and targeted phylogenetic revision, can substantially improve character interpretation and clade resolution among early diverging ornithischians. Future studies should further explore intraspecific variation, ontogeny, and histological development within *Fona* and other contemporaneous SBEDOs in order to better understand the role of morphological variability in shaping evolutionary trajectories, ecological interactions, and taxonomic diversity within Dinosauria.

Supporting information

S1.01 File. High-resolution images of the premaxilla. Compressed archive containing high-resolution image files of premaxillary elements described in this study.
(7Z)

S1.02 File. High-resolution images of the maxilla. Compressed archive containing high-resolution image files of maxillary elements described in this study.
(7Z)

S1.03 File. High-resolution images of the nasal. Compressed archive containing high-resolution image files of nasal elements described in this study.
(7Z)

S1.04 File. High-resolution images of the jugal. Compressed archive containing high-resolution image files of jugal elements described in this study.
(7Z)

S1.05 File. High-resolution images of the quadratojugal. Compressed archive containing high-resolution image files of quadratojugal elements described in this study.
(7Z)

S1.06 File. High-resolution images of the postorbital. Compressed archive containing high-resolution image files of postorbital elements described in this study.
(7Z)

S1.07 File. High-resolution images of the quadrate. Compressed archive containing high-resolution image files of quadrate elements described in this study.
(7Z)

S1.08 File. High-resolution images of the squamosal. Compressed archive containing high-resolution image files of squamosal elements described in this study.
(7Z)

S1.09 File. High-resolution images of the parietal. Compressed archive containing high-resolution image files of parietal elements described in this study.
(7Z)

S1.10 File. High-resolution images of the frontal. Compressed archive containing high-resolution image files of frontal elements described in this study.
(7Z)

S1.11 File. High-resolution images of the prefrontal. Compressed archive containing high-resolution image files of prefrontal elements described in this study.
(7Z)

S1.12 File. High-resolution images of the lacrimal. Compressed archive containing high-resolution image files of lacrimal elements described in this study.
(7Z)

S1.13 File. High-resolution images of the supraorbital. Compressed archive containing high-resolution image files of supraorbital elements described in this study.
(7Z)

S1.14 File. High-resolution images of the supraoccipital. Compressed archive containing high-resolution image files of supraoccipital elements described in this study.
(7Z)

S1.15 File. High-resolution images of the otoccipital. Compressed archive containing high-resolution image files of otoccipital elements described in this study.
(7Z)

S1.16 File. High-resolution images of the prootic. Compressed archive containing high-resolution image files of prootic elements described in this study.
(7Z)

S1.17 File. High-resolution images of the basisphenoid. Compressed archive containing high-resolution image files of basisphenoid elements described in this study.
(7Z)

S1.18 File. High-resolution images of the pterygoid. Compressed archive containing high-resolution image files of pterygoid elements described in this study.
(7Z)

S1.19 File. High-resolution images of the ectopterygoid. Compressed archive containing high-resolution image files of ectopterygoid elements described in this study.
(7Z)

S1.20 File. High-resolution images of the dentary. Compressed archive containing high-resolution image files of dentary elements described in this study.
(7Z)

S1.21 File. High-resolution images of the surangular. Compressed archive containing high-resolution image files of surangular elements described in this study.
(7Z)

S1.22 File. High-resolution images of the angular. Compressed archive containing high-resolution image files of angular elements described in this study.
(7Z)

S1.23 File. High-resolution images of the splenial. Compressed archive containing high-resolution image files of splenial elements described in this study.
(7Z)

S1.24 File. High-resolution images of the premaxillary dentition of NCSM 33548. Compressed archive containing high-resolution image files of the premaxillary dentition of NCSM 33548.
(7Z)

S1.25 File. High-resolution images of the premaxillary dentition of NCSM 35571. Compressed archive containing high-resolution image files of the premaxillary dentition of NCSM 35571.
(7Z)

S1.26 File. High-resolution images of the premaxillary dentition of NCSM 36178. Compressed archive containing high-resolution image files of the premaxillary dentition of NCSM 36178.
(7Z)

S1.27 File. High-resolution images of the premaxillary dentition of NCSM 36180. Compressed archive containing high-resolution image files of the premaxillary dentition of NCSM 36180.
(7Z)

S1.28 File. High-resolution images of the premaxillary dentition of NCSM 36181. Compressed archive containing high-resolution image files of the premaxillary dentition of NCSM 36181.
(7Z)

S1.29 File. High-resolution images of the premaxillary dentition of NCSM 36184. Compressed archive containing high-resolution image files of the premaxillary dentition of NCSM 36184.
(7Z)

S1.30 File. High-resolution images of the premaxillary dentition of NCSM 36188. Compressed archive containing high-resolution image files of the premaxillary dentition of NCSM 36188.
(7Z)

S1.31 File. High-resolution images of the premaxillary dentition of NCSM 36190. Compressed archive containing high-resolution image files of the premaxillary dentition of NCSM 36190.
(7Z)

S1.32 File. High-resolution images of the right maxillary dentition of NCSM 36183. Compressed archive containing high-resolution image files of the right maxillary dentition of NCSM 36183.
(7Z)

S1.33 File. High-resolution images of the left maxillary dentition of NCSM 36185. Compressed archive containing high-resolution image files of the left maxillary dentition of NCSM 36185.
(7Z)

S1.34 File. High-resolution images of the right maxillary dentition of NCSM 36186. Compressed archive containing high-resolution image files of the right maxillary dentition of NCSM 36186.
(7Z)

S1.35 File. High-resolution images of the dentary dentition of NCSM 36179. Compressed archive containing high-resolution image files of the dentary dentition of NCSM 36179.
(7Z)

S1.36 File. High-resolution images of the dentary dentition of NCSM 36182. Compressed archive containing high-resolution image files of the dentary dentition of NCSM 36182.
(7Z)

S1.37 File. High-resolution images of the dentary dentition of NCSM 36189. Compressed archive containing high-resolution image files of the dentary dentition of NCSM 36189.
(7Z)

S1.38 File. Phylogenetic matrix changes relative to Avrahami et al. 2024. Document summarizing changes made to the phylogenetic matrix used in this study, including character additions, rescoring, and relevant comments.
(DOCX)

S1.39 File. Updated phylogenetic matrix with cell comments. Excel spreadsheet containing the updated phylogenetic matrix, with cell-level comments documenting scoring changes.
(XLSX)

S1.40 File. Phylogenetic matrix for parsimony analysis. TNT file used for the parsimony phylogenetic analysis presented in this study.
(TXT)

Acknowledgments

We are especially grateful to **L. Herzog** for the discovery of the Mini Troll locality, for which *Fona* is named, and for her continued assistance with specimen management, preparation, and CT scanning throughout this project. We thank **A. Giterman** for preparation of *Fona* specimens from the Mini Troll, Last Chance Theropod, Magic Man, and Karmic localities. **A. Knowles** prepared the complete articulated postcranial skeleton of NCSM 33548. We also thank **E. Lund** for preparation of additional Mini Troll material, **A. Shinya** for preparation of FMNH PR 4581, and **F. Gallastegui** for the discovery of FMNH PR 4581.

We acknowledge **S. Moran** and **W. Simpson** for cataloging and assigning institutional numbers to all NCSM and FMNH specimens. We are deeply appreciative of the many NCSM, NCSU, and FMNH volunteers who contributed to the

collection, preparation, and digitization of these materials, including **E. Bender, H. Miller, C. Hauser, K. Lindsay, B. Dale, E. Schubarg, A. Moore, H. Orlowski,** and **V. Harpe**.

We are grateful to the reviewers **D. Barta, F. Han,** and **Z. Hannebaum**. We also thank **D. Varricchio, E. Metz, J. Scannella,** and **E. Lamm** for facilitating access to and examination of comparative material of *Orodromeus* and *Oryctodromeus* at the Museum of the Rockies. We are further indebted to the curators and collections staff who supported additional institutional visits: **B. Peecook** (IMNH), **C. Kammerer** (NCSM), **T. Scheyer** and **D. Hansen** (NMZ), **Y. Schicker-Siber** (SMA), **J. Sertich** (DMNS), **R. Cifelli** and **J. Larsen** (OMNH), **D. Evans** and **B. Iwama** (ROM), **S. Johnston** and **C. Byrd** (MCZ), **J. Kirkland** and **M. Hayden** (UGS), **R. Irmis** (NHMU), **V. Rhue** and **D. Brinkman** (YPM), **M. Jin** and **C. Mehling** (AMNH), **L. Jacobs** and **D. Winkler** (SMU), **R. Scheetz** (BYU), **A. Millhouse** (NMNH), **P. Barrett, S. Maidment, M. Jones,** and **M. Day** (NHMUK), and **X. Zheng** (STMN). We also thank **Min Huh, Jungyun Jung, Hyemin Jo,** and **Minguk Kim** for hosting the first author during a valuable research visit to Gwangju, South Korea, to study *Koreanosaurus*.

We are particularly indebted to **P.J. Makovicky**, whose prior collaboration and continued engagement have substantially shaped this research. He led the FMNH field team that discovered the Manolo locality specimen, generously provided the three-dimensional scans of the cranial elements analyzed here, and contributed foundational insights that informed both this study and earlier related work.

We thank the staff of the Bureau of Land Management Utah and the Price Field Office for permitting support. All necessary permits were obtained for the described study, which complied with all relevant regulations.

Author contributions

Conceptualization: Haviv M. Avrahami, Lindsay E. Zanno.

Data curation: Haviv M. Avrahami, Lindsay E. Zanno.

Formal analysis: Haviv M. Avrahami, Lindsay E. Zanno.

Funding acquisition: Haviv M. Avrahami, Lindsay E. Zanno.

Investigation: Haviv M. Avrahami, Lindsay E. Zanno.

Methodology: Haviv M. Avrahami, Lindsay E. Zanno.

Project administration: Lindsay E. Zanno.

Resources: Lindsay E. Zanno.

Software: Haviv M. Avrahami.

Supervision: Lindsay E. Zanno.

Validation: Lindsay E. Zanno.

Visualization: Haviv M. Avrahami.

Writing – original draft: Haviv M. Avrahami.

Writing – review & editing: Lindsay E. Zanno.

References

1. Zanno LE, Gates TA, Avrahami HM, Tucker RT, Makovicky PJ. An early-diverging iguanodontian (Dinosauria: Rhabdodontomorpha) from the Late Cretaceous of North America. PLoS One. 2023;18(6):e0286042. https://doi.org/10.1371/journal.pone.0286042 PMID: 37285376
2. Becerra MG, Pol D, Porro LB, Paulina-Carabajal A, Rauhut OWM. Craniomandibular osteology of *Manidens condorensis* (Ornithischia: Heterodontosauridae) from the upper Lower Jurassic of Argentina. Journal of Vertebrate Paleontology. 2022;42(3). https://doi.org/10.1080/02724634.2023.2181087
3. Porro LB, Witmer LM, Barrett PM. Digital preparation and osteology of the skull of *Lesothosaurus diagnosticus* (Ornithischia: Dinosauria). PeerJ. 2015;3:e1494. https://doi.org/10.7717/peerj.1494 PMID: 26713245

4. Thomas DA. The cranial anatomy of *Tenontosaurus tilletti* Ostrom, 1970 (Dinosauria, Ornithopoda). Palaeontologia Electronica. 2015;18(2).
5. Avrahami HM, Lindsey KP, Herzog L. Advances in 3D Surface Scanning: Mobile Device Applications Set a New Standard for the Digitization of Fossils and Museum Artifacts. Journal of Vertebrate Paleontology. 2023;77(83):83.
6. Huxley TH. On *Hypsilophodon foxii*, a new dinosaurian from the Wealden of the Isle of Wight. QJGS. 1870;26(1–2):3–12. https://doi.org/10.1144/gsl.jgs.1870.026.01-02.07
7. Galton PM. The ornithischian dinosaur *Hypsilophodon* from the Wealden of the Isle of Wight. Bulletin of The British Museum (Natural History). 1974;25(1).
8. Bertozzo F, Kecheng N, Gillette NV, Godefroit P. Anatomical description and digital reconstruction of the skull of Jeholosaurus shangyuanensis (Dinosauria, Ornithopoda) from China. PLoS One. 2025;20(1):e0312519. https://doi.org/10.1371/journal.pone.0312519 PMID: 39854443
9. Avrahami HM, Makovicky PJ, Tucker RT, Zanno LE. A new semi-fossorial thescelosaurine dinosaur from the Cenomanian-age Mussentuchit Member of the Cedar Mountain Formation, Utah. Anat Rec (Hoboken). 2024;307(12):3717–81. https://doi.org/10.1002/ar.25505 PMID: 38979930
10. Button DJ, Porro LB, Lautenschlager S, Jones MEH, Barrett PM. Multiple pathways to herbivory underpinned deep divergences in ornithischian evolution. Curr Biol. 2023;33(3):557-565.e7. https://doi.org/10.1016/j.cub.2022.12.019 PMID: 36603586
11. Debraga M, Rybczynski N, Reisz R. A methodology for skull reconstruction. Comptes Rendus Palevol. 2024;23(9). https://doi.org/10.5852/cr-palevol2024v23a9
12. Fonseca AO, Reid IJ, Venner A, Duncan RJ, Garcia MS, Müller RT. A comprehensive phylogenetic analysis on early ornithischian evolution. Journal of Systematic Palaeontology. 2024;22(1). https://doi.org/10.1080/14772019.2024.2346577
13. Goloboff PA, Morales ME. TNT version 1.6, with a graphical interface for MacOS and Linux, including new routines in parallel. Cladistics. 2023;39(2):144–53. https://doi.org/10.1111/cla.12524 PMID: 36682054
14. Bouckaert R, Vaughan TG, Barido-Sottani J, Duchêne S, Fourment M, Gavryushkina A, et al. BEAST 2.5: An advanced software platform for Bayesian evolutionary analysis. PLoS Comput Biol. 2019;15(4):e1006650. https://doi.org/10.1371/journal.pcbi.1006650 PMID: 30958812
15. Rambaut A, Drummond AJ, Xie D, Baele G, Suchard MA. Posterior Summarization in Bayesian Phylogenetics Using Tracer 1.7. Syst Biol. 2018;67(5):901–4. https://doi.org/10.1093/sysbio/syy032 PMID: 29718447
16. Ostrom JH. A new species of hadrosaurian dinosaur from the Cretaceous of New Mexico. Journal of Paleontology Memoir. 1961;35(3):575–7.
17. Nabavizadeh A, Weishampel DB. The Predentary Bone and Its Significance in the Evolution of Feeding Mechanisms in Ornithischian Dinosaurs. Anat Rec (Hoboken). 2016;299(10):1358–88. https://doi.org/10.1002/ar.23455 PMID: 27490958
18. Boyd CA. The cranial anatomy of the neornithischian dinosaur *Thescelosaurus neglectu*s. PeerJ. 2014;2:e669. https://doi.org/10.7717/peerj.669 PMID: 25405076
19. Liyong J, Jun C, Shuqin Z, Butler RJ, Godefroit P. Cranial anatomy of the small ornithischian dinosaur *Changchunsaurus parvus* from the Quantou Formation (Cretaceous: Aptian–Cenomanian) of Jilin Province, northeastern China. Journal of Vertebrate Paleontology. 2010;30(1):196–214. https://doi.org/10.1080/02724630903412372
20. Krumenacker L. Osteology, phylogeny, taphonomy, and ontogenetic histology of *Oryctodromeus cubicularis*, from the Middle Cretaceous (Albian-Cenomanian) of Montana and Idaho. Montana State University. 2017.
21. Sues HD. Anatomy and relationships of a new hypsilophodontid dinosaur from the Lower Cretaceous of North America. Palaeontographica Abt A. 1980;169:51–72.
22. Barrett PM, Han FL. Cranial anatomy of *Jeholosaurus shangyuanensis* (Dinosauria: Ornithischia) from the Early Cretaceous of China. Zootaxa. 2009;2072(1):31–55.
23. Yang Y, Wu W, Dieudonné P-E, Godefroit P. A new basal ornithopod dinosaur from the Lower Cretaceous of China. PeerJ. 2020;8:e9832. https://doi.org/10.7717/peerj.9832 PMID: 33194351
24. Sereno PC. *Lesothosaurus*, "fabrosaurids," and the early evolution of Ornithischia. Journal of Vertebrate Paleontology. 1991;11:168–97.
25. Barta DE, Norell MA. The osteology of *Haya griva* (Dinosauria: Ornithischia) from the Late Cretaceous of Mongolia. Bulletin of the American Museum of Natural History. 2021;445(1):1–112.
26. Hannebaum ZJD. Exploring orodromine variation in the Two Medicine-Judith River-Belly River clastic wedge in Montana and Alberta. Bozeman: Montana State University. 2025.
27. Farke AA, Maxwell WD, Cifelli RL, Wedel MJ. A Ceratopsian Dinosaur from the Lower Cretaceous of Western North America, and the Biogeography of Neoceratopsia. PLoS One. 2014;9(12):e112055. https://doi.org/10.1371/journal.pone.0112055 PMID: 25494182
28. Makovicky PJ, Kilbourne BM, Sadleir RW, Norell MA. A new basal ornithopod (Dinosauria, Ornithischia) from the Late Cretaceous of Mongolia. Journal of Vertebrate Paleontology. 2011;31(3):626–40. https://doi.org/10.1080/02724634.2011.557114
29. Scheetz RD. Osteology of *Orodromeus makelai* and the phylogeny of basal ornithopod dinosaurs. Citeseer. 1999.
30. Salgado L, Canudo JI, Garrido AC, Moreno-Azanza M, Martínez LCA, Coria RA, et al. A new primitive Neornithischian dinosaur from the Jurassic of Patagonia with gut contents. Sci Rep. 2017;7:42778. https://doi.org/10.1038/srep42778 PMID: 28202910
31. Sereno PC, Zhimin D. The skull of the basal stegosaur *Huayangosaurus taibaii* and a cladistic diagnosis of stegosauria. Journal of Vertebrate Paleontology. 1992;12(3):318–43. https://doi.org/10.1080/02724634.1992.10011463

32. Herrera-Castillo CM, Carrillo-Briceño JD, Sánchez-Villagra MR. Non-invasive imaging reveals new cranial element of the basal ornithischian dinosaur Laquintasaura venezuelae, Early Jurassic of Venezuela. Anartia. 2021;:53–60.

33. Breeden BT III, Rowe TB. New Specimens of *Scutellosaurus lawleri* Colbert, 1981, from the Lower Jurassic Kayenta Formation in Arizona Elucidate the Early Evolution of Thyreophoran Dinosaurs. Journal of Vertebrate Paleontology. 2020;40(4):e1791894. https://doi.org/10.1080/02724634.2020.1791894

34. Yu C, Prieto-Marquez A, Chinzorig T, Badamkhatan Z, Norell M. A neoceratopsian dinosaur from the early Cretaceous of Mongolia and the early evolution of ceratopsia. Commun Biol. 2020;3(1):499. https://doi.org/10.1038/s42003-020-01222-7 PMID: 32913206

35. Andrzejewski KA, Winkler DA, Jacobs LL. A new basal ornithopod (Dinosauria: Ornithischia) from the Early Cretaceous of Texas. PLoS One. 2019;14(3):e0207935. https://doi.org/10.1371/journal.pone.0207935 PMID: 30860999

36. Norman DB, Crompton AW, Butler RJ, Porro LB, Charig AJ. The Lower Jurassic ornithischian dinosaur *Heterodontosaurus tucki* Crompton & Charig, 1962: cranial anatomy, functional morphology, taxonomy, and relationships. Zoological Journal of the Linnean Society. 2011;:no-no. https://doi.org/10.1111/j.1096-3642.2011.00697.x

37. Rozadilla S, Agnolín FL, Novas FE. Osteology of the Patagonian ornithopodTalenkauen santacrucensis(Dinosauria, Ornithischia). Journal of Systematic Palaeontology. 2019;17(24):2043–89. https://doi.org/10.1080/14772019.2019.1582562

38. Zheng X-T, You H-L, Xu X, Dong Z-M. An Early Cretaceous heterodontosaurid dinosaur with filamentous integumentary structures. Nature. 2009;458(7236):333–6. https://doi.org/10.1038/nature07856 PMID: 19295609

39. Norman DB. Basal Iguanodontia. In: Weishampel DB, Dodson P, Osmólska H, editors. The Dinosauria, Second Edition. Berkeley: University of California Press. 2004. p. 413–37.

40. Weishampel DB, Jianu C, Csiki Z, Norman DB. Osteology and phylogeny of *Zalmoxes* (n. g.), an unusual Euornithopod dinosaur from the latest Cretaceous of Romania. Journal of Systematic Palaeontology. 2003;1(2):65–123. https://doi.org/10.1017/s1477201903001032

41. Barker CT, Naish D, Newham E, Katsamenis OL, Dyke G. Complex neuroanatomy in the rostrum of the Isle of Wight theropod *Neovenator salerii*. Sci Rep. 2017;7(1):3749. https://doi.org/10.1038/s41598-017-03671-3 PMID: 28623335

42. Dieudonné P, Becerra MG, Zanesco T, Tortosa T, Cruzado-Caballero P, Stein K, et al. Foskeia pelendonum, a new rhabdodontomorph from the Lower Cretaceous of Salas de los Infantes (Burgos Province, Spain), and a new phylogeny of ornithischian dinosaurs. Papers in Palaeontology. 2026;12(1). https://doi.org/10.1002/spp2.70057

43. Herne MC, Nair JP, Evans AR, Tait AM. New small-bodied ornithopods (Dinosauria, Neornithischia) from the Early Cretaceous Wonthaggi Formation (Strzelecki Group) of the Australian-Antarctic rift system, with revision of *Qantassaurus intrepidus* Rich and Vickers-Rich, 1999. J Paleontol. 2019;93(3):543–84. https://doi.org/10.1017/jpa.2018.95

44. Godefroit P, Sinitsa SM, Dhouailly D, Bolotsky YL, Sizov AV, McNamara ME, et al. Dinosaur evolution. A Jurassic ornithischian dinosaur from Siberia with both feathers and scales. Science. 2014;345(6195):451–5. https://doi.org/10.1126/science.1253351 PMID: 25061209

45. Knoll F. Nearly complete skull of *Lesothosaurus* (Dinosauria: Ornithischia) from the Upper Elliot Formation (Lower Jurassic: Hettangian) of Lesotho. Journal of Vertebrate Paleontology. 2002;22(2):238–43. https://doi.org/10.1671/0272-4634(2002)022[0238:ncsold]2.0.co;2

46. Barrett PM, Butler RJ, Knoll F. Small-bodied ornithischian dinosaurs from the Middle Jurassic of Sichuan, China. Journal of Vertebrate Paleontology. 2005;25(4):823–34. https://doi.org/10.1671/0272-4634(2005)025[0823:sodftm]2.0.co;2

47. Galton PM. The cranial anatomy of Dryosaurus, a hypsilophodontid dinosaur from the Upper Jurassic of North America and East Africa, with a review of hypsilophodontids from the Upper Jurassic of North America. Geologica et Palaeontologica. 1983;17:207–43.

48. Peng G. Jurassic ornithopod *Agilisaurus louderbacki* (Ornithopoda: Fabrosauridae) from Zigong, Sichuan, China. Vertebrata PalAsiatica. 1992;30(1):39–51.

49. Han F-L, Forster CA, Clark JM, Xu X. Cranial anatomy of *Yinlong downsi* (Ornithischia: Ceratopsia) from the Upper Jurassic Shishugou Formation of Xinjiang, China. Journal of Vertebrate Paleontology. 2015;36(1):e1029579. https://doi.org/10.1080/02724634.2015.1029579

50. Xu X, Makovicky PJ, Wang Xl X, Norell MA, You Hl H. A ceratopsian dinosaur from China and the early evolution of Ceratopsia. Nature. 2002;416(6878):314–7. https://doi.org/10.1038/416314a PMID: 11907575

51. Avrahami HM, Hauser CM, Zanno LE. New additions to the early diverging ornithischian *Zephyrosaurs shaffi* from the Cretaceous Cloverly Formation of North America. Journal of Vertebrate Paleontology. 2023;77(83):83.

52. Sereno PC. Taxonomy, morphology, masticatory function and phylogeny of heterodontosaurid dinosaurs. Zookeys. 2012;(226):1–225. https://doi.org/10.3897/zookeys.223.2840 PMID: 23166462

53. Weishampel DB, Witmer LM. Heterodontosauridae. In: Weishampel DB, Dodson P, Osmólska H, editors. The Dinosauria. Berkeley: University of California Press. 1990. p. 487–97.

54. Pol D, Rauhut OWM, Becerra M. A Middle Jurassic heterodontosaurid dinosaur from Patagonia and the evolution of heterodontosaurids. Naturwissenschaften. 2011;98(5):369–79. https://doi.org/10.1007/s00114-011-0780-5 PMID: 21452054

55. Bell PR, Herne MC, Brougham T, Smith ET. Ornithopod diversity in the Griman Creek Formation (Cenomanian), New South Wales, Australia. PeerJ. 2018;6:e6008. https://doi.org/10.7717/peerj.6008 PMID: 30533306

56. Coria RA, Salgado L. A basal iguanodontian (Ornithischia: Ornithopoda) from the Late Cretaceous of South America. Journal of Vertebrate Paleontology. 1996;16(3):445–57. https://doi.org/10.1080/02724634.1996.10011333

57. Zan S, Chen J, Jin L, Li T. A primitive ornithopod from the Early Cretaceous Quantou Formation of central Jilin, China. Vertebrata Pal Asiatica. 2005;43(3):182–93.

58. Takasaki R, Fiorillo AR, Tykoski RS, Kobayashi Y. Re-examination of the cranial osteology of the Arctic Alaskan hadrosaurine with implications for its taxonomic status. PLoS One. 2020;15(5):e0232410. https://doi.org/10.1371/journal.pone.0232410 PMID: 32374777

59. Galton PM. *Hypsilophodon foxii* and other smaller bipedal ornithischian dinosaurs from the Lower Cretaceous of southern England. Bernissart dinosaurs and Early Cretaceous terrestrial ecosystems. Bloomington: Indiana University Press. 2012. p. 225–81.

60. Brown CM, Boyd CA, Russel AP. A new basal ornithopod dinosaur (Frenchman Formation, Saskatchewan, Canada), and implications for late *Maastrichtian ornithischian* diversity in North America. Zoological Journal of the Linnean Society. 2011;163(4):1157–98. https://doi.org/10.1111/j.1096-3642.2011.00735.x

61. Brown CM, Evans DC, Ryan MJ, Russell AP. New data on the diversity and abundance of small-bodied ornithopods (Dinosauria, Ornithischia) from the Belly River Group (Campanian) of Alberta. Journal of Vertebrate Paleontology. 2013;33(3):495–520. https://doi.org/10.1080/02724634.2013.746229

62. Dunfee DR, Ridgely RC, Lamanna MC, Witmer LM. Ontogenetic analysis of the skull of the early-diverging iguanodontian *Dryosaurus elderae* (Dinosauria: Ornithopoda) from the Upper Jurassic Morrison Formation of Utah: new insights from microcomputed tomography. Journal of Vertebrate Paleontology. 2020;80:127.

63. Sullivan RM. A taxonomic review of the Pachycephalosauridae (Dinosauria: Ornithischia). New Mexico Museum of Natural History and Science Bulletin. 2006;35:347–65.

64. Morschhauser EM, Li D, You H, Dodson P. Cranial anatomy of the basal neoceratopsian *Auroraceratops rugosus* (Ornithischia: Ceratopsia) from the Yujingzi Basin, Gansu Province, China. Journal of Vertebrate Paleontology. 2018;38(sup1):36–68. https://doi.org/10.1080/02724634.2017.1399136

65. Carpenter K, Galton P. A photo documentation of bipedal ornithischian dinosaurs from the Upper Jurassic Morrison Formation, USA. GIW. 2018;5:167–207. https://doi.org/10.31711/giw.v5.pp167-207

66. He X, Cai K. A new species of *Yandusaurus* (Hypsilophodont dinosaur) from the Middle Jurassic of Dashanpu, Zigong, Sichuan. Journal of the Chengdu College of Geology Supplement. 1983;1:14–25.

67. Sobral G, Hipsley CA, Müller J. Braincase redescription of *Dysalotosaurus lettowvorbecki* (Dinosauria, Ornithopoda) based on computed tomography. Journal of Vertebrate Paleontology. 2012;32(5):1090–102. https://doi.org/10.1080/02724634.2012.693554

68. Button DJ, Zanno LE. Neuroanatomy of the late Cretaceous *Thescelosaurus neglectus* (Neornithischia: Thescelosauridae) reveals novel ecological specialisations within Dinosauria. Sci Rep. 2023;13(1):19224. https://doi.org/10.1038/s41598-023-45658-3 PMID: 37932280

69. Galton PM. Crania and endocranial casts from ornithopod dinosaurs of the families Dryosauridae and Hypsilophodentidae (Reptilia: Ornithischia). Geologica et Palaeontologica. 1989;23:217–39.

70. Rich TH, Galton PM, Vickers-rich P. The holotype individual of the ornithopod dinosaur *Leaellynasaura amicagraphica* Rich & Rich, 1989 (late Early Cretaceous, Victoria, Australia). Alcheringa: An Australasian Journal of Palaeontology. 2010;34(3):385–96. https://doi.org/10.1080/03115518.2010.489404

71. Godefroit P, Codrea V, Weishampel DB. Osteology of *Zalmoxes shqiperorum* (Dinosauria, Ornithopoda), based on new specimens from the Upper Cretaceous of Nălaţ-Vad (Romania). Geodiversitas. 2009;31(3):525–53. https://doi.org/10.5252/g2009n3a3

72. Bartholomai A, Molnar R. Muttaburrasaurus, a new iguanodontid (Ornithischia: Ornithopoda) dinosaur from the Lower Cretaceous of Queensland. 1981.

73. Maidment SCR, Porro LB. Homology of the palpebral and origin of supraorbital ossifications in ornithischian dinosaurs. LET. 2010;43(1):95–111. https://doi.org/10.1111/j.1502-3931.2009.00172.x

74. Boyd CA. The systematic relationships and biogeographic history of ornithischian dinosaurs. PeerJ. 2015;3:e1523. https://doi.org/10.7717/peerj.1523 PMID: 26713260

75. Gates TA, Lund EK, Boyd C, DeBlieux DD, Titus AL, Evans DC, et al. Ornithopod dinosaurs from the Grand Staircase-Escalante National Monument Region, Utah, and their role in paleobiogeographic and macroevolutionary studies. At the top of the Grand Staircase: the Late Cretaceous in Utah. 2013. p. 463–81.

76. You HL, Dodson P. Redescription of neoceratopsian dinosaur *Archaeoceratops* and early evolution of Neoceratopsia. Palaeontologica Polonica. 2003;48(2):261–72.

77. Norman DB, Witmer LM, Weishampel DB. Basal ornithischia. In: Weishampel DB, Dodson P, Osmólska H, editors. The Dinosauria: Second Edition. Berkeley: University of California Press. 2004. p. 325–34.

78. Norman DB, Sues HD, Witmer LM, Coria RA. Basal ornithopoda. In: Weishampel DB, Dodson P, Osmólska H, editors. The Dinosauria, Second Edition. Berkeley: University of California Press. 2004. p. 393–412.

79. Boyd CA, Brown CM, Scheetz RD, Clarke JA. Taxonomic revision of the basal neornithischian taxa *Thescelosaurus* and *Bugenasaura*. Journal of Vertebrate Paleontology. 2009;29(3):758–70. https://doi.org/10.1671/039.029.0328

80. Galton PM. Redescription of the skull and mandible of *Parksosaurus* from the Late Cretaceous. 1973.

81. Sues H-D, Evans DC, Galton PM, Brown CM. Anatomy of the neornithischian dinosaur *Parksosaurus warreni* (Parks, 1926) from the Upper Cretaceous (lower Maastrichtian) Horseshoe Canyon Formation of Alberta, Canada. Cretaceous Research. 2023;141:105369. https://doi.org/10.1016/j.cretres.2022.105369

82. Poole KE. Phylogeny and biogeography of iguanodontian dinosaurs, with implications from ontogeny and an examination of the function of the fused carpal-digit I complex. The George Washington University. 2015.

83. Varricchio DJ, Martin AJ, Katsura Y. First trace and body fossil evidence of a burrowing, denning dinosaur. Proc Biol Sci. 2007;274(1616):1361–8. https://doi.org/10.1098/rspb.2006.0443 PMID: 17374596

84. Dieudonné P-E, Cruzado-Caballero P, Godefroit P, Tortosa T. A new phylogeny of cerapodan dinosaurs. Historical Biology. 2020;33(10):2335–55. https://doi.org/10.1080/08912963.2020.1793979

85. Oelrich TM. The anatomy of the head of Ctenosaura pectinata (Iguanidae). 1956.

86. Martínez RN, Haro JA, Apaldetti C. Braincase ofPanphagia protos(Dinosauria, Sauropodomorpha). Journal of Vertebrate Paleontology. 2012;32(sup1):70–82. https://doi.org/10.1080/02724634.2013.819009

87. Sampson SD, Witmer LM. Craniofacial anatomy of *Majungasaurus crenatissimus* (Theropoda: Abelisauridae) from the late Cretaceous of Madagascar. Journal of Vertebrate Paleontology. 2007;27(sup2):32–104. https://doi.org/10.1671/0272-4634(2007)27[32:caomct]2.0.co;2

88. Sobral G, Müller J. The braincase of Mesosuchus browni (Reptilia, Archosauromorpha) with information on the inner ear and description of a pneumatic sinus. PeerJ. 2019;7:e6798. https://doi.org/10.7717/peerj.6798 PMID: 31198620

89. Sobral G, Sookias RB, Bhullar B-AS, Smith R, Butler RJ, Müller J. New information on the braincase and inner ear of Euparkeria capensis Broom: implications for diapsid and archosaur evolution. R Soc Open Sci. 2016;3(7):160072. https://doi.org/10.1098/rsos.160072 PMID: 27493766

90. Chapelle KEJ, Choiniere JN. A revised cranial description of Massospondylus carinatus Owen (Dinosauria: Sauropodomorpha) based on computed tomographic scans and a review of cranial characters for basal Sauropodomorpha. PeerJ. 2018;6:e4224. https://doi.org/10.7717/peerj.4224 PMID: 29340238

91. Galton PM, Kermack D. The anatomy of *Pantydraco caducus*, a very basal sauropodomorph dinosaur from the Rhaetian (Upper Triassic) of South Wales, UK. Revue de Paléobiologie. 2010;29(2):341–404.

92. Bruner HL. On the cephalic veins and sinuses of reptiles, with description of a mechanism for raising the venous blood-pressure in the head. Am J Anat. 1907;7(1):1–117. https://doi.org/10.1002/aja.1000070102

93. Bolotsky YL, Godefroit P. A new hadrosaurine dinosaur from the Late Cretaceous of Far Eastern Russia. Journal of Vertebrate Paleontology. 2004;24(2):351–65. https://doi.org/10.1671/1110

94. Godefroit P, Bolotsky YL, Lauters P. A new saurolophine dinosaur from the latest cretaceous of far Eastern Russia. PLoS One. 2012;7(5):e36849. https://doi.org/10.1371/journal.pone.0036849 PMID: 22666331

95. Maisch MW, Matzke AT. An isolated dinosaurian prootic with possible stegosaurian affinities (Dinosauria: Thyreophora) from the Upper Jurassic Qigu Formation of the southern Junggar Basin, NW-China. njgpa. 2019;294(3):275–83. https://doi.org/10.1127/njgpa/2019/0859

96. Butler RJ, Smith RMH, Norman DB. A primitive ornithischian dinosaur from the Late Triassic of South Africa, and the early evolution and diversification of Ornithischia. Proc Biol Sci. 2007;274(1621):2041–6. https://doi.org/10.1098/rspb.2007.0367 PMID: 17567562

97. Butler RJ, Upchurch P, Norman DB. The phylogeny of the ornithischian dinosaurs. Journal of Systematic Palaeontology. 2008;6(1):1–40. https://doi.org/10.1017/s1477201907002271

98. Butler RJ, Porro LB, Galton PM, Chiappe LM. Anatomy and cranial functional morphology of the small-bodied dinosaur *Fruitadens haagarorum* from the Upper Jurassic of the USA. PLoS One. 2012;7(4):e31556. https://doi.org/10.1371/journal.pone.0031556 PMID: 22509242

99. Edmund AG. On the special foramina in the jaws of many ornithischian dinosaurs. Contributions of the Royal Ontario Museum Division of Zoology and Palaeontology. 1957;48:1–14.

100. Norell MA, Barta DE. A New Specimen of the Ornithischian Dinosaur *Haya griva*, Cross-Gobi Geologic Correlation, and the Age of The Zos Canyon Beds. American Museum Novitates. 2016;3851(3851):1–20. https://doi.org/10.1206/3851.1

101. Rotatori FM, Moreno-Azanza M, Mateus O. New information on ornithopod dinosaurs from the Late Jurassic of Portugal. APP. 2020;65. https://doi.org/10.4202/app.00661.2019

102. Porro LB, Butler RJ, Barrett PM, Moore-Fay S, Abel RL. New heterodontosaurid specimens from the Lower Jurassic of southern Africa and the early ornithischian dinosaur radiation. Earth and Environmental Science Transactions of the Royal Society of Edinburgh. 2010;101(3–4):351–66. https://doi.org/10.1017/s175569101102010x

103. Galton PM. Cranial anatomy of the basal hypsilophodontid dinosaur *Thescelosaurus neglectus* Gilmore (Ornithischia: Ornithopoda) from the Upper Cretaceous of North America. Rev Paléobiol. 1997;16(1):231–58.

104. Holliday CM. New insights into dinosaur jaw muscle anatomy. Anat Rec (Hoboken). 2009;292(9):1246–65. https://doi.org/10.1002/ar.20982 PMID: 19711458

105. Xu X, Forster CA, Clark JM, Mo J. A basal ceratopsian with transitional features from the Late Jurassic of northwestern China. Proc Biol Sci. 2006;273(1598):2135–40. https://doi.org/10.1098/rspb.2006.3566 PMID: 16901832

106. Rauhut OWM. The interrelationships and evolution of basal theropod dinosaurs. Special Papers in Palaeontology. 2003;69:1–213.

107. Hudgins MN, Currie PJ, Sullivan C. Dental assessment of Stegoceras validum (Ornithischia: Pachycephalosauridae) and *Thescelosaurus neglectus* (Ornithischia: Thescelosauridae): paleoecological inferences. Cretaceous Research. 2022;130:105058. https://doi.org/10.1016/j.cretres.2021.105058

108. Coria RA, Calvo JO. A new iguanodontian ornithopod from Neuquen Basin, Patagonia, Argentina. Journal of Vertebrate Paleontology. 2002;22(3):503–9. https://doi.org/10.1671/0272-4634(2002)022[0503:aniofn]2.0.co;2

109. Galton PM. Teeth of ornithischian dinosaurs (mostly Ornithopoda) from the Morrison Formation (Upper Jurassic) of Western United States. Carpenter K, editor: Bloomington: Indiana University Press; 2007. 17–47 p.

110. Crompton AW, Charig AJ. A new Ornithischian from the Upper Triassic of South Africa. Nature. 1962;196(4859):1074–7. https://doi.org/10.1038/1961074a0

111. Godefroit P, Garcia G, Gomez B, Stein K, Cincotta A, Lefèvre U, et al. Extreme tooth enlargement in a new Late Cretaceous rhabdodontid dinosaur from Southern France. Sci Rep. 2017;7(1):13098. https://doi.org/10.1038/s41598-017-13160-2 PMID: 29074952

112. Bakker RT, Carpenter K, Galton PM, Siegarth J, Filla J. A new latest Jurassic vertebrate fauna, from the highest levels of the Morrison Formation at Como Bluff, Wyoming. Hunteria. 1990;2(6):1–20.

113. Barrett PM, Maidment SCR. A Review of Nanosaurus agilis Marsh and Other Small-Bodied Morrison Formation "Ornithopods". Bulletin of the Peabody Museum of Natural History. 2025;66(1). https://doi.org/10.3374/014.066.0102

114. Chen J, LeBlanc ARH, Jin L, Huang T, Reisz RR. Tooth development, histology, and enamel microstructure in Changchunsaurus parvus: Implications for dental evolution in ornithopod dinosaurs. PLoS One. 2018;13(11):e0205206. https://doi.org/10.1371/journal.pone.0205206 PMID: 30403689

115. Rauhut OWM. Dinosaur teeth from the Barremian of Uña, Province of Cuenca, Spain. Cretaceous Research. 2002;23(2):255–63. https://doi.org/10.1006/cres.2002.1003

116. Becerra MG, Pol D, Marsicano CA, Rauhut OWM. The dentition of *Manidens condorensis* (Ornithischia; Heterodontosauridae) from the Jurassic Cañadón Asfalto Formation of Patagonia: morphology, heterodonty and the use of statistical methods for identifying isolated teeth. Historical Biology. 2013;26(4):480–92. https://doi.org/10.1080/08912963.2013.794227

117. Rich TH, Rich P. Polar dinosaurs and biotas of the Early Cretaceous of southeastern Australia. National Geographic Research. 1989;5(1):15–53.

118. Agnolin FL, Ezcurra MD, Pais DF, Salisbury SW. A reappraisal of the Cretaceous non-avian dinosaur faunas from Australia and New Zealand: evidence for their Gondwanan affinities. Journal of Systematic Palaeontology. 2010;8(2):257–300. https://doi.org/10.1080/14772011003594870

119. Allison B, Lamaster T, Avrahami HM, Zanno LE. Buccal tooth development, replacement rate, and microstructure in Parksosauridae (Dinosauria: Neornithischia). Journal of Vertebrate Paleontology. 2019;2019(53):79.

120. Jung J, Kim M, Jo H, Clarke JA. A new dinosaur species from Korea and its implications for early-diverging neornithischian diversity. foss-rec. 2026;29(1):87–113. https://doi.org/10.3897/fr.29.178152

121. Maidment SCR, Butler RJ, Brusatte SL, Meade LE, Augustin FJ, Csiki-Sava Z, et al. A hidden diversity of ceratopsian dinosaurs in Late Cretaceous Europe. Nature. 2026;651(8105):397–403. https://doi.org/10.1038/s41586-025-09897-w PMID: 41501448

122. Maidment SCR, Barrett PM. Enigmacursor mollyborthwickae, a neornithischian dinosaur from the Upper Jurassic Morrison Formation of the western USA. R Soc Open Sci. 2025;12(6):242195. https://doi.org/10.1098/rsos.242195 PMID: 40568542
